# Synthesis, antiproliferative screening, and molecular docking of some heterocycles derived from *N*-(1-(5-chloro-3-methyl-1-phenyl-1*H*-pyrazol-4-yl)-3-hydrazineyl-3-oxoprop-1-en-2-yl)benzamide

**DOI:** 10.1038/s41598-025-27006-9

**Published:** 2025-11-24

**Authors:** Eman A. E. El-Helw, Youssef M. Youssef, Galal A. Elsayed, Amira A. El-Sayed, Aya I. Hassaballah, Ashraf M. Mohamed, Mohammad E. Azab

**Affiliations:** 1 Chemistry Department, Faculty of Science, Ain Shams University, Cairo, 11566 Egypt; 2 Applied Organic Chemistry Department, National Research Centre, Dokki, 12622 Egypt

**Keywords:** Antiproliferative, Imidazole, Pyrazolopyrazole, Pyrazole, Tetrazine, Biochemistry, Cancer, Chemical biology, Chemistry, Drug discovery

## Abstract

The hydrazide derivative synthesized from oxazolone was employed as a key building block for the preparation of various *N*-acetyl, *N*-benzoyl, imidazole, tetrazine, pyrazole, and pyrazolopyrazole compounds. These target heterocycles were obtained by reacting the hydrazide with several carbon-based electrophilic reagents, including chloroacetyl chloride, benzoyl chloride, acetic anhydride, carbon disulfide, and pyrazole aldehyde. The synthesized compounds were evaluated for their antiproliferative activity against colon (HCT-116) and breast (MCF-7) cancer cell lines, which implied the more selective toxicity of these derivatives toward cancer cell lines rather than normal cell line (WI-38), signifying the safety of the tested compounds. Biological screening results demonstrated that compounds **5**,** 8**,** 9**, and **10** exhibited significant cytotoxic activity against both cell lines. Among molecular docking simulation, the ligand binding energies were like those of doxorubicin (as an anticancer drug) and RRC (as a CDK2 inhibitor), as the most interacting amino acids were common, proposing being CDK2 inhibitor. The superlative docking score was given by compound **9** (S= -9.5080 kcal/mol), which was higher than that of doxorubicin and co-crystallized ligand (RRC). This work may develop the innovative, highly effective agents against cancer in the future.

**Supplementary Information:**

The online version contains supplementary material available at 10.1038/s41598-025-27006-9.

## Introduction

**Fig. 1:**
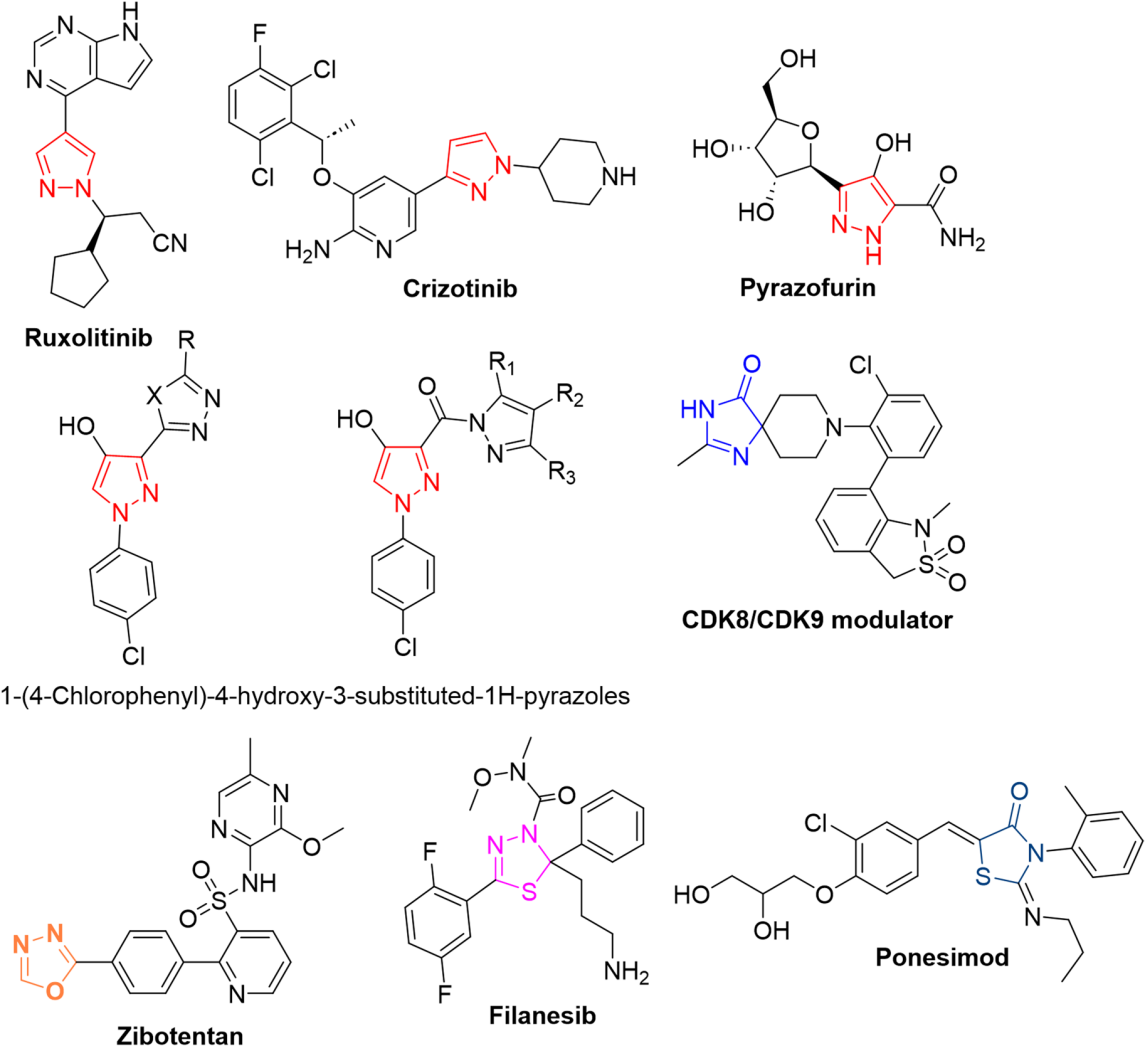
Some drugs bearing pyrazole, imidazoline, oxadiazole, thiadiazole, and thiazolidinone moieties.

Oxazolones have recently gained significant attention due to their feasible transformation into a wide range of heterocyclic systems like imidazolones, oxadiazoles, triazoles, thiadiazoles, thiazolidinones, and triazinones with promising pharmacological applications, including anticancer, antiviral, antimicrobial, antioxidant, and anti-inflammatory activities^1–5^. Imidazolones are found in pharmaceuticals, agrochemicals, fluorescent probes, and biological metabolites^6^. Oxadiazoles and thiadiazoles are versatile pharmacophores found in anticancer, antimicrobial, anti-inflammatory, and anti-tubercular agents^7^. For instance, 1,3,4-thiadiazole derivatives are integral in drugs like cephazolin and acetazolamide^8^. Thiazolidinones are known for their broad biological activity, especially in anticancer, antimicrobial, and antidiabetic domains^9^. Tetrazines exhibit potent anticancer, antibacterial, and antioxidant activities, including DNA cleavage capabilities^10^. Figure 1 displayed some drugs containing these promising heterocycles.

In turn, pyrazoles have drawn intense scientific interest owing to their diverse and potent physiological and pharmacological effects^11–13^ including antitumor, antiviral, antimicrobial, antioxidant, antidepressant, and anticonvulsant properties^14–20^. Notably, the US National Cancer Institute (NCI) has reported that 1-(4-chlorophenyl)−4-hydroxy-3-substituted pyrazoles (see Fig. 1) exhibit significant anticancer activity^12^. Additionally, certain pyrazole derivatives have demonstrated strong anticancer potential by inhibiting B-Raf kinase, with IC₅₀ values in the nanomolar range^21^.

As a result, the pyrazole ring is considered a valuable framework for developing pharmaceutical agents with diverse biological functions and favorable safety profiles. The impressive pharmacological performance of pyrazoles (cf. Figure 1) continues to inspire numerous research groups to design and synthesize heterocyclic compounds incorporating the pyrazolyl moiety. In this context and as a continuation of our strategy^22–29^, this work investigated the behavior of acid hydrazide **2**, derived from pyrazolyl-oxazolone **1**, towards different carbon electrophiles for constructing various heterocycles aimed at enhancing antiproliferative activity, particularly against colon and breast cancer cell lines.

## Results and discussion

### Synthesis

**Scheme 1:**
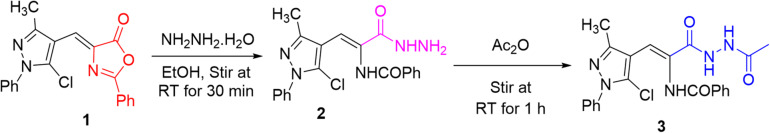
Synthesis of acid hydrazide **2** and its acylation with acetic anhydride.

 The requisite acid hydrazide **2** bearing a pyrazole moiety was successfully obtained in a good yield through stirring ethanolic solution of the oxazolone **1**^30^ with hydrazine hydrate at room temperature (RT) (Scheme 1). The IR spectrum of hydrazide **2** disclosed the absorption bands for NH_2_, NH, and amide C = O groups at ν 3296, 3233, 3199, and 1671 cm^− 1^, respectively, while that of lactone carbonyl group disappeared. The ^1^H NMR spectrum showed two broad singlet signals for two NH protons at δ 9.81 and 9.51 ppm and another broad singlet signal for NH_2_ protons at δ 4.39 ppm, in addition to a singlet signal for methyl protons at δ 2.10 ppm. Ample evidence for its structure was extended from its ^13^C NMR spectrum by showing signals at δ 165.6, 164.3, 137.5, 133.7, 131.7, 131.6, 129.2, 128.2, 128.1, 127.9, 125.5, 124.4, 114.8, 113.8, and 13.6 ppm. The acid hydrazide **2** was utilized to synthesize various heterocyclic systems of interesting pharmacological effects. Firstly, acylation of hydrazide **2** using acetic anhydride at room temperature furnished the mono-acetyl derivative **3** (Scheme 1). The IR spectrum exhibited a new band for acetyl C = O at ν 1703 cm^− 1^ and the ^1^H NMR spectrum exhibited two singlet signals attributable to the two methyl groups of the pyrazole and acetyl group at δ 2.12 and 1.89 ppm. Furthermore, its ^13^C NMR spectrum was completely consistent with the assigned structure.

**Scheme 2:**
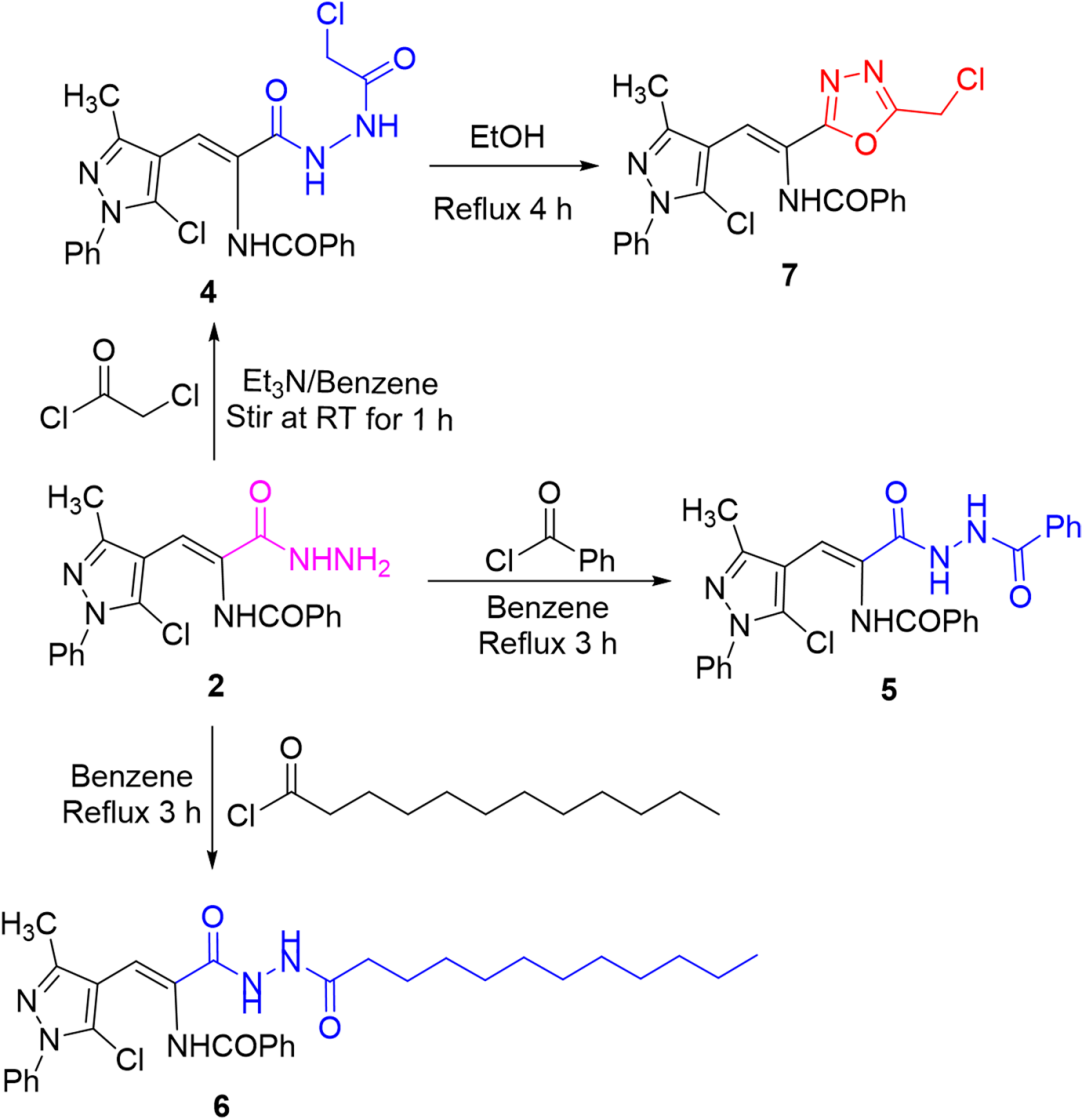
Reactions of hydrazide **2** with acid chlorides and synthesis of oxadiazole **7**.

**Scheme 3:**
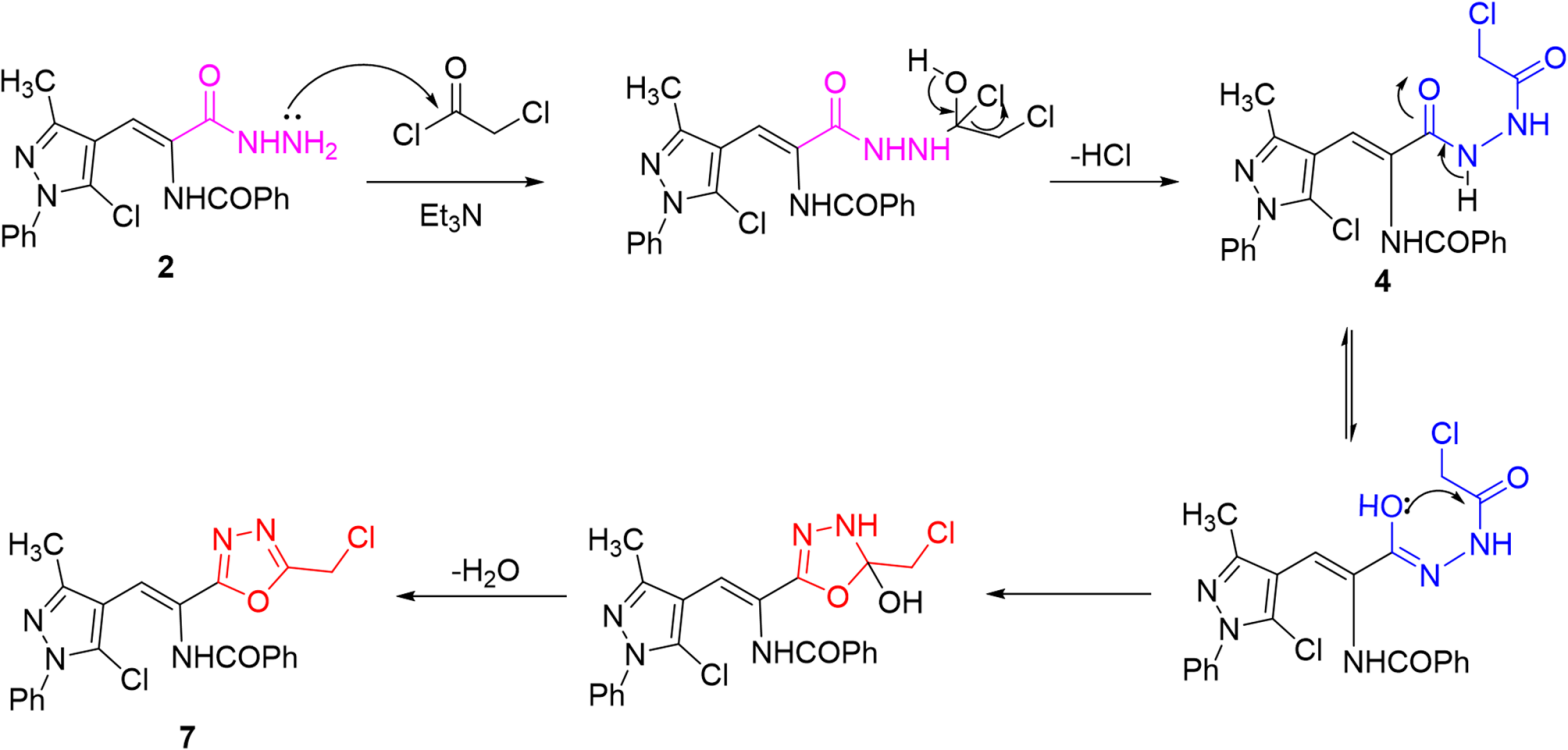
A suggested pathway for the formation of oxadiazole **7**.

Treating acid hydrazide **2** with different acid chlorides such as chloroacetyl chloride, benzoyl chloride, and dodecanoyl chloride yielded *N*-chloroacetyl hydrazide **4**, *N*-benzoyl hydrazide **5**, and *N*-dodecanoyl hydrazide **6**, respectively through the elimination of hydrogen chloride molecule (cf. Scheme 2). Refluxing *N*-chloroacetyl hydrazide **4** in ethanolic solution afforded oxadiazole candidate **7**, which can be postulated *via* Scheme 3. The spectral data confirmed the assigned structures of compounds obtained.

**Scheme 4:**
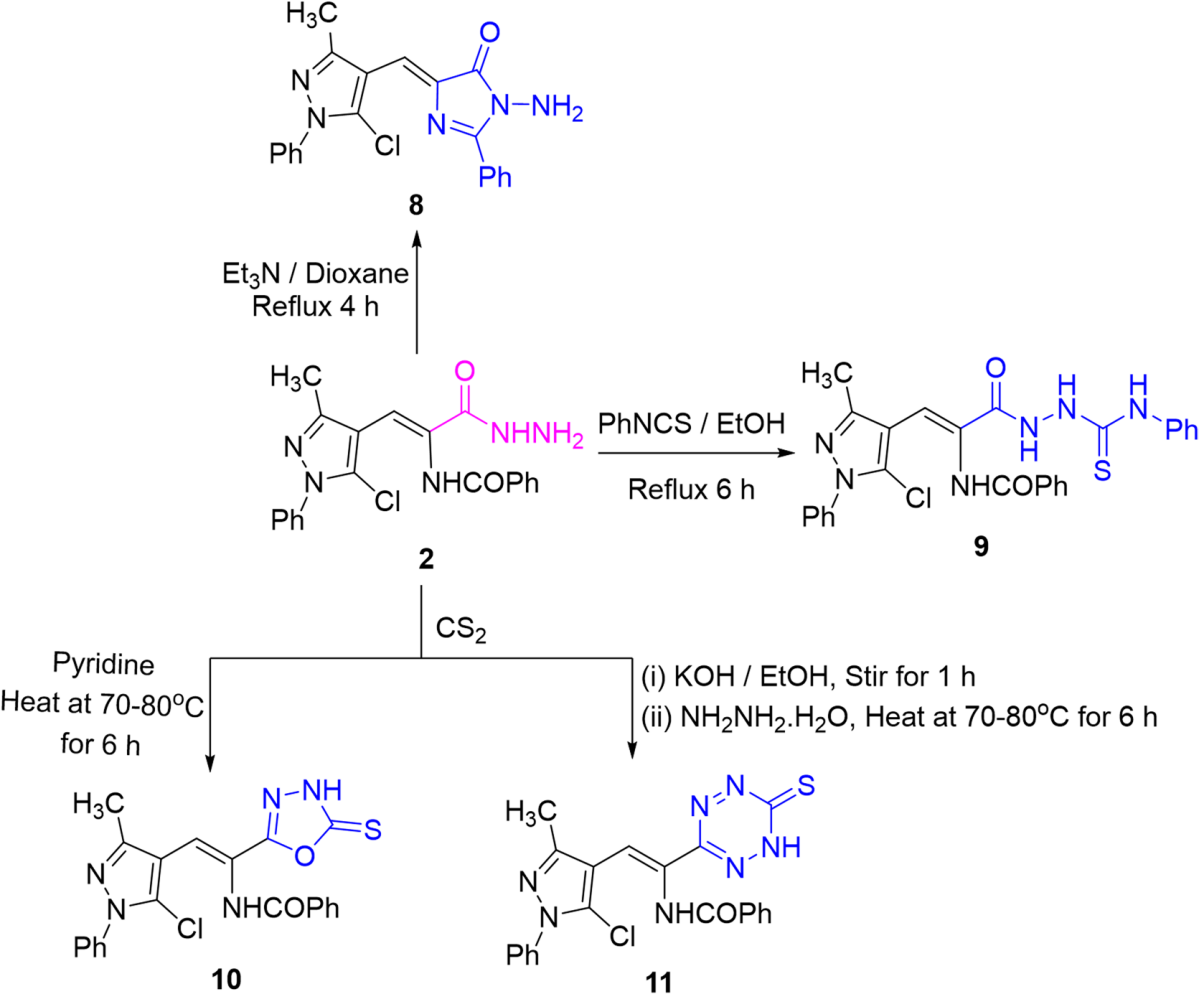
Synthesis of compounds **8–11**.

**Scheme 5:**
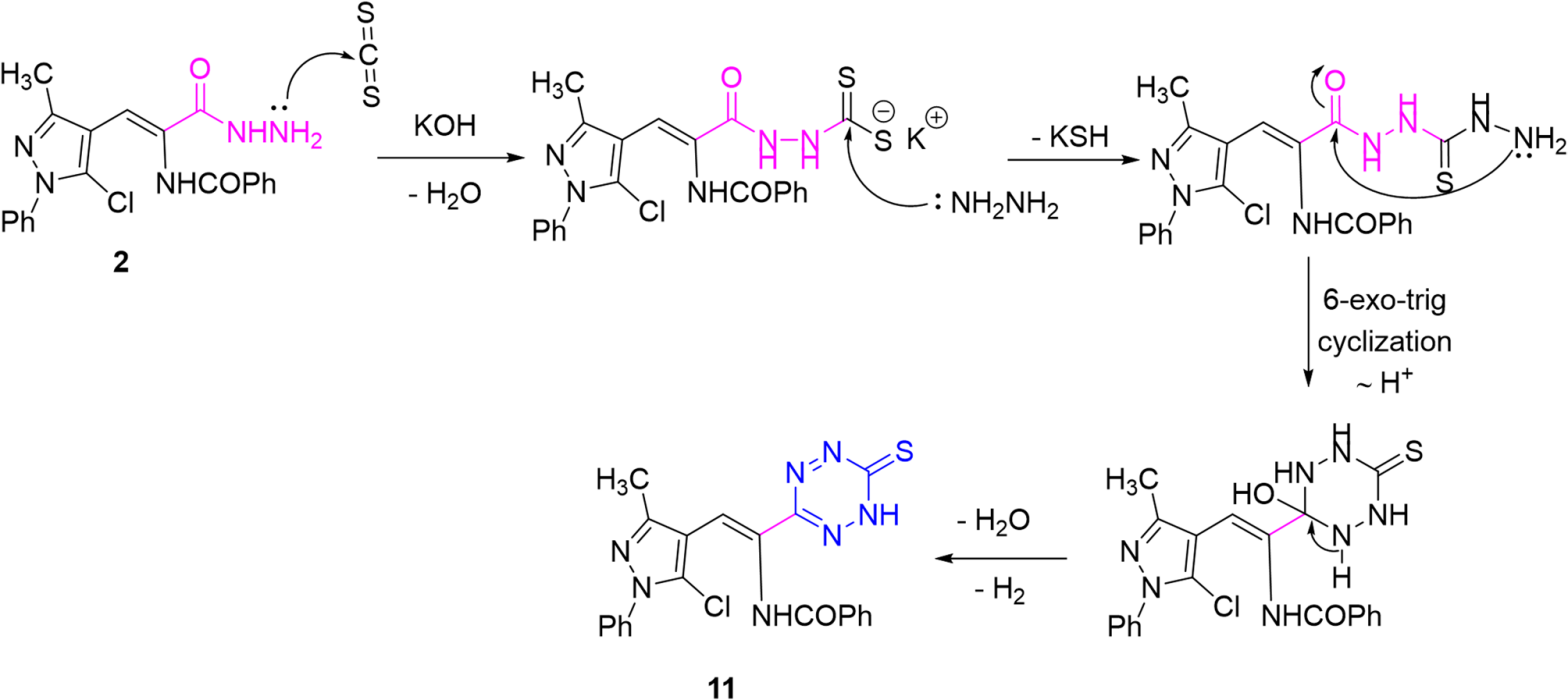
A plausible mechanism for the formation of tetrazinethione **11.**

On the other hand, *N*-aminoimidazolone **8** was obtained by refluxing the hydrazide **2** in dioxane and triethylamine through intramolecular 5-*exo*-trig cyclization. ^1^H NMR spectrum of imidazolone **8** exhibited one singlet signal for NH_2_ protons while the two NH signals disappeared. Refluxing the hydrazide **2** with phenyl isothiocyanate in ethanol yielded the thiosemicarbazide derivative **9** (Scheme 4). In turn, hydrazide **2** was submitted to react with carbon disulfide under two different conditions. Thus, heating the reaction mixture in pyridine at 70–80 °C yielded oxadiazolethione **10** while stirring the reaction mixture in ethanolic potassium hydroxide at ambient temperature followed by hydrazinolysis afforded the tetrazinethione **11**. The ^1^H NMR spectrum of oxadiazolethione **10** displayed two labile singlet signals for two NH protons. Presumably, the acyl hydrazide **2** was deprotonated by pyridine/KOH and attacked carbon atom of carbon disulfide to form a dithiocarbazate**-**type intermediate. That intermediate can undergo intramolecular cyclodehydration (condensation between the acyl carbonyl and the dithiocarbazate nitrogen/thiocarbonyl) to give a 1,3,4-oxadiazole-2-thione **10**^31^. Alternatively, in the presence of hydrazine (promote N–N bond formation and intermolecular condensation)^32^ two such nitrogen-rich fragments can couple and then cyclize/oxidize to a 1,2,4,5-tetrazinethione **11** (cf. Scheme 5).

**Scheme 6:**
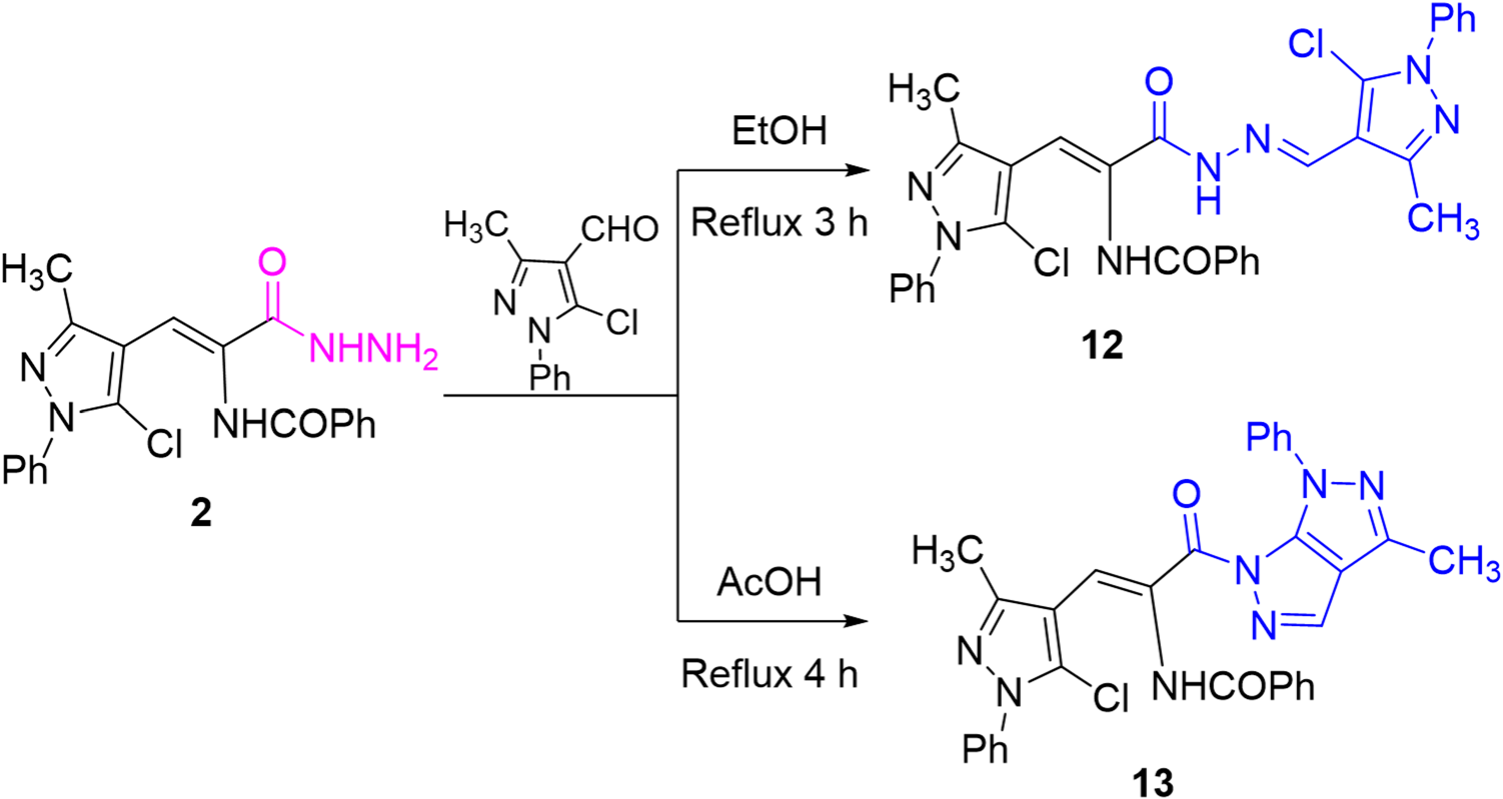
Condensation of hydrazide **2** with *N*-phenyl-5-chloro-4-formyl-3-methylpyrazole.

Noteworthy, condensation of **2** with *N*-phenyl-5-chloro-4-formyl-3-methylpyrazole in refluxing ethanol afforded the corresponding hydrazone derivative **12**. Carrying out the reaction in refluxing glacial acetic acid (higher boiling point solvent) achieved the construction of pyrazolopyrazole derivative **13** through initial condensation to give hydrazone **12**, which subsequently underwent 5-*endo*-trig cyclization by the attack of NH on C5-pyrazole to remove HCl molecule (Scheme 6). Their IR spectra showed absorption bands for NH and carbonyl groups. The ^1^H NMR spectrum of hydrazone **12** displayed a singlet signal for the new methyl protons, two singlet signals of two NH protons, and a singlet signal of methine (CH = N) proton. While the ^1^H NMR spectrum of pyrazolopyrazole **13** showed one broad singlet signal for NH proton. Their mass spectra supported the assigned structures through showing the correct molecular ion peaks.

**Scheme 7:**
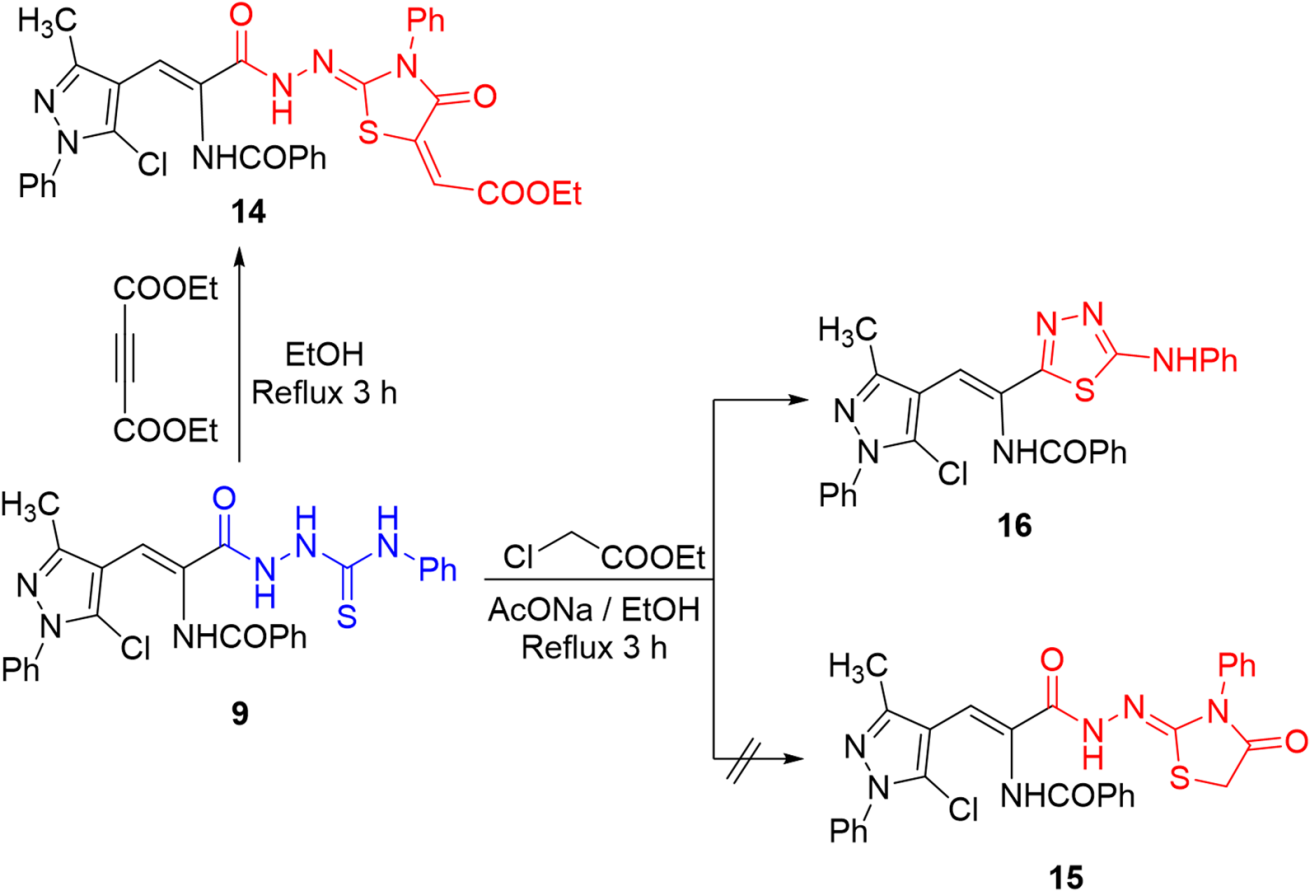
Reactions of thiosemicarbazide derivative **9** with diethyl acetylenedicarboxylate and ethyl chloroacetate.

In turn, refluxing thiosemicarbazide derivative **9** and diethyl acetylenedicaboxylate in ethanol afforded thiazolidinone derivative **14**. The IR spectrum showed a new absorption band for ester C = O at ν 1736 cm^− 1^ and its ^1^H NMR spectrum displayed two singlet signals for 2 NH groups at δ 11.41 and 9.99 ppm, in addition to triplet and quartet signals for protons of ethyl group. Finally, the reaction of thiosemicarbazide derivative **9** with ethyl chloroacetate failed to construct thiazolidinone **15** but afforded the thiadiazole derivative **16**, without inclusion of ethyl chloroacetate (Scheme 7). The IR spectrum of **16** showed an absorption band for the carbonyl group at ν 1678 cm^− 1^. In addition, its ^1^H NMR spectrum lacked the signal of methylene protons and offered two singlet signals for two NH protons at δ 10.53 and 10.29 ppm, which confirmed the assigned structure. Presumably, the formation of thiadiazole **16** was viewed through intramolecular 5-*exo*-trig cyclization by attack of thione-sulfur (C = S) on the carbonyl-carbon (C = O) atom to eliminate water molecule. This reaction pathway was also chemically confirmed by executing the reaction without ethyl chloroacetate, and the evolution of hydrogen sulfide was not detected using lead acetate paper, confirming the attack by sulfur atom to remove water molecule^33^.

## Antiproliferative activity

The synthesized compounds were screened for their in vitro antiproliferative activity against two human tumor cell lines, namely, colon carcinoma (HCT-116) and breast cancer (MCF-7), in addition to their cytotoxicity against the normal human lung fibroblast (WI-38) cell line using MTT assay^34^. The results in Table 1 disclosed that derivatives **5**, **8**, **9**, and **10** showed strong efficacy. Meanwhile, the *N*-chloroacetylhydrazide **4** exhibited moderate efficacy against the HCT-116 while strong activity against MCF-7 cell line. The hydrazone **12** and pyrazolopyrazole **13** showed no activity at all against HCT-116, but they had weak activity against MCF-7 cell line. The rest of the tested compounds offered weak activities. All the tested substances exhibited a low toxicity on the non-cancerous cell line (WI-38). The selectivity of tested derivatives against two cancer cell lines was evaluated using the normal cell line. The selectivity index (SI) values, commonly applied in pharmacology and toxicology to judge the relative safety or specificity of a compound, were calculated in Table 1. Compounds **9**, **10**, **5**, and **8** show the highest selectivity toward cancer cells, particularly MCF-7. SI > 3 generally implies good selectivity toward tumor cells.

**Fig. 2:**
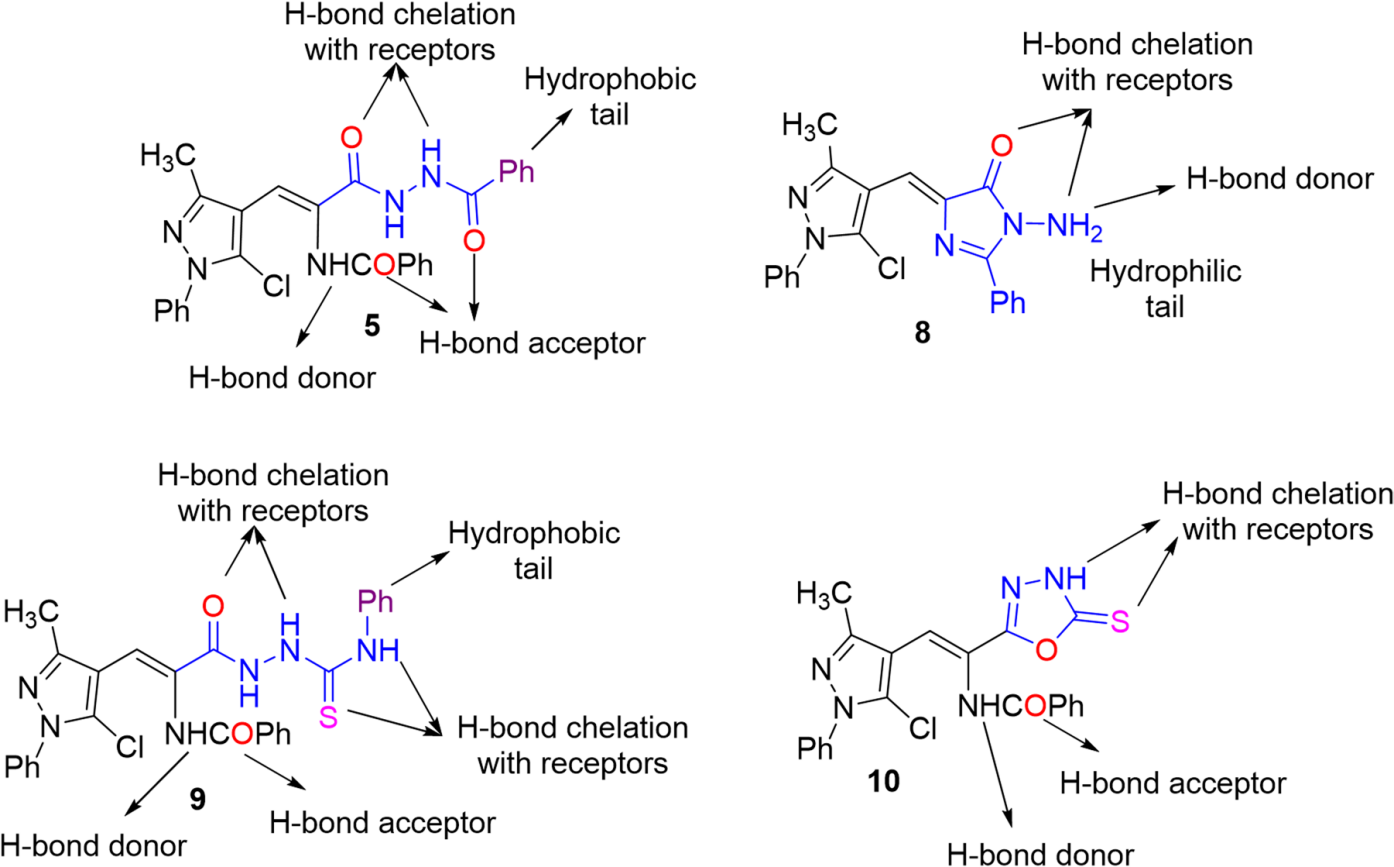
SAR of the potent compounds.

A structure-activity relationship (SAR) study of the potent compounds **5**, **8**, **9**, and **10** indicated the promising role of structures bearing NHCO, NH_2_, and NHCS functionalities in the biological profile, because they are capable of hydrogen bonding with receptors^35,36^. Also, the existence of imidazolone (compound **8**) and oxadiazolethione (compound **10**) moieties bearing suitable positions of functional groups enhanced the activity through chelating hydrogen bonding with receptors (cf. Figure 2). Compound **5** has a primary amino group capable of strong chelating hydrogen bonding with amino acid receptors of the target protein. Thiosemicarbazide derivative **9** has many NH groups in addition to oxo- and thioxo- groups, forming extra hydrogen bonding with receptors. Compounds **5** and **9** had a phenyl group as a hydrophobic tail, capable of hydrophobic interaction with receptors (cf. Figure 2). According to the electronic effects, they are more polarized and readily give electrons to receptors, making them promising candidates for bioactivity. These compounds’ longer conjugation led to greater affinity for creating face-to-edge aromatic contact with the receptor’s active pockets. For more details about the mode of antiproliferative action of these compounds, a molecular docking simulation was performed in the next section.

**Table 1:** Cytotoxic activity of the tested compounds against human tumor cell lines compared to normal cell line^a^.

Substance	In vitro Cytotoxicity (IC_50_, µM)^b^			SI^c^	
	HCT-116	MCF-7	WI-38	HCT-116	MCF-7
**1**	73.50 ± 3.8	59.31 ± 3.3	> 100	1.36	1.69
**2**	42.72 ± 2.7	11.91 ± 1.0	72.23 ± 1.4	1.69	6.06
**3**	89.27 ± 4.7	71.83 ± 3.8	> 100	1.12	1.39
**4**	26.84 ± 1.9	19.04 ± 1.6	87.12 ± 1.4	3.25	4.58
**5**	10.36 ± 0.8	17.23 ± 1.4	74.52 ± 1.3	7.19	4.33
**6**	93.87 ± 4.9	68.66 ± 3.6	> 100	1.07	1.46
**7**	86.61 ± 4.4	82.97 ± 4.1	> 100	1.15	1.21
**8**	21.81 ± 1.6	14.75 ± 1.3	79.87 ± 1.5	3.66	5.41
**9**	13.89 ± 1.0	7.65 ± 0.5	71.24 ± 0.9	5.13	9.31
**10**	15.65 ± 1.1	9.26 ± 0.8	82.23 ± 1.2	5.25	8.88
**11**	49.25 ± 2.9	32.45 ± 2.3	89.78 ± 2.5	1.82	2.77
**12**	> 100	78.12 ± 3.9	> 100	1.00	1.28
**13**	> 100	87.72 ± 4.5	> 100	1.00	1.14
**14**	53.72 ± 3.0	39.36 ± 2.4	95.12 ± 3.4	1.77	2.42
**16**	59.62 ± 3.3	48.03 ± 2.9	98.52 ± 3.0	1.65	2.05
**Doxorubicin**	5.23 ± 0.3	4.17 ± 0.2	10.36 ± 0.5	1.98	2.48

^a^ The period of incubation of cells with the examined compounds was 24 h. ^**b**^ **IC**_**50**_ **(µM)**: 1–10 (very strong), 11–20 (strong), 21–50 (moderate), 51–100 (weak), > 100 (non-cytotoxic). ^c^ SI = IC_50_ of normal cell line/IC_50_ of respective cancer cell line.

## Molecular Docking

To determine the antiproliferative mode of action, a molecular docking simulation of the potent compounds **5**, **8**, **9**, and **10** was performed towards the cyclin-dependent kinase-2 (CDK2) protein (PDB ID: 2A4L)^37,38^, and the binding affinity (S-score, kcal/mol) was assessed (cf. Tables 2 and 3). CDK2 was selected as the target protein because of its well-established role in regulating the cell cycle, particularly the G1-to-S phase transition. Overexpression or dysregulation of CDK2 has been strongly associated with uncontrolled cellular proliferation in various cancers, making it a promising therapeutic target. Several studies have demonstrated that inhibition of CDK2 can induce cell cycle arrest and apoptosis in cancer cells while sparing normal cells, which aligns with the aim of our study to identify potential anticancer agents. Specifically, CDK2 protein was also selected because the studied molecules possess structural features compatible with kinase inhibition, particularly those that can interact with the ATP-binding pocket of CDK2.

The amino acids of target CDK2 protein involved in binding interactions with the prepared ligands were listed in Table 2. The 2D and 3D interactions of the docking simulation were shown in Table 3. The superlative docking score was shown by compound **9** (S= −9.5080 kcal/mol), which was higher than that of doxorubicin (as an anticancer drug) and co-crystallized ligand (RRC) as a CDK2 inhibitor. This was through the formation of two hydrogen bonds with THR 14 and LYS 129, in addition to pi-hydrogen contact with LYS 89 (common with RRC). The other ligand binding energies were like those of doxorubicin and RRC, as the most interacting amino acids were common, proposing being CDK2 inhibitor. Compound **5** formed one hydrogen bonding with ASP 86 and three pi-hydrogen contacts with ILE 10, VAL 18, and GLN 131, in addition to pi-cation interaction with LYS 89. Compound **10** (S= −8.1894 kcal/mol) formed one H-bond with LYS 89 and two pi-H contacts with VAL 18 and ALA 144. Molecular docking and SAR analyses indicated that the studied compounds contain pharmacophoric moieties capable of forming key hydrogen bonds and hydrophobic interactions with the active-site residues of CDK2 (such as Leu83, Glu81, and Asp86), similar to known CDK2 inhibitors. Therefore, CDK2 was chosen as a relevant biological target to evaluate the potential anticancer activity of the designed molecules.

**Fig. 3:**
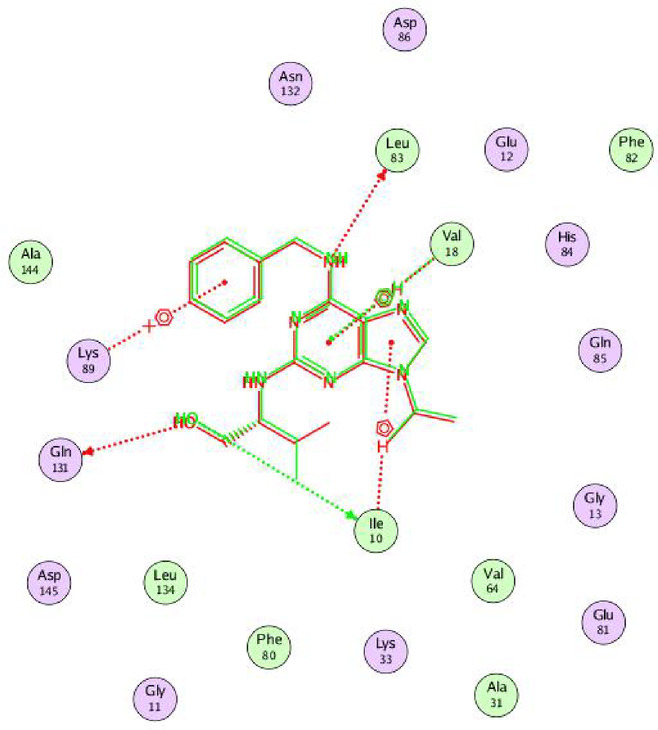
2D diagrams of the superimposition of the native and redocked co-crystallized ligand (RRC) structures at CDK2 protein target (PDB ID: 2A4L) with RMSD value of 1.4404 Å.

The validation of docking protocol was endorsed by employing co-crystallized ligands (RRC) with their respective protein target, wherein the superimposition of the native and redocked co-crystallized ligands was visualized through 2D diagram^38,39^. The RMSD value for the superimposition, which implies the difference between two structures, was determined to be 1.4404 Å (cf. Figure 3).

**Table 2:** Docking results of the potent compounds toward CDK2 protein (PDB ID: 2A4L) compared to co-crystallized ligand (RRC) and reference drug (doxorubicin).

Compds.	S score (kcal/mol)	RMSD (Å)	Amino acids*	Type of interaction	Bond length (Å)
**5**	−8.2037	1.9130	ASP 86 *ILE 10* *VAL 18* *LYS 89* *GLN 131*	H-donor pi-H pi-H pi-cation pi-H	3.08 4.50 4.47 3.30 3.81
**8**	−7.1747	1.5602	*LYS 89* PHE 80 *VAL 18* *GLN 131*	H-acceptor H-pi pi-H pi-H	2.87 4.48 3.96 3.99
**9**	−9.5080	1.6345	THR 14 LYS 129 *LYS 89*	H-acceptor H-acceptor pi-H	3.39 3.40 3.85
**10**	−8.1894	1.4738	*LYS 89* *VAL 18* ALA 144	H-acceptor pi-H pi-H	3.31 3.63 4.21
**RRC**	−8.7658	1.4404	*GLN 131* LEU 83 *ILE 10* *VAL 18* *LYS 89*	H-donor H-donor pi-H pi-H pi-cation	2.77 3.31 4.21 4.48 3.87
**Doxorubicin**	−8.6768	1.3264	GLU 12 *VAL 18* *GLN 131*	H-acceptor pi-H pi-H	3.28 4.20 4.26

*Common amino acids interacting with potent compounds, co-crystallized ligand, and doxorubicin were italized.

**Table 3:** 2D and 3D interactions of the potent compounds toward CDK2 protein (PDB ID: 2A4L) compared to co-crystallized ligand (RRC) and reference drug (doxorubicin).

Compds.	2D interactions	3D interactions*
5		
8		
**9**		
**10**		
**RRC**		
**Doxorubicin**		

*The docked compounds were colored in cyan, while RRC and doxorubicin were colored in orange and green, respectively.

## Conclusion

In summary, a pyrazole-based hydrazide was utilized as a scaffold to synthesize a series of new heterocyclic derivatives *via* its reaction with some carbon electrophiles. The in vitro antiproliferative activity of the synthesized compounds was assessed against colon (HCT-116) and breast (MCF-7) cancer cell lines, in addition to cytotoxicity against normal cell line (WI-38). From the biological results, *N*-benzoylhydrazide, *N*-aminoimidazolone, phenylthiosemicarbazide, and oxadiazolethione derivatives were the most potent against the two cell lines. The results indicated the more selective toxicity of these derivatives toward cancer cell lines rather than normal cell line, showing the safety of the tested compounds. A molecular docking simulation was performed toward CDK2 protein to determine the mode of action, which showed the best docking score of phenylthiosemicarbazide derivative compared to doxorubicin (as an anticancer drug) and co-crystallized ligand (roscovitine as a CDK2 inhibitor). This work may contribute to developing new effective antiproliferative drugs.

## Materials and methods

All melting points were measured on GALLENKAMP melting point apparatus. The IR spectra (ν, cm^−1^) were recorded on a Pye-Unicam SP-3–300 infrared spectrophotometer (KBr disks). ^1^H and ^13^C NMR spectra (δ, ppm) were run at 300 and 100 *MHz* on a Varian Mercury VX-300 and Bruker Advance III-400 NMR spectrometer, respectively. TMS was used as an internal standard. Deuterated dimethyl sulfoxide (DMSO-*d*_*6*_) was used as the solvent. The mass spectra were recorded on Shimadzu GCMS-QP-1000EX mass spectrometer at 70 eV. Elemental analyses were performed on CHN analyzer, and all compounds were within ± 0.4 of the theoretical values. The reactions were monitored by thin-layer chromatography (TLC) using TLC sheets coated with UV fluorescent silica gel Merck 60 F_254_ plates and were visualized using UV lamp and different solvents as mobile phases. All reagents and solvents were purified and dried by standard techniques.

### N-(1-(5-Chloro-3-methyl-1-phenyl-1H-pyrazol-4-yl)−3-hydrazineyl-3-oxoprop-1-en-2-yl) benzamide (2)

A solution of oxazolone **1**^30^ (3.63 g, 0.01 mol) and hydrazine hydrate (1 mL, 0.02 mol, 80%) in ethanol (30 mL) was stirred for 30 min at room temperature. The separated product was filtered and crystallized from ethanol to give white crystals. Yield 83%, mp. 182–184 °C. IR (ν, cm^−1^): 3296, 3233, 3199 (NH_2_, NH), 1671 (C = O), 1646 (C = N). ^1^H NMR (DMSO-*d*_6_, *δ*, ppm): 9.81 (*br*.s, 1H, *NH*CO, exchangeable), 9.51 (*br*.s, 1H, *NH*NH_2_, exchangeable), 7.98–7.44 (m, 10 H, Ar-H), 6.66 (s, 1H, CH=), 4.39 (*br*.s, 2 H, NH_2_, exchangeable), 2.10 (s, 3 H, CH_3_). ^13^C NMR (DMSO-*d*_6_): 165.6, 164.3,148.1 137.5, 133.7, 131.7, 131.6, 129.2,129.0, 128.2, 128.1, 127.9(2), 125.5,124.5, 124.4, 114.8(2), 113.8, 13.6. GCMS (*m/z*, %): 395 (M^+^., 54), 367 (60), 351 (51), 343 (43), 321 (35), 306 (29), 281 (69), 208 (24), 180 (100), 107 (46), 65 (54). Anal.: calcd. for C_20_H_18_ClN_5_O_2_ (395.85): C: 60.69, H: 4.58, N: 17.69, found; C: 60.75, H: 4.70, N: 17.82.

## N-(3-(2-Acetylhydrazineyl)−1-(5-chloro-3-methyl-1-phenyl-1 H-pyrazol-4-yl)−3-oxoprop-1-en-2-yl)benzamide (3)

A suspension of hydrazide **2** (3.95 g, 0.01 mol) and acetic anhydride (10 mL) was stirred at room temperature for 1 h. The formed solid was filtered and recrystallized from ethanol to give white crystals. Yield 71%, mp. 188–190 °C. IR (ν, cm^− 1^): 3305, 3226 (NH), 1704, 1674 (C = O), 1647 (C = N). ^1^H NMR (DMSO-*d*_6_, *δ*, ppm): 10.06, 10.00, 9.89 (*br*.s, 3 H, 3NH, exchangeable), 7.98–7.52 (m, 10 H, Ar-H), 6.78 (s, 1H, CH=), 2.12 (s, 3 H, CH_3_), 1.89 (s, 3 H, CH_3_CO). ^13^C NMR (DMSO-*d*_6_): 174.6, 168.2, 165.8, 163.9, 148.2, 137.5, 133.6, 131.7, 131.38, 129.3 (2), 128.4 (2), 128.2, 127.9, 125.8, 124.5, 116.0, 115.7, 113.7, 20.6, 13.7. GCMS (*m/z*, %): 437 (M^+^., 86), 425 (91), 409 (49), 395 (24), 341 (37), 275 (43), 268 (100), 244 (37), 177 (31), 132 (36), 91 (27). Anal.: calcd. for C_22_H_20_ClN_5_O_3_ (437.88): C: 60.35, H: 4.60, N: 15.99, found; C: 60.55, H: 4.71, N: 16.07.

## N-(1-(5-Chloro-3-methyl-1-phenyl-1H-pyrazol-4-yl)−3-(2-(2-chloroacetyl)hydrazinyl)−3-oxoprop-1-en-2-yl)benzamide (4)

The hydrazide **2** (3.95 g, 0.01 mol) was stirred in dry benzene (20 mL) and triethylamine (0.5 mL) with chloroacetyl chloride (1.12 mL, 0.01 mol) for 1 h at room temperature. The solid deposited was filtered, washed with light petroleum ether, and recrystallized from benzene to furnish beige crystals. Yield 74%, mp. 122–124 °C. IR (ν, cm^−1^): 3278, 3220 (NH), 1731, 1686, 1663 (C = O). ^1^H NMR (DMSO-*d*_6_, *δ*, ppm): 10.38 (*br*.s, 1H, *NH*NHCO, exchangeable),10.30 (*br*.s, 1H, *NH*COCH_2_, exchangeable), 9.91 (*br*.s, 1H, NH, exchangeable), 7.98–7.46 (m, 10 H, Ar-H), 6.76 (s, 1H, CH=), 4.26 (s, 2 H, CH_2_), 2.11 (s, 3 H, CH_3_). Anal.: calcd. for C_22_H_19_Cl_2_N_5_O_3_ (472.33): C: 55.94, H: 4.05, N: 14.83, found; C: 55.65, H: 3.97, N: 14.61.

### N-(3-(2-Benzoylhydrazinyl)−1-(5-chloro-3-methyl-1-phenyl-1 H-pyrazol-4-yl)−3-oxoprop-1-en-2-yl)benzamide (5)

A solution of hydrazide **2** (3.95 g, 0.01 mol) and benzoyl chloride (1.4 mL, 0.01 mol) in dry benzene (20 mL) was refluxed for 3 h. The reaction mixture was cooled and the separated solid was filtered, washed with light petroleum ether, and then recrystallized from benzene to afford pale**-**yellow crystals. Yield 65%, mp. 98–100 °C. IR (ν, cm^−1^): 3238 (NH), 1697, 1650 (C = O). ^1^H NMR (DMSO-*d*_6_, *δ*, ppm): 10.50, 10.26, 9.94 (*br*.s, 3 H, 3NH, exchangeable), 8.01–7.35 (m, 15 H, Ar-H), 6.89 (s, 1H, CH=), 2.14 (s, 3 H, CH_3_). GCMS (*m/z*, %): 499 (M^+^., 19), 481 (36), 474 (55), 449 (25), 427 (24), 369 (17), 303 (25), 246 (25), 223 (22), 122 (100), 109 (32), 84 (25). Anal.: calcd. for C_27_H_22_ClN_5_O_3_ (499.96): C: 64.87, H: 4.44, N: 14.01, found; C: 64.81, H: 4.29, N: 13.92.

### N-(1-(5-Chloro-3-methyl-1-phenyl-1H-pyrazol-4-yl)−3-(2-dodecanoylhydrazinyl)−3-oxoprop-1-en-2-yl)benzamide (6)

A solution of hydrazide **2** (3.95 g, 0.01 mol) and dodecanoyl chloride (2.19 mL, 0.01 mol) in dry benzene (20 mL) was refluxed for 3 h. The reaction mixture was cooled and the separated solid was filtered, washed with light petroleum ether, then recrystallized from benzene to afford yellow crystals. Yield 65%, mp. 76–78 °C. IR (ν, cm^−1^): 3231 (NH), 1699 (C = O), 1650 (C = N). ^1^H NMR (DMSO-*d*_6_, *δ*, ppm): 10.02, 9.86, 9.80 (*br*.s, 3 H, 3NH, exchangeable), 7.98–7.03 (m, 10 H, Ar-H), 6.76 (s, 1H, CH=), 2.69 (t, 2 H, CH_2_CO, *J* = 7.5 *Hz*), 2.13 (s, 3 H, CH_3_), 1.52–1.18 (m, 18 H, 9CH_2_), 0.84 (t, 3 H, *CH*_*3*_CH_2_, *J* = 7.8 *Hz*). Anal.: calcd. for C_32_H_40_ClN_5_O_3_ (578.15): C: 66.48, H: 6.97, N: 12.11, found; C: 66.41, H: 6.90, N: 12.01.

### N-(2-(5-Chloro-3-methyl-1-phenyl-1H-pyrazol-4-yl)−1-(5-(chloromethyl)−1,3,4-oxadiazol-2-yl)vinyl)benzamide (7)

A solution of derivative **4** (5.47 g, 0.01 mol) in absolute ethanol (20 mL) was refluxed for 4 h. The obtained solid was filtered and crystallized from dioxane to produce yellow crystals. Yield 70%, mp. 220–222 °C. IR (ν, cm^−1^): 3310 (NH), 1659 (C = O). ^1^H NMR (DMSO-*d*_6_, *δ*, ppm): 9.76 (*br*.s, 1H, NH, exchangeable), 7.99–7.45 (m, 10 H, Ar-H), 6.05 (s, 1H, CH=), 4.05 (s, 2 H, CH_2_), 2.26 (s, 3 H, CH_3_). GCMS (*m/z*, %): 453 (M^+^., 47), 426 (49), 386 (40), 363 (71), 301 (39), 241 (63), 173 (100), 167 (47), 103 (49), 41 (36). Anal.: calcd. for C_22_H_17_Cl_2_N_5_O_2_ (454.31): C: 58.16, H: 3.77, N: 15.42, found; C: 58.08, H: 3.59, N: 15.32.

***3-Amino-5-((5-chloro-3-methyl-1-phenyl-1 H-pyrazol-4-yl)methylene)−2-phenyl-3***,***5-dihydro-4 H-imidazol-4-one (8)***.

A solution of the hydrazide **2** (5.47 g, 0.01 mol) in dioxane (20 mL) and triethylamine (1 ml) was refluxed for 4 h. The formed solid was filtered on hot and crystallized from dioxane to furnish dark-green crystals. Yield 85%, mp. 204–206 °C. IR (ν, cm^−1^): 3307, 3213 (NH_2_), 1703 (C = O), 1636 (C = N). ^1^H NMR (DMSO-*d*_6_, *δ*, ppm): 8.36–7.51 (m, 10 H, Ar-H), 6.96 (s, 1H, CH=), 5.38 (*br*.s, 2 H, NH_2_, exchangeable), 2.68 (s, 3 H, CH_3_). GCMS (*m/z*, %): 377 (M^+^., 25), 365 (100), 355 (55), 322 (74), 281 (80), 233 (88), 213 (46), 192 (70), 164 (54), 83 (85), 59 (67). Anal.: calcd. for C_20_H_16_ClN_5_O (377.83): C: 63.58, H: 4.27, N: 18.54, found; C: 63.68, H: 4.29, N: 18.64.

### N-(1-(5-Chloro-3-methyl-1-phenyl-1 H-pyrazol-4-yl)−3-oxo-3-(2-(phenylcarbamothioyl)-hydrazinyl)prop-1-en-2-yl)benzamide (9)

A solution of hydrazide **2** (3.95 g, 0.01 mol) and phenyl isothiocyanate (1.35 mL, 0.01 mol) in ethanol (20 mL) was refluxed for 6 h. The product was collected and recrystallized from dioxane to give white crystals. Yield 77%, mp. 190–192 °C. IR (ν, cm^−1^): 3378, 3206, 3180, 3128 (NH), 1678 (C = O), 1646 (C = N). ^1^H NMR (DMSO-*d*_6_, *δ*, ppm): 10.58, 10.41, 9.80, 9.34 (*br*.s, 4 H, 4NH, exchangeable), 8.03–7.16 (m, 15 H, Ar-H), 6.75 (s, 1H, CH=), 2.12 (s, 3 H, CH_3_). GCMS (*m/z*, %): 530 (M^+^., 31), 519 (68), 512 (43), 465 (35), 422 (34), 379 (41), 328 (47), 277 (60), 270 (100), 204 (49), 184 (63), 160 (99). Anal.: calcd. for C_27_H_23_ClN_6_O_2_S (531.03): C: 61.07, H: 4.37, N: 15.83, found; C: 61.02, H: 4.29, N: 15.79.

### N-(2-(5-Chloro-3-methyl-1-phenyl-1H-pyrazol-4-yl)−1-(5-thioxo-4,5-dihydro-1,3,4-oxadiazol-2-yl)vinyl)benzamide (10)

A mixture of acid hydrazide **2** (3.95 g, 0.01 mol) and carbon disulfide (5 mL) was heated in pyridine (20 mL) at 70–80 °C for 6 h. Acidification of the reaction mixture by cold dilute hydrochloric acid (10%) deposited a semi-solid product which was filtered, dried, and recrystallized from ethanol to furnish beige crystals. Yield 60%, mp. 231–233 °C. IR (ν, cm^− 1^): 3233 (NH), 1642 (C = O). ^1^H NMR (DMSO-*d*_6_, *δ*, ppm): 14.70 (*br*.s, 1H, NHCS, exchangeable), 10.33 (*br*.s, 1H, NH, exchangeable), 7.98–7.44 (m, 10 H, Ar-H), 6.90 (s, 1H, CH=), 2.18 (s, 3 H, CH_3_). ^13^C NMR (DMSO-*d*_6_): 177.3, 166.1, 159.6, 148.6, 137.3, 132.8, 132.2, 129.3, 128.5(2), 127.7(2), 126.4(2), 124.6(2), 120.5(2), 117.7, 113.1, 13.6. Anal.: calcd. for C_21_H_16_ClN_5_O_2_S (437.90): C: 57.60, H: 3.68, N: 15.99, found; C: 57.56, H: 3.52, N: 15.79.

### N-(2-(5-Chloro-3-methyl-1-phenyl-1 H-pyrazol-4-yl)−1-(6-thioxo-1,6-dihydro-1,2,4,5-tetrazin-3-yl)vinyl)benzamide (11)

To a mixture of potassium hydroxide (0.01 mol in 2 mL of water) in ethanol (15 mL), hydrazide **2** (3.95 g, 0.01 mol) was added, then the mixture was cooled in ice bath with stirring for 10 min. After that, carbon disulfide (5 mL) was added dropwise. After 1 h, hydrazine hydrate (0.02 mol, 80%) was added, and the reaction mixture was heated on water bath at 70–80 °C for 6 h. After cooling, the reaction mixture was acidified with dilute HCl (10%). The precipitate was filtered and recrystallized from benzene to obtain simony crystals. Yield 50%, mp. 120–122 °C. IR (ν, cm^− 1^): 3331 (br. NH), 1712 (C = O), 1641 (C = N). ^1^H NMR (DMSO-*d*_6_, *δ*, ppm): 9.86 (*br*.s, 1H, *NH*CS, exchangeable), 8.77 (*br*.s, 1H, *NH*CO, exchangeable), 7.85–7.35 (m, 10 H, Ar-H), 6.98 (s, 1H, CH=), 2.21 (s, 3 H, CH_3_). GCMS (*m/z*, %): 449 (M^+^., 38), 410 (43), 370 (71), 303 (72), 261 (100), 252 (87), 241 (56), 204 (51), 172 (49), 153 (66), 140 (93). Anal.: calcd. for C_21_H_16_ClN_7_OS (449.92): C: 56.06, H: 3.58, N: 21.79, found; C: 56.08, H: 3.64, N: 21.82.

### N-(>−1-(5-Chloro-3-methyl-1-phenyl-1H-pyrazol-4-yl)−3-(2-((5-chloro-3-methyl-1-phenyl-1H-pyrazol-4-yl)methylene)hydrazinyl)−3-oxoprop-1-en-2-yl)benzamide (12)

A solution of the hydrazide **2** (3.95 g, 0.01 mol) and 5-chloro-3-methyl-1-phenylpyrazole-4-carbaldehyde (2.20 g, 0.01 mol) in absolute ethanol (20 mL) was refluxed for 3 h. The formed precipitate was filtered and recrystallized from ethanol to obtain white crystal, yield 75%, mp. 222–224 °C. IR (ν, cm^−1^): 3274, 3215 (NH), 1661 (C = O), 1636 (C = N). ^1^H NMR (DMSO-*d*_6_, *δ*, ppm): 11.59 (*br*.s, 1H, *NH*N, exchangeable), 9.97 (*br*.s, 1H, *NH*CO, exchangeable), 8.42 (s, 1H, CH = N), 8.00-7.44.00.44.00.44.00.44 (m, 15 H, Ar-H), 6.71 (s, 1H, CH=), 2.45, 2.14 (s, 6 H, 2CH_3_). GCMS (*m/z*, %): 597 (M^+^., 21), 569 (72), 476 (34), 431 (37), 379 (17), 255 (41), 236 (46), 219 (59), 193 (78), 101 (59), 94 (100), 84 (37). Anal.: calcd. for C_31_H_25_Cl_2_N_7_O_2_ (598.49): C: 62.21, H: 4.21, N: 16.38, found; C: 62.14, H: 4.13, N: 16.27.

### N-(1-(5-Chloro-3-methyl-1-phenyl-1H-pyrazol-4-yl)−3-(4-methyl-6-phenylpyrazolo[3,4-c]pyrazol-1(6H)-yl)−3-oxoprop-1-en-2-yl)benzamide (13)

A solution of the hydrazide **2** (3.95 g, 0.01 mol) and 5-chloro-3-methyl-1-phenylpyrazole-4-carbaldehyde (2.20 g, 0.01 mol) in acetic acid (20 mL) was refluxed for 4 h. The formed precipitate was filtered, washed several times with petroleum ether (60–80), and recrystallized from benzene to give orange crystals. Yield 60%, mp. 203–205 °C. IR (ν, cm^−1^): 3390 (NH), 1710, 1647 (C = O). ^1^H NMR (DMSO-*d*_6_, *δ*, ppm): 9.42 (*br*.s, 1H, NH, exchangeable), 8.57 (s, 1H, C3-H pyrazolo), 8.15–7.36 (m, 15 H, Ar-H), 7.05 (s, 1H, CH=), 2.71 (s, 3 H, CH_3_ pyrazolo), 2.38 (s, 3 H, CH_3_ pyrazole). GCMS (*m/z*, %): 561 (M^+^., 20), 539 (32), 506 (54), 469 (70), 359 (54), 323 (100), 296 (85), 235 (80), 199 (52), 140 (55), 111 (54), 83 (46). Anal.: calcd. for C_31_H_24_ClN_7_O_2_ (562.03): C: 66.25, H: 4.30, N: 17.45, found; C: 66.30, H: 4.37, N: 17.59.

### *Ethyl 2-(2-(2-(2-benzamido-3-(5-chloro-3-methyl-1-phenyl-1H-pyrazol-4-yl)acryloyl)-hydrazono)−4-oxo-3-phenylthiazolidin-5-ylidene)acetate (14)*

A solution of compound **9** (5.30 g, 0.01 mol) with diethyl acetylenedicarboxylate (1.7 mL, 0.01 mol) and freshly fused sodium acetate (0.82 g, 0.01 mol) in absolute ethanol (15 mL) was refluxed for 3 h. The reaction mixture was poured onto ice-cold water, and the forming precipitate was filtered and recrystallized from benzene to give yellow crystals. Yield 76%, mp. 118–120 °C. IR (ν, cm^−1^): 3254 (NH), 1736 (C = O ester), 1695, 1653 (C = O amide), 1622 (C = N). ^1^H NMR (DMSO-*d*_6_, *δ*, ppm): 11.41, 9.99 (*br*.s, 2 H, 2NH, exchangeable), 8.00-6.99 (m, 15 H, Ar-H), 6.92 (s, 1H, =CH), 6.87 (s,  H, COCH=), 4.20 (q, 2 H, *CH*_*2*_CH_3_, *J* = 6.7 *Hz*), 2.15 (s, 3 H, CH_3_), 1.20 (t, 3 H, *CH*_*3*_CH_2_, *J* = 6.8 *Hz*). GCMS (*m/z*, %): 656 (M^+^.+2, 71), 654 (M^+^., 25), 608 (56), 498 (29), 438 (79), 379 (50), 355 (44), 268 (100), 205 (46), 169 (29), 115 (74), 97 (35). Anal.: calcd. for C_33_H_27_ClN_6_O_5_S (655.13): C: 60.50, H: 4.15, N: 12.83, found; C: 60.59, H: 4.29, N: 12.89.

### *N-(2-(5-Chloro-3-methyl-1-phenyl-1H-pyrazol-4-yl)−1-(5-(phenylamino)−1*,*3*,*4-thiadiazol-2-yl)vinyl)benzamide (16)*

Refluxing derivative **9** (5.30 g, 0.01 mol) with ethyl chloroacetate (1.22 mL, 0.01 mol) and freshly fused sodium acetate (0.82 g, 0.01 mol) in ethanol (15 mL) for 3 h. The solid which deposited after pouring onto ice-cold water was filtrated, dried, and recrystallized from benzene to furnish pale-yellow crystals. Yield 73%, mp. 212–214 °C. IR (ν, cm^−1^): 3224 (NH), 1677 (C = O), 1640 (C = N). ^1^H NMR (DMSO-*d*_6_, *δ*, ppm): 10.54 (*br*.s, 1H, *NH*Ph, exchangeable), 10.29 (*br*.s, 1H, NHCO, exchangeable), 8.01–6.97 (m, 15 H, Ar-H), 6.87 (s, 1H, CH=), 2.19 (s, 3 H, CH_3_). GCMS (*m/z*, %): 513 (M^+^.+2, 39), 512 (M^+^., 62), 480 (61), 455 (58), 391 (55), 348 (60), 302 (100), 252 (79), 197 (55), 136 (45), 112 (46), 42 (55). Anal.: calcd. for C_27_H_21_ClN_6_OS (513.02): C: 63.21, H: 4.13, N: 16.38, found; C: 63.08, H: 4.06, N: 16.31.

### Cytotoxicity assay

#### Cell lines

The cell lines: colorectal carcinoma (HCT-116) and mammary gland breast cancer (MCF-7) were obtained from ATCC *via* Holding company for biological products and vaccines (VACSERA), Cairo, Egypt.

#### Chemical reagents

The reagents RPMI-1640 medium, MTT, and DMSO (sigma co., St. Louis, USA), Fetal Bovine serum (GIBCO, UK). Doxorubicin was used as a standard anticancer drug for comparison.

### MTT assay

The cell lines mentioned above were used to determine the inhibitory effects of compounds on cell growth using the MTT assay. This colorimetric assay is based on the conversion of the yellow tetrazolium bromide (MTT) into a purple formazan derivative by mitochondrial succinate dehydrogenase in viable cells. Cell lines were cultured in RPMI-1640 medium with 10% fetal bovine serum. Antibiotics added to the medium included 100 units/ml penicillin and 100 mg/ml streptomycin. Cultures were maintained at 37 °C in a 5% CO_2_ incubator in a 96-well plate at a density of 1.0 × 10^4^ cells/well for 24 h. After incubation, the cells were treated with different concentrations of compounds and incubated for 24 h. After 24 h of drug treatment, 20 ml of MTT solution at 5 mg/ml was added and incubated for 4 h. Dimethyl sulfoxide (DMSO) in the volume of 100 ml was added into each well to dissolve the purple formazan formed. The colorimetric assay was measured and recorded at an absorbance of 570 nm using a plate reader (EXL 800, USA). The relative cell viability in percentage was calculated as (A_570_ of treated samples/A_570_ of untreated samples) x 100.

#### Molecular Docking

The crystal structure (PDB ID: 2A4L) of the human CDK2 complex was taken from the protein from the RSCB protein data bank (http://www.rcsb.org/pdb). The missing residues were added using a graphical user interface tool of molecular modeling, and a ligand interaction map was generated using the web version of pose view. The docking calculations were performed using AutoDock Vina. Initially, the protein structures were protonated, and the hydrogen atoms were hidden. Then, the energy was minimized, and the binding pockets of the protein were well-defined. The 2D structures of the prepared compounds were sketched using ChemBioDraw Ultra 14.0 and saved as MOL format. Then, the saved files were opened, and 3D structures were protonated. Next, energy minimization was applied. Before docking the synthesized ligands, validation of the docking protocol was carried out by running the simulation only using the co-crystallized ligands and low RMSD between docked and crystal conformations. The molecular docking of the synthesized compounds and the co-crystallized ligand was performed using a default protocol; Placement: Triangle Matcher, Rescoring 1: London dG, Refinement: Force field. For each selected compound, twenty independent docking runs were generated using genetic algorithm protocol.

### Statistical analysis

The bioassay was repeated in triplicate and the results were obtained as means ± standard deviation (SD, *n* = 3) using SPSS 13.0 program (SPSS Inc., Chicago, IL). Differences between groups were considered statistically significant at *ρ* values < 0.05.

## Supplementary Information

Below is the link to the electronic supplementary material.

Supplementary Material 1

## Data Availability

All data generated or analyzed during this study are included in this published article and its supplementary information files.
