# Supplementary material for: Synthesis, antiproliferative screening, and molecular docking of some heterocycles derived from N-(1-(5-chloro-3-methyl-1-phenyl-1H-pyrazol-4-yl)-3-hydrazineyl-3-oxoprop-1-en-2-yl)benzamide
Source: Sci Rep. 2025 Nov 24;15:41821. doi: 10.1038/s41598-025-27006-9 (PMC12647661; doi:10.1038/s41598-025-27006-9)
Supplement: Supplementary file 1 — Supplementary Material 1 [file 41598_2025_27006_MOESM1_ESM.pdf]

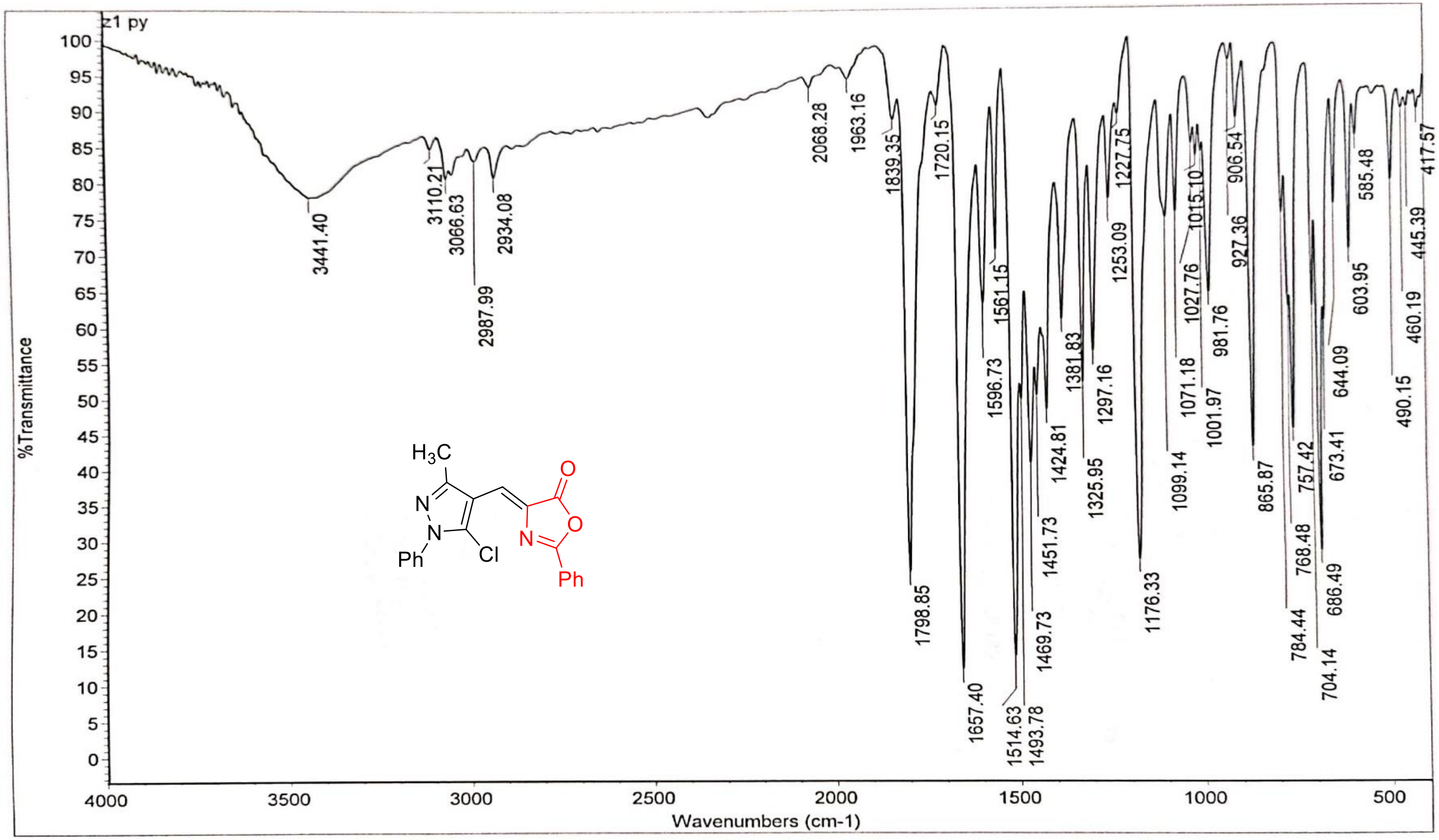

Fig. S1: IR spectrum of (1)

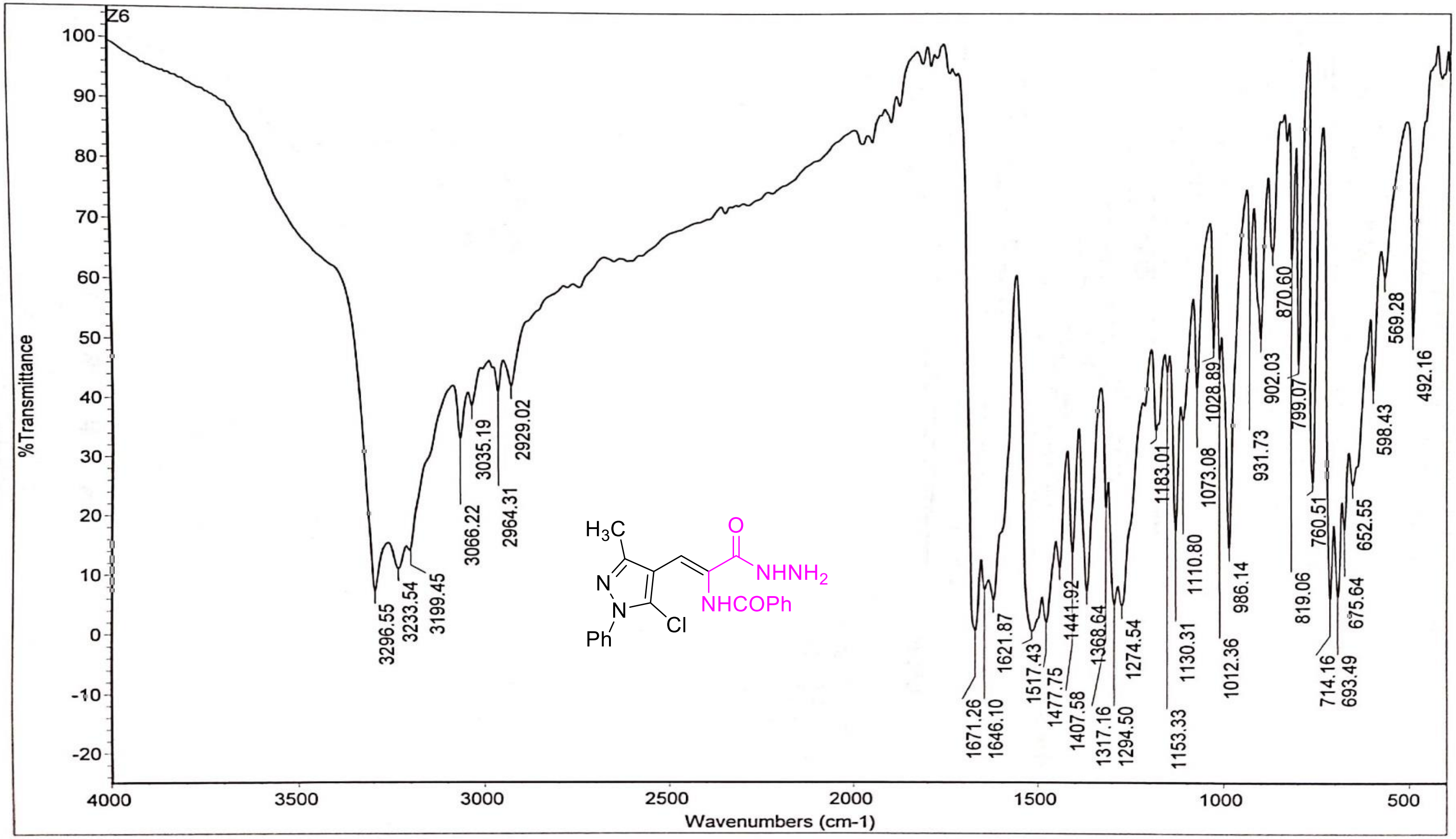

Fig. S2: IR spectrum of (2)

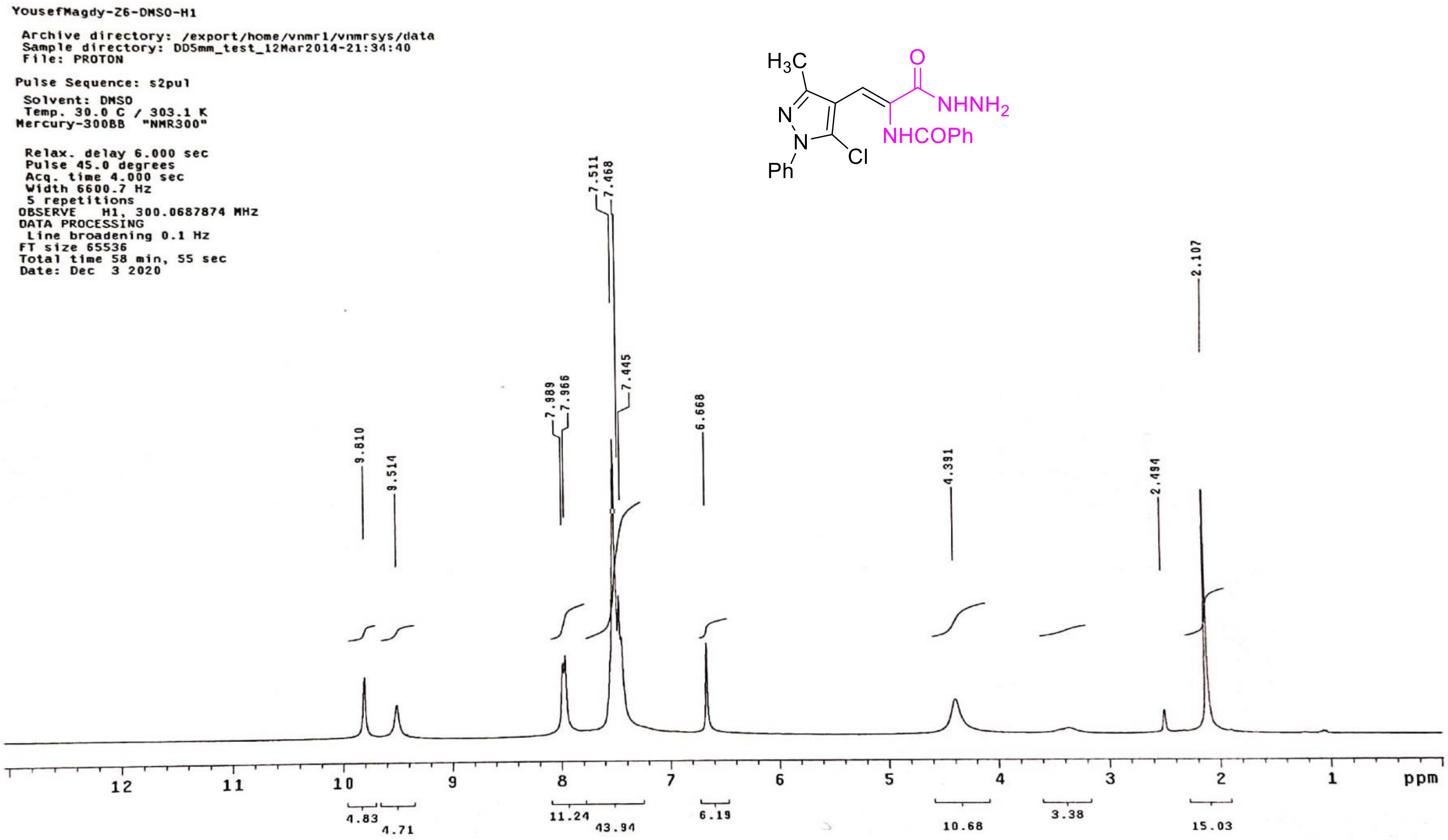

Fig. S3: ^{1}H NMR spectrum of (2)

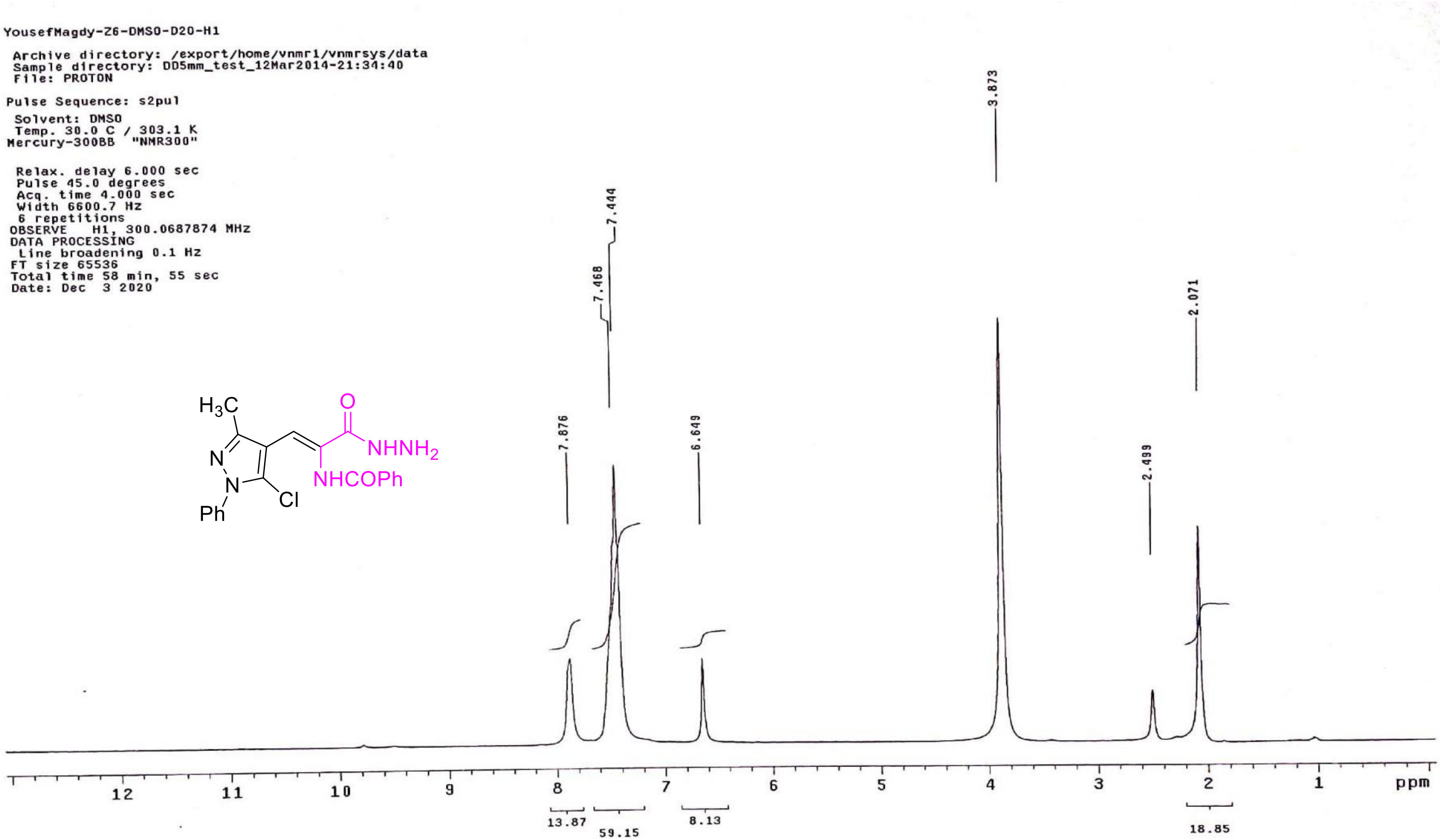

Fig. S4: ^{1}H NMR-D_2O spectrum of (2)

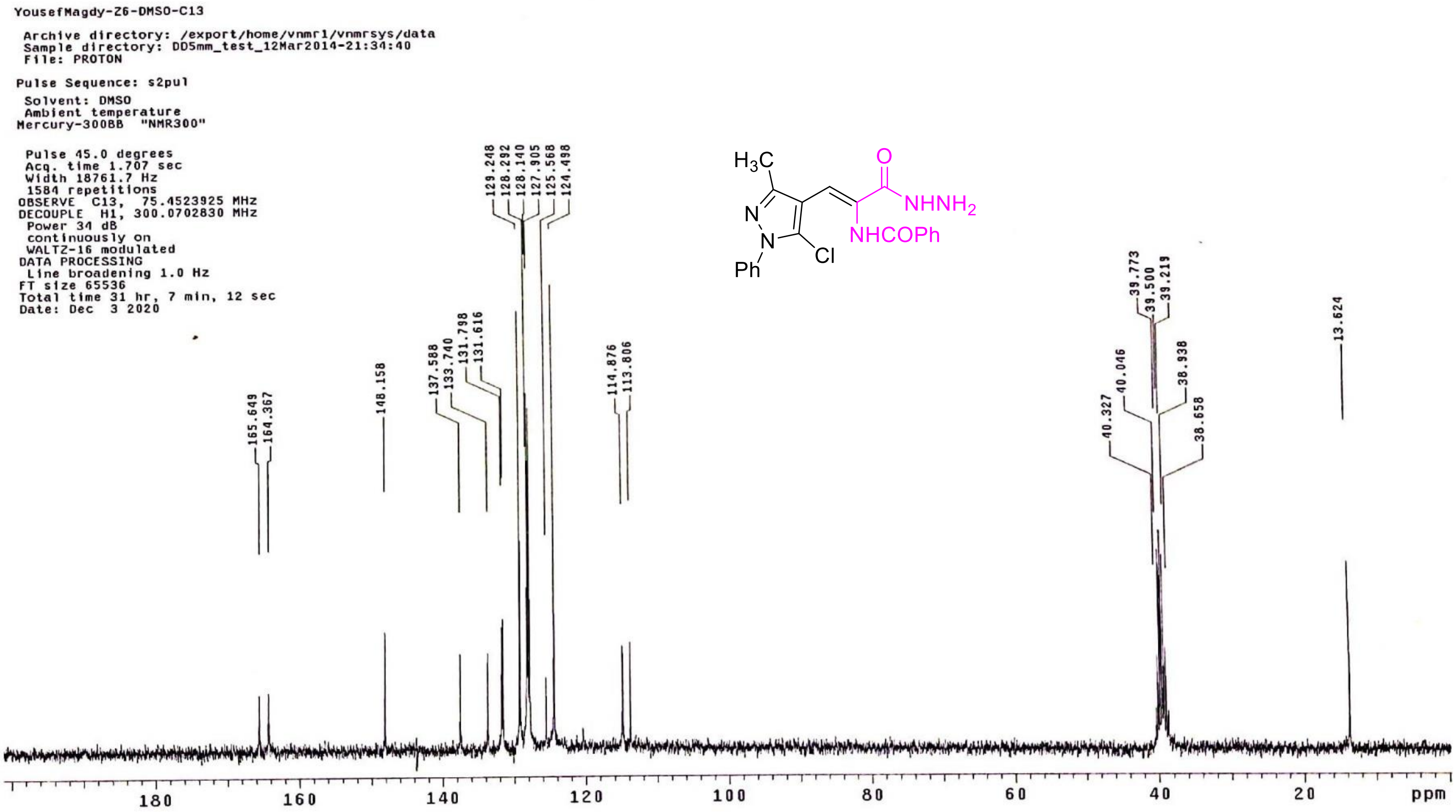

Fig. S5: ^{13}CNMR spectrum of (2)

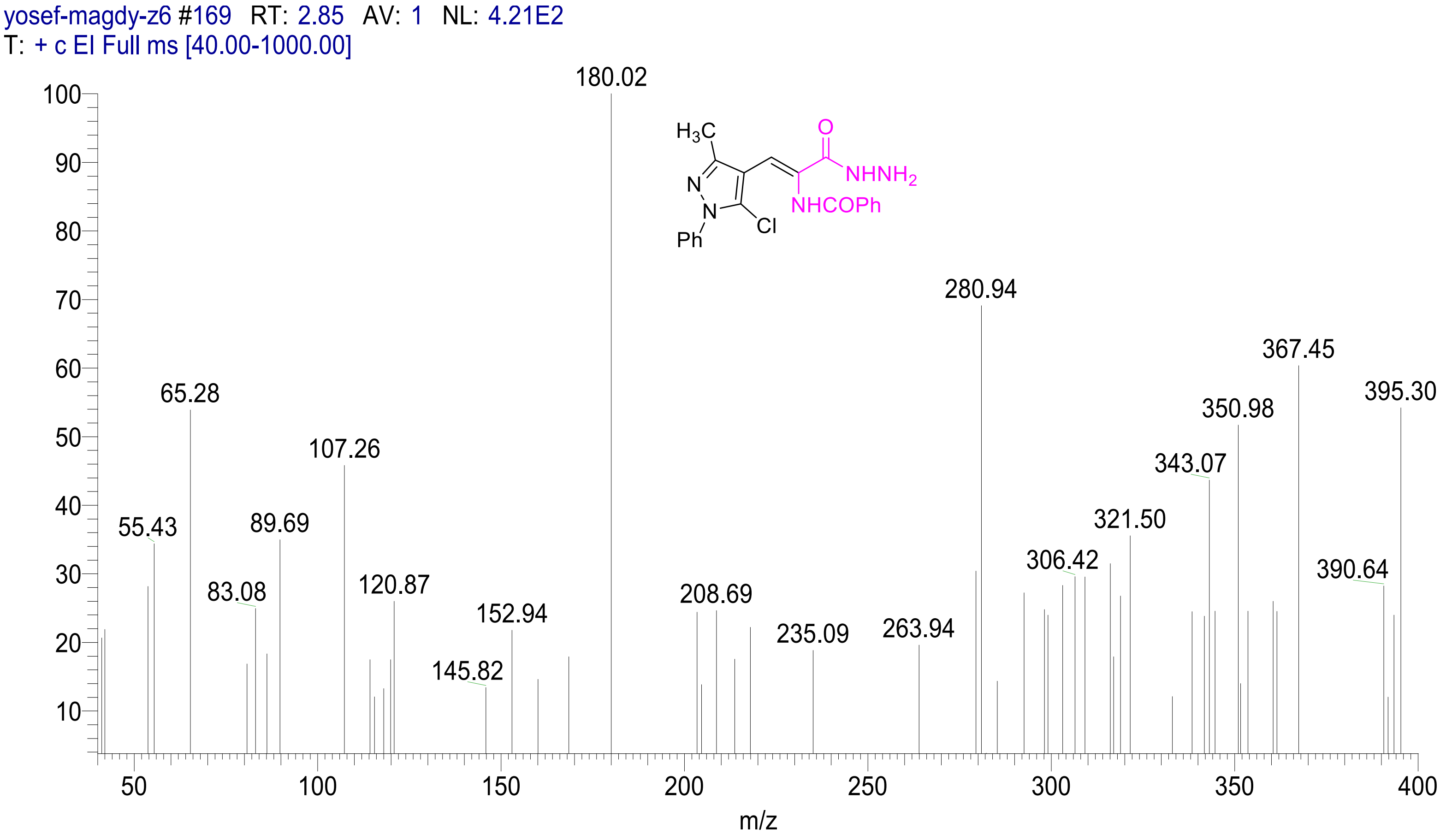

Fig. S6: Mass spectrum of (2)

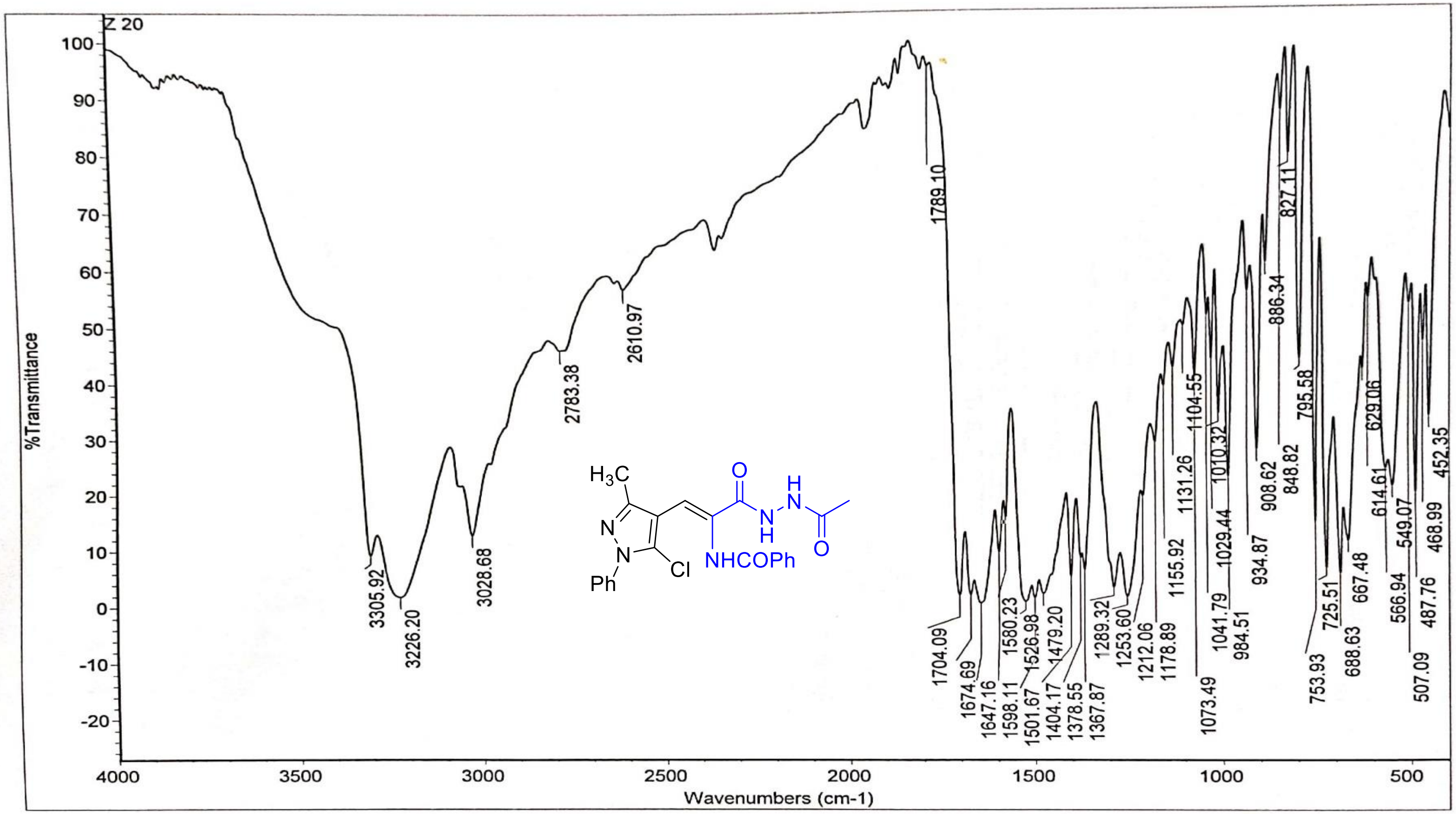

Fig. S7: IR spectrum of (3)

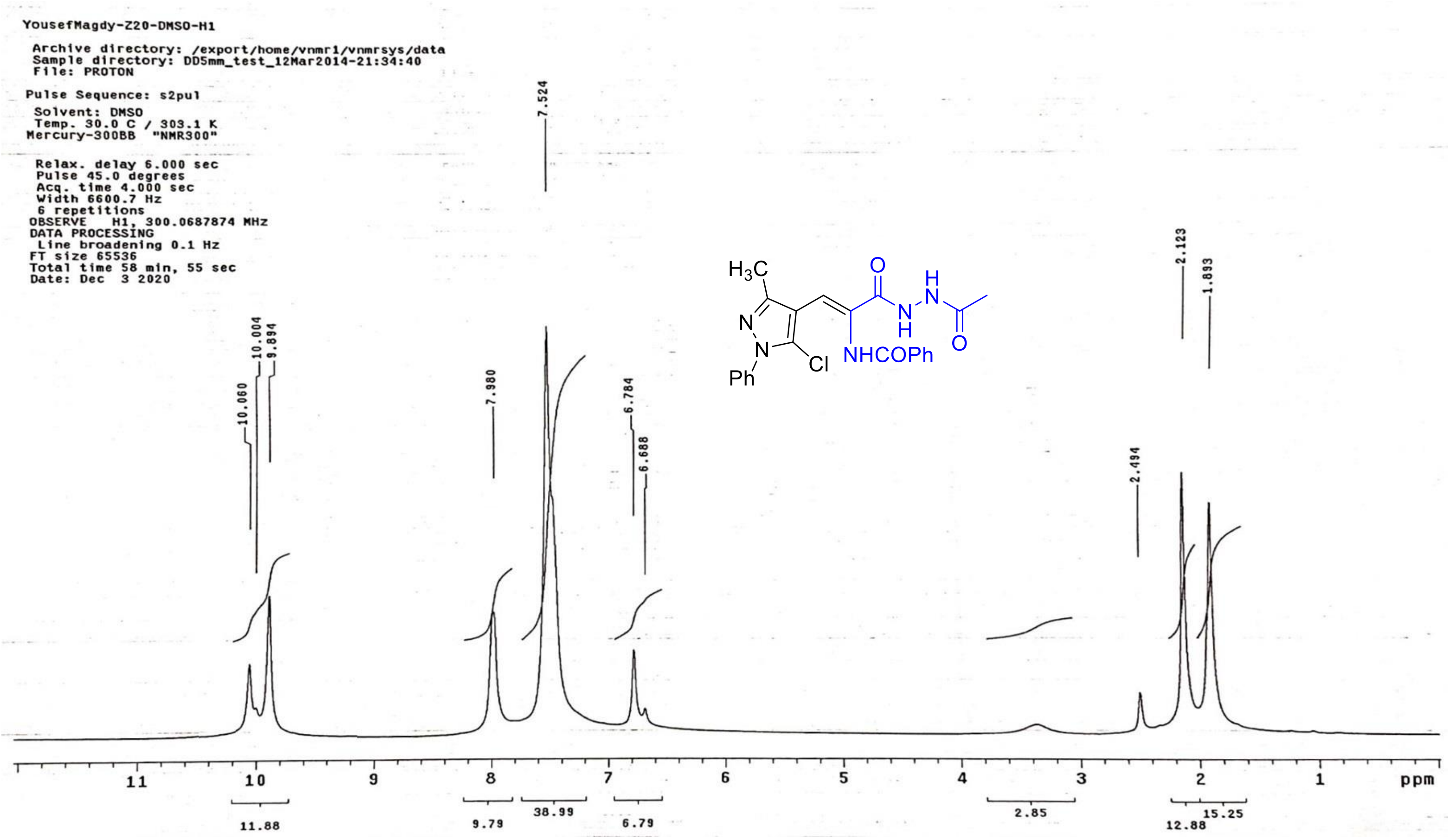

Fig. S8: ^{1}H NMR spectrum of (3)

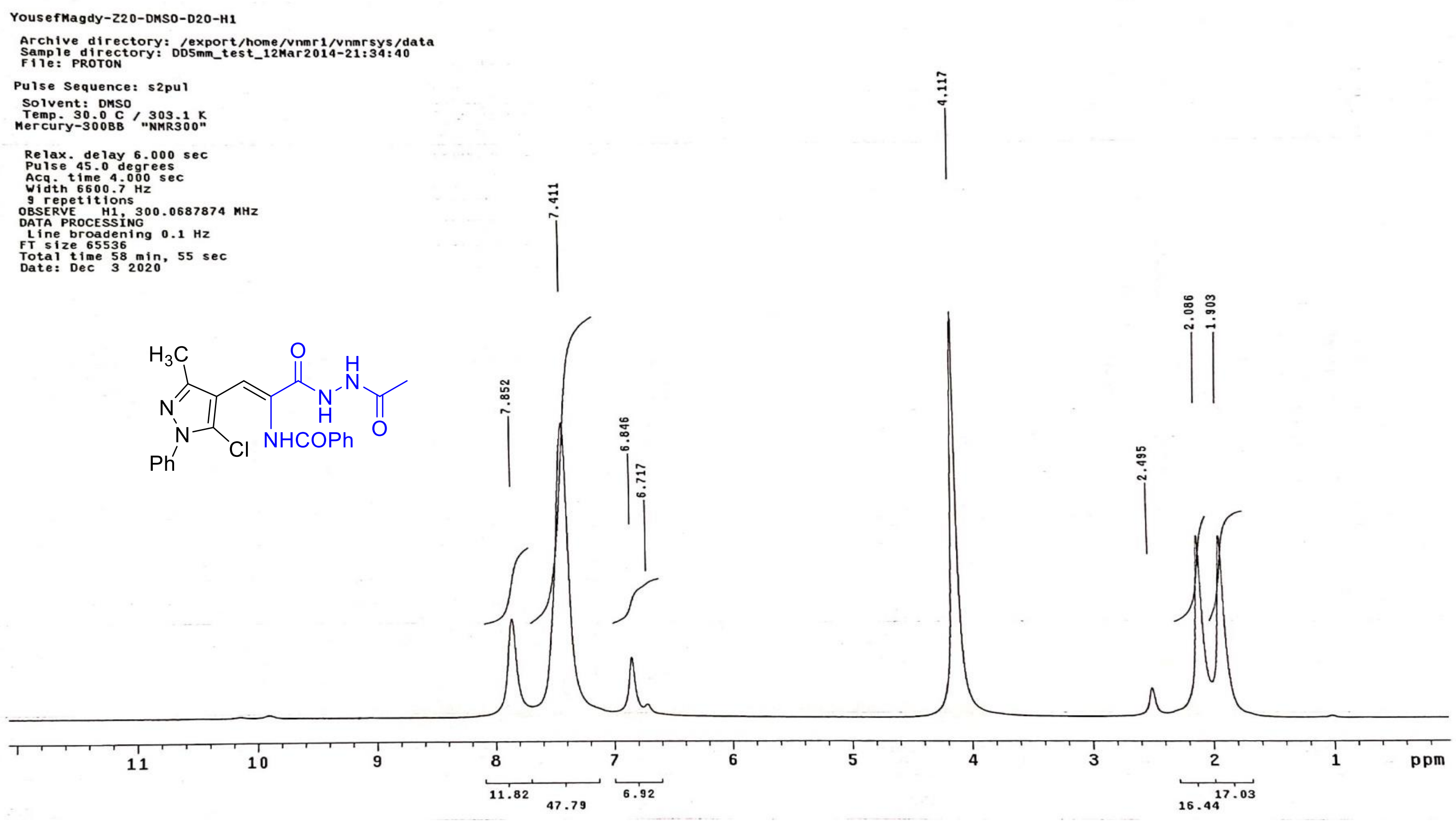

Fig. S9: ^{1}H NMR-D_2O spectrum of (3)

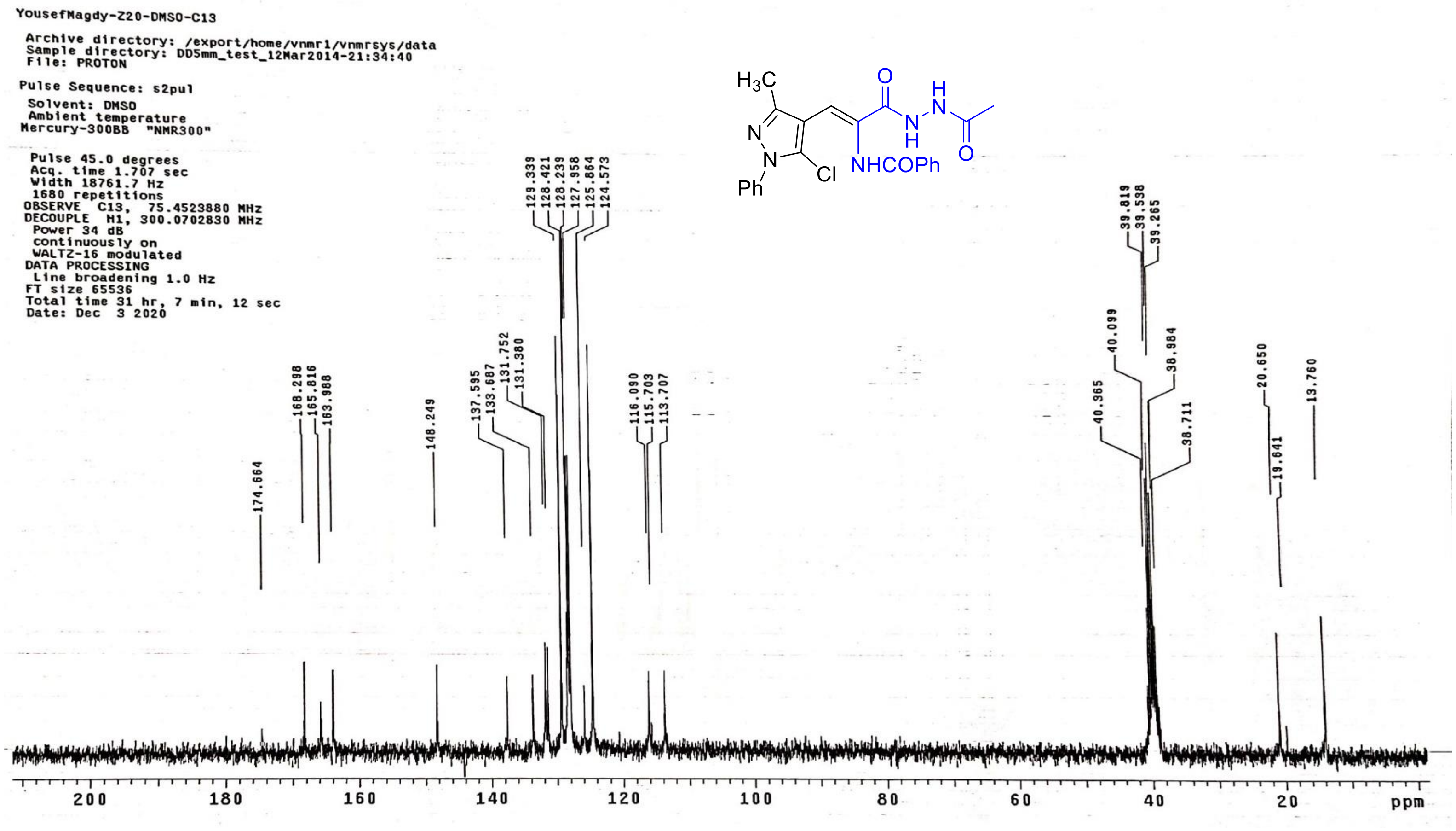

Fig. S10: ^{13}CNMR spectrum of (3)

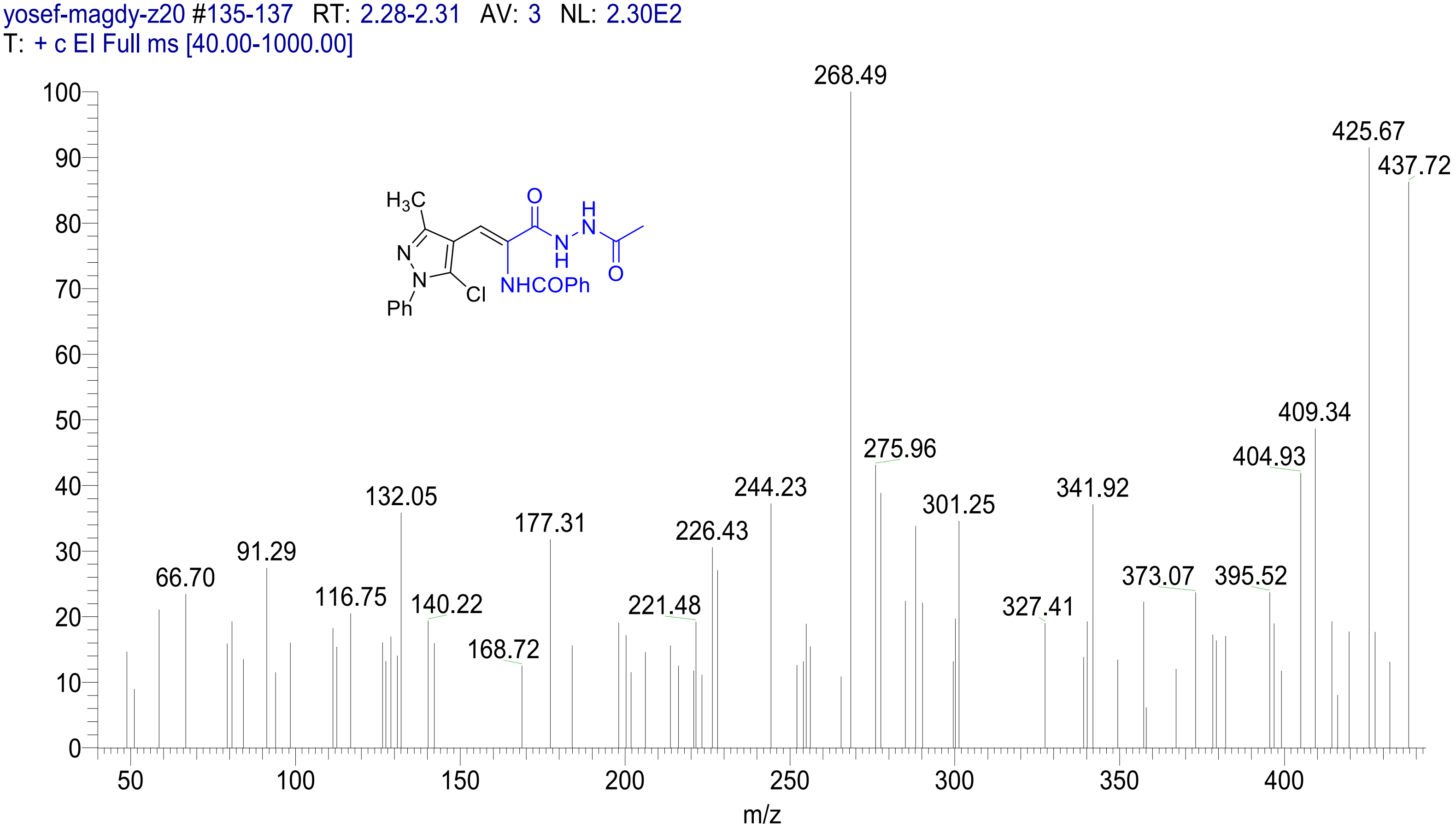

Fig. S11: Mass spectrum of (3)

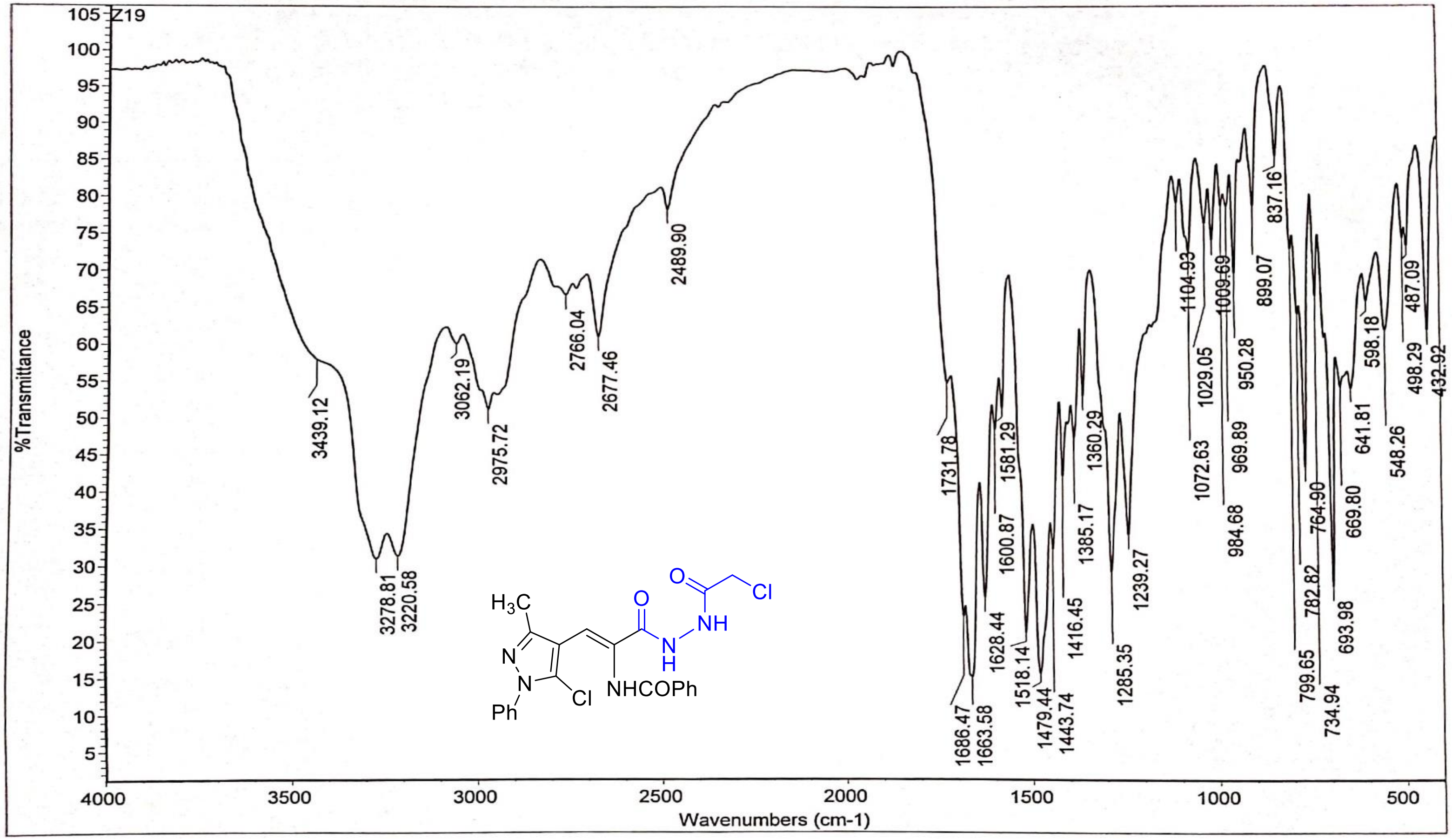

Fig. S12: IR spectrum of (4)

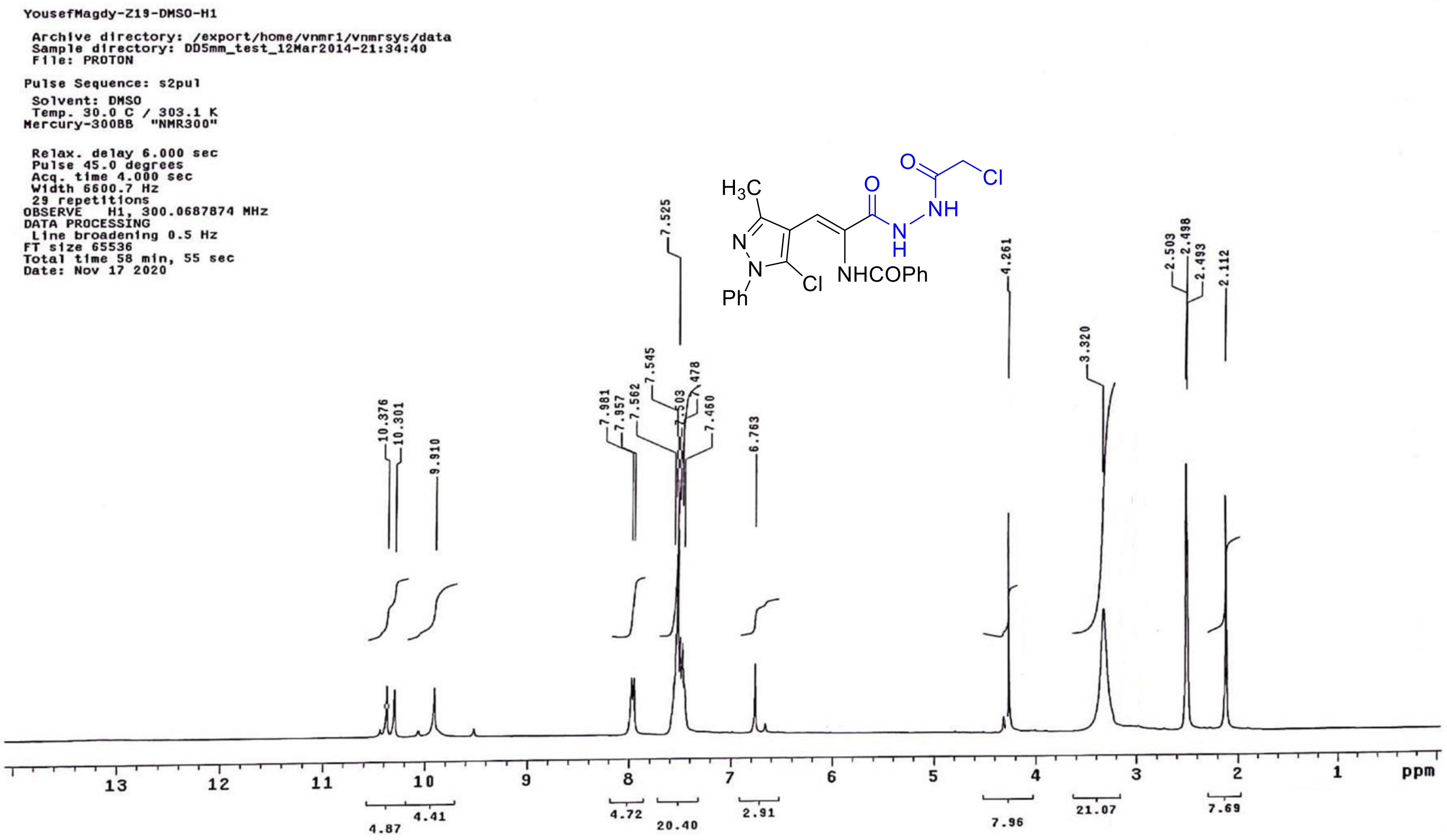

Fig. S13: ^{1}H NMR spectrum of (4)

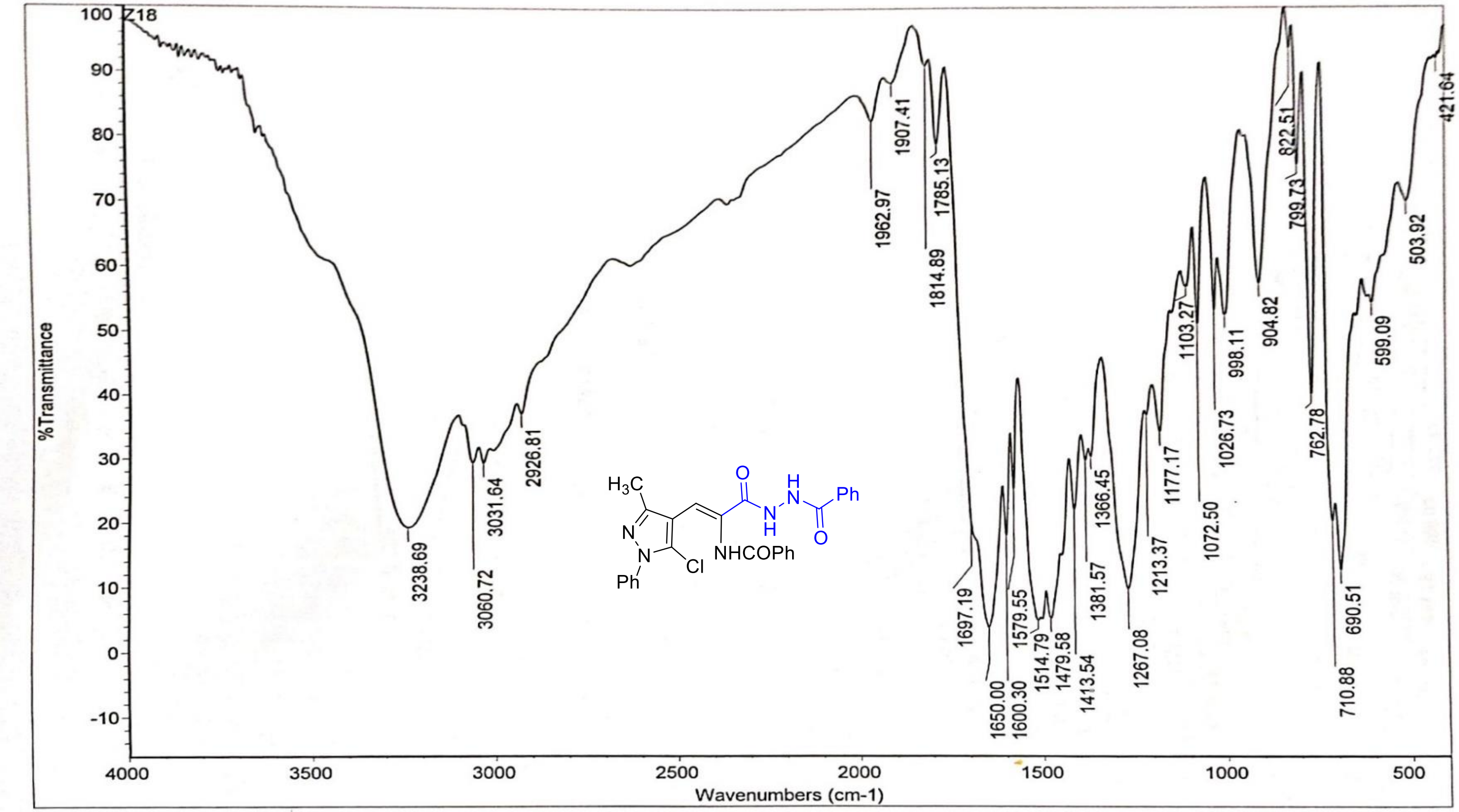

Fig. S14: IR spectrum of (5)

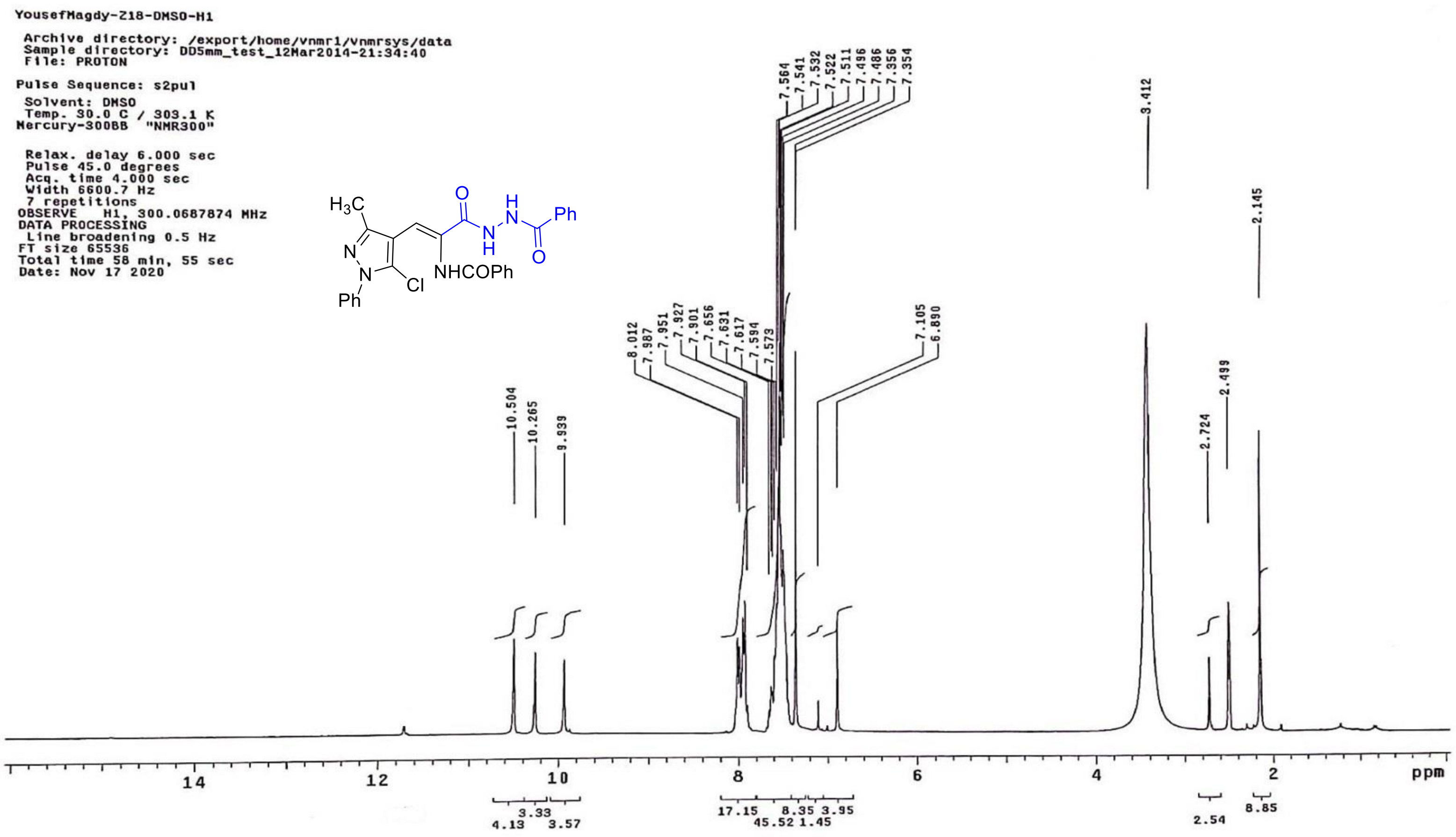

Fig. S15: ^{1}H NMR spectrum of (5)

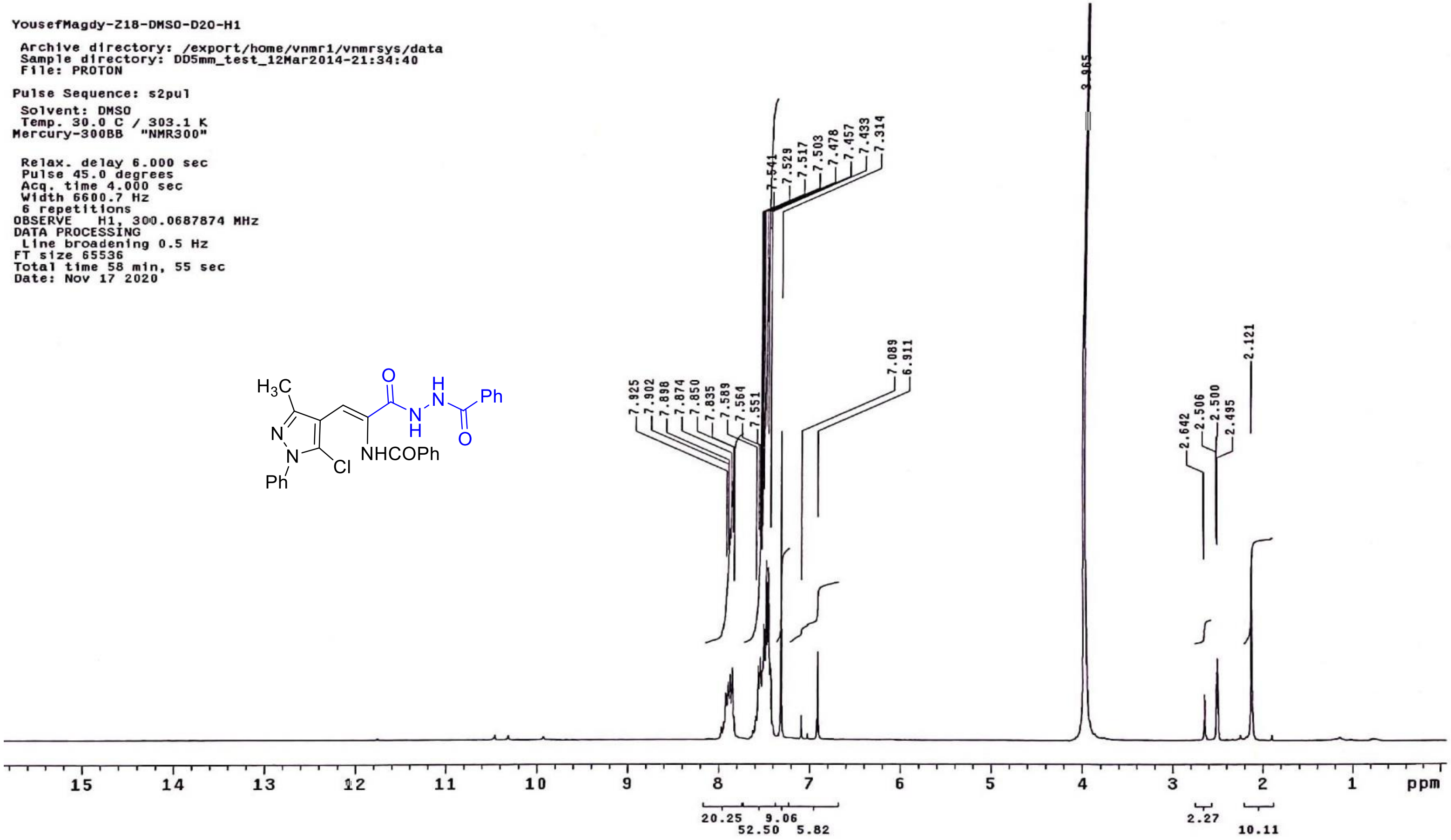

Fig. S16: ^{1}H NMR-D_2O spectrum of (5)

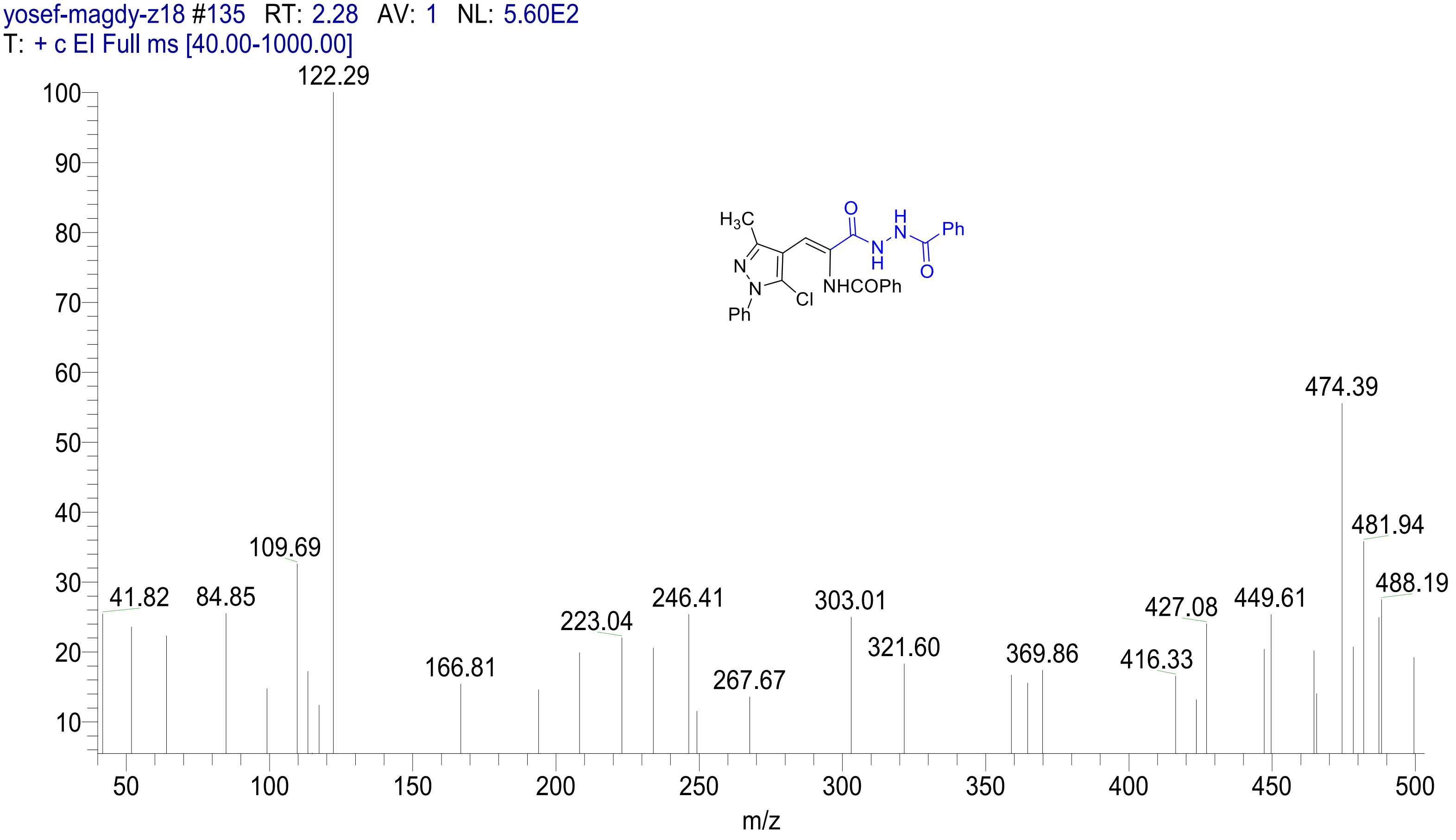

Fig. S17: Mass spectrum of (5)

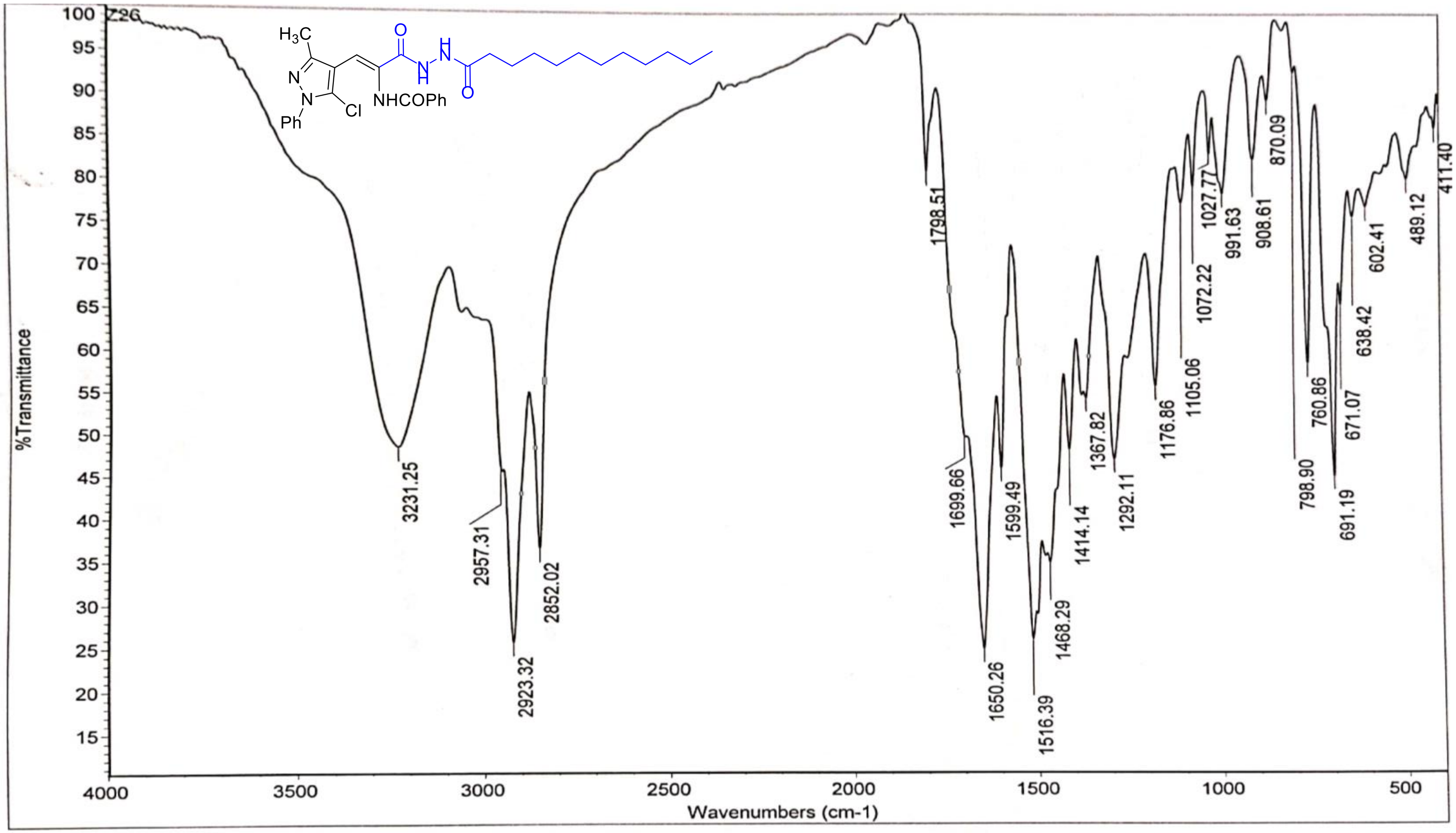

Fig. S18: IR spectrum of (6)

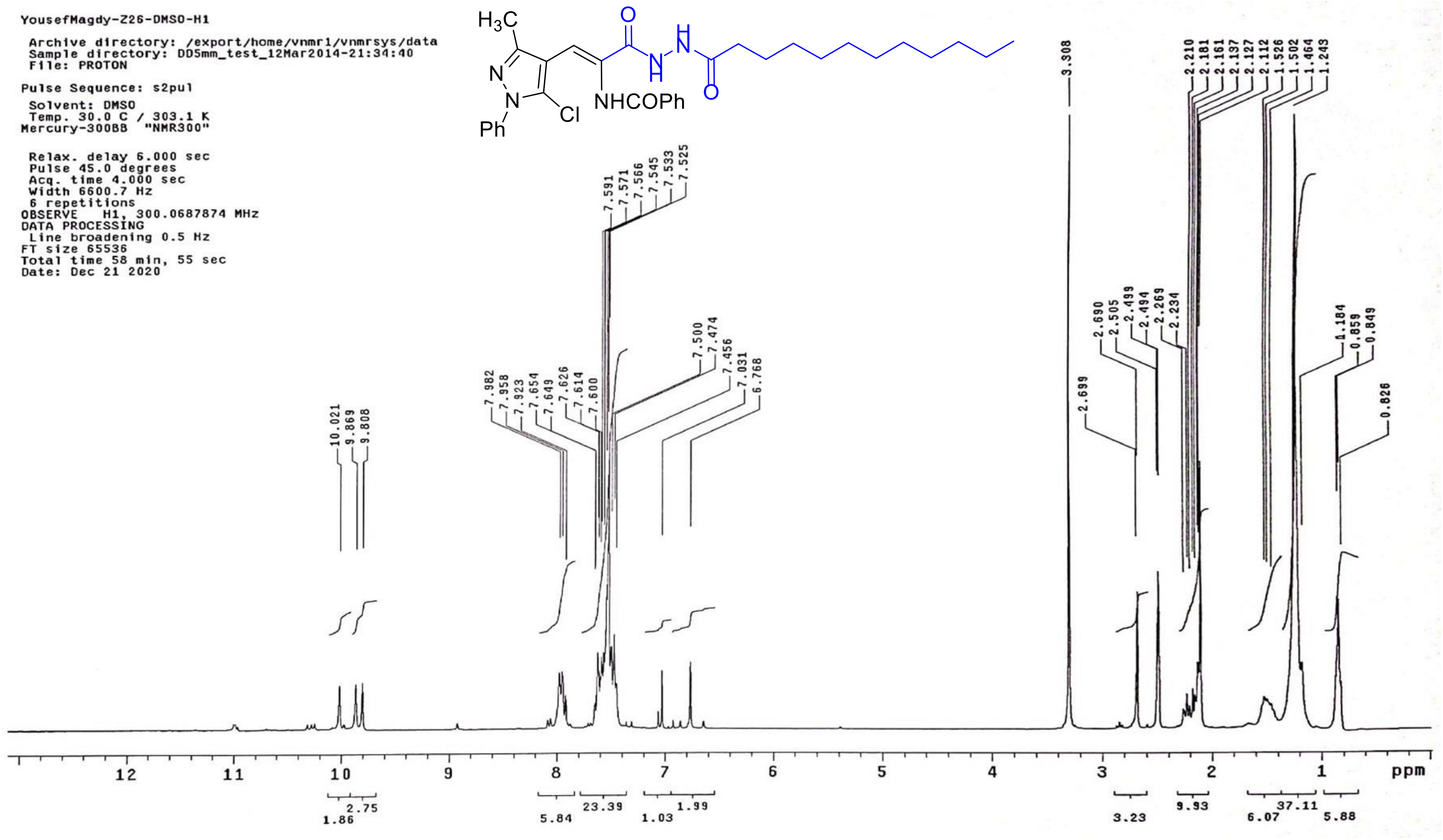

Fig. S19: ^{1}H NMR spectrum of (6)

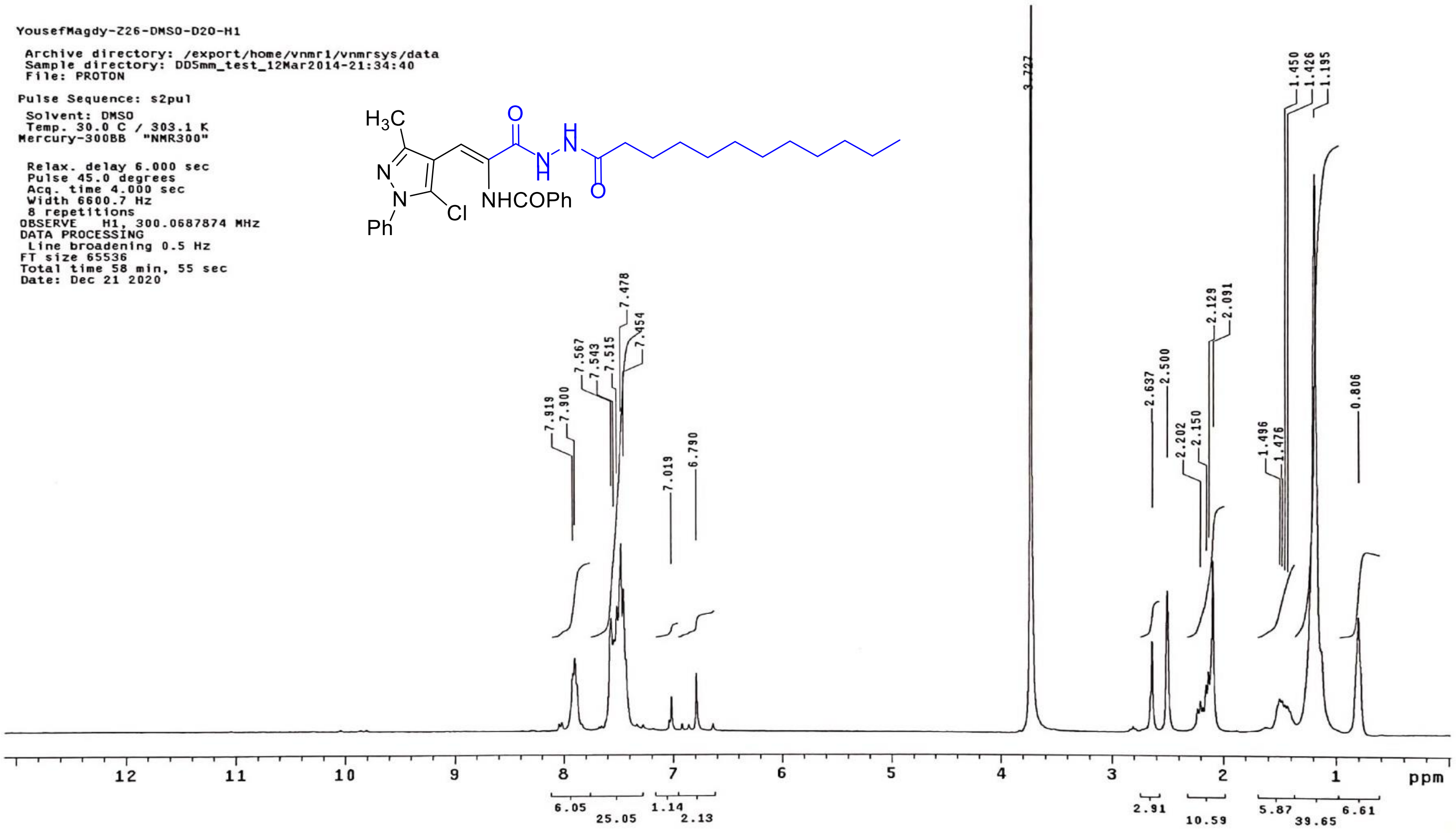

Fig. S20: ¹H NMR-D_2O spectrum of (6)

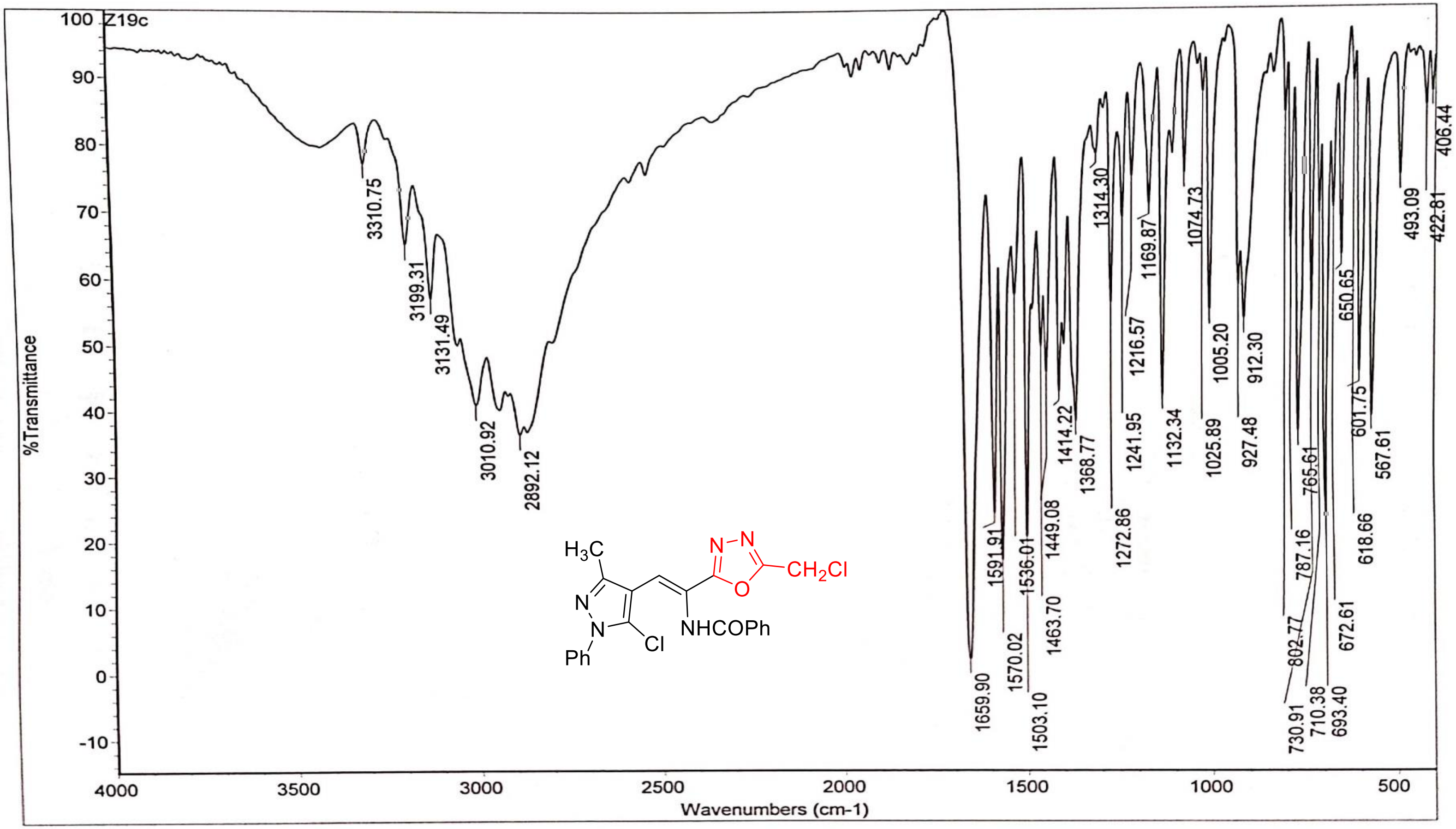

Fig. S21: IR spectrum of (7)

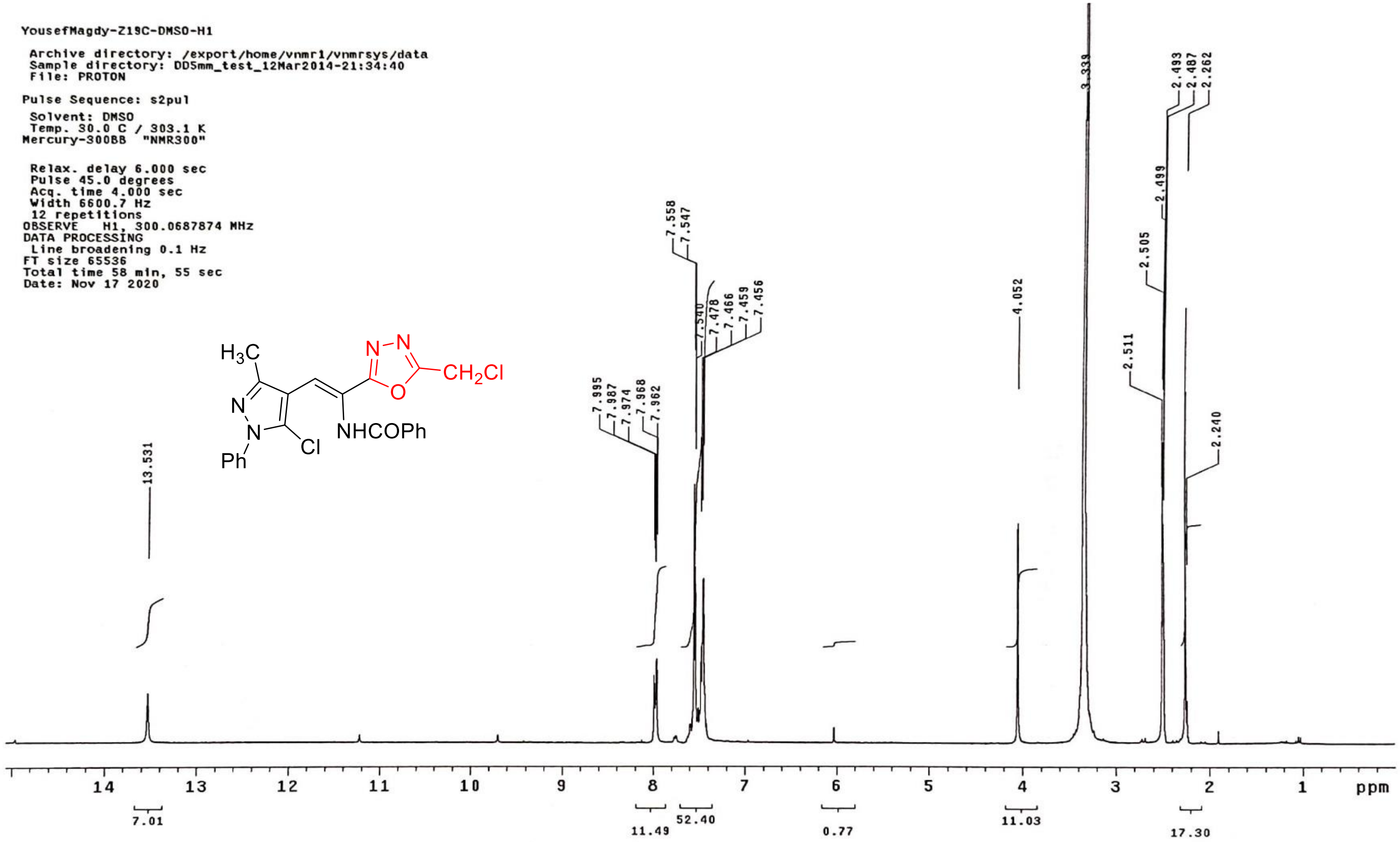

Fig. S22: ^{1}H NMR spectrum of (7)

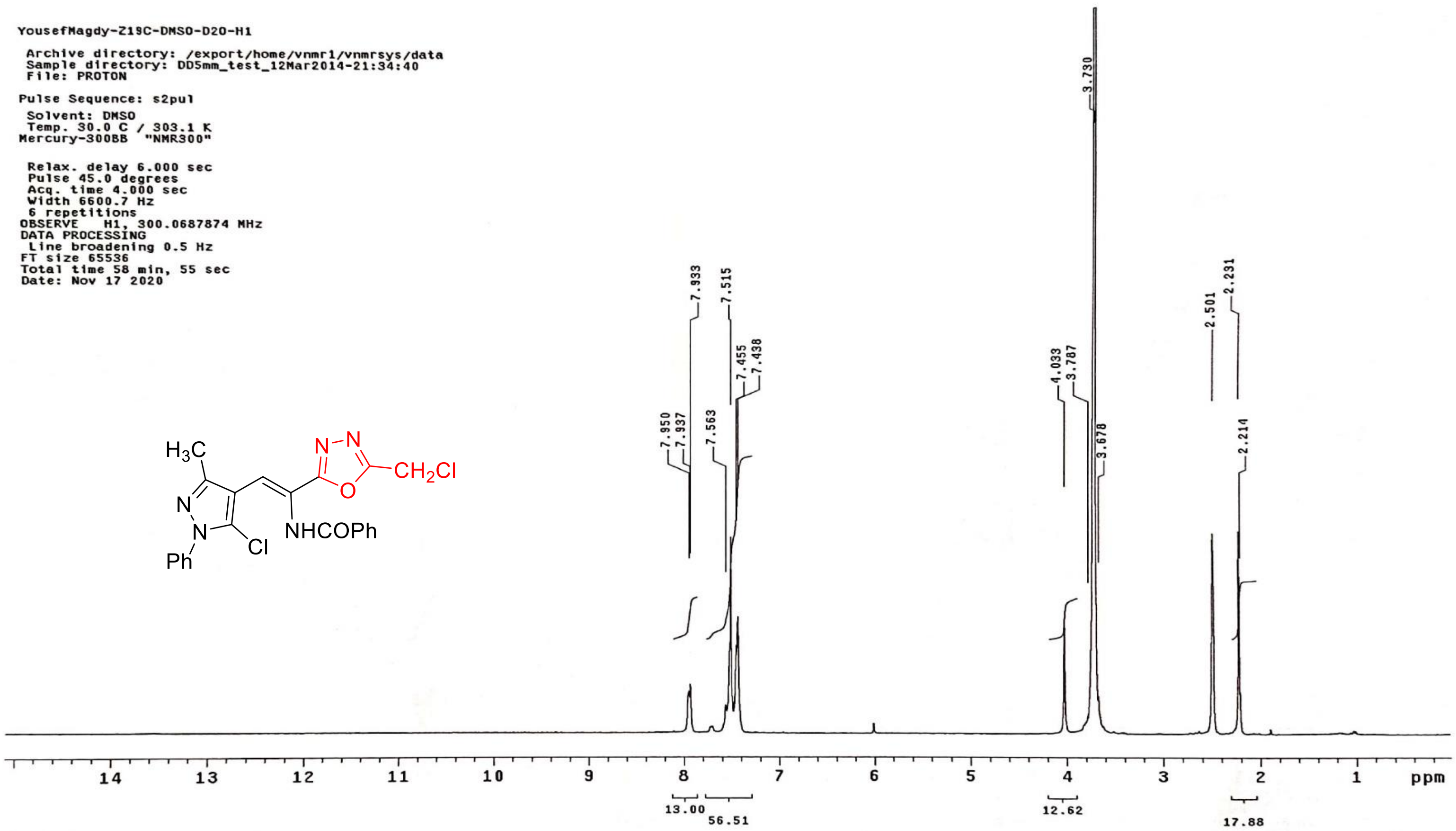

Fig. S23: ^{1}H NMR-D_2O spectrum of (7)

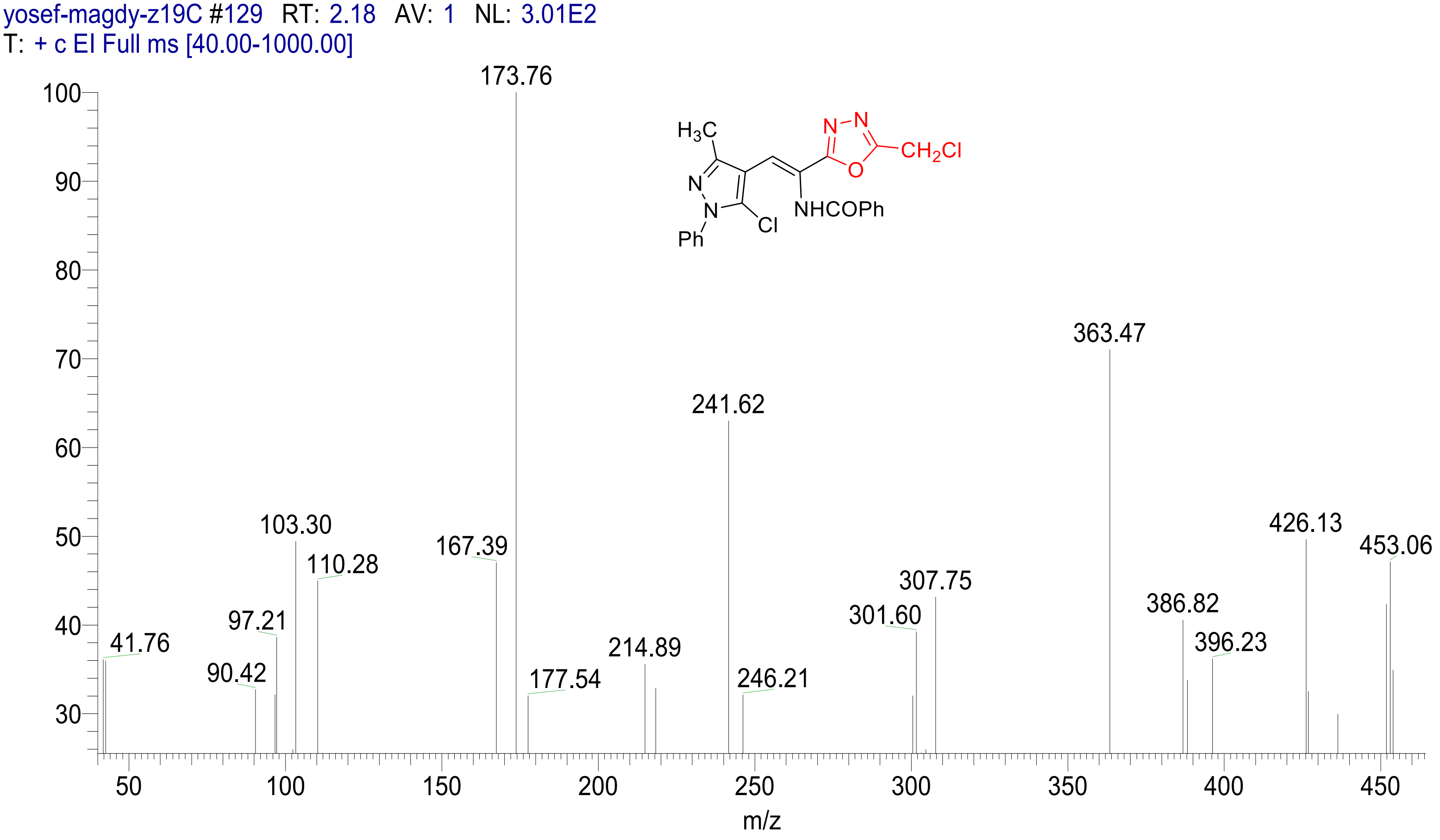

Fig. S24: Mass spectrum of (7)

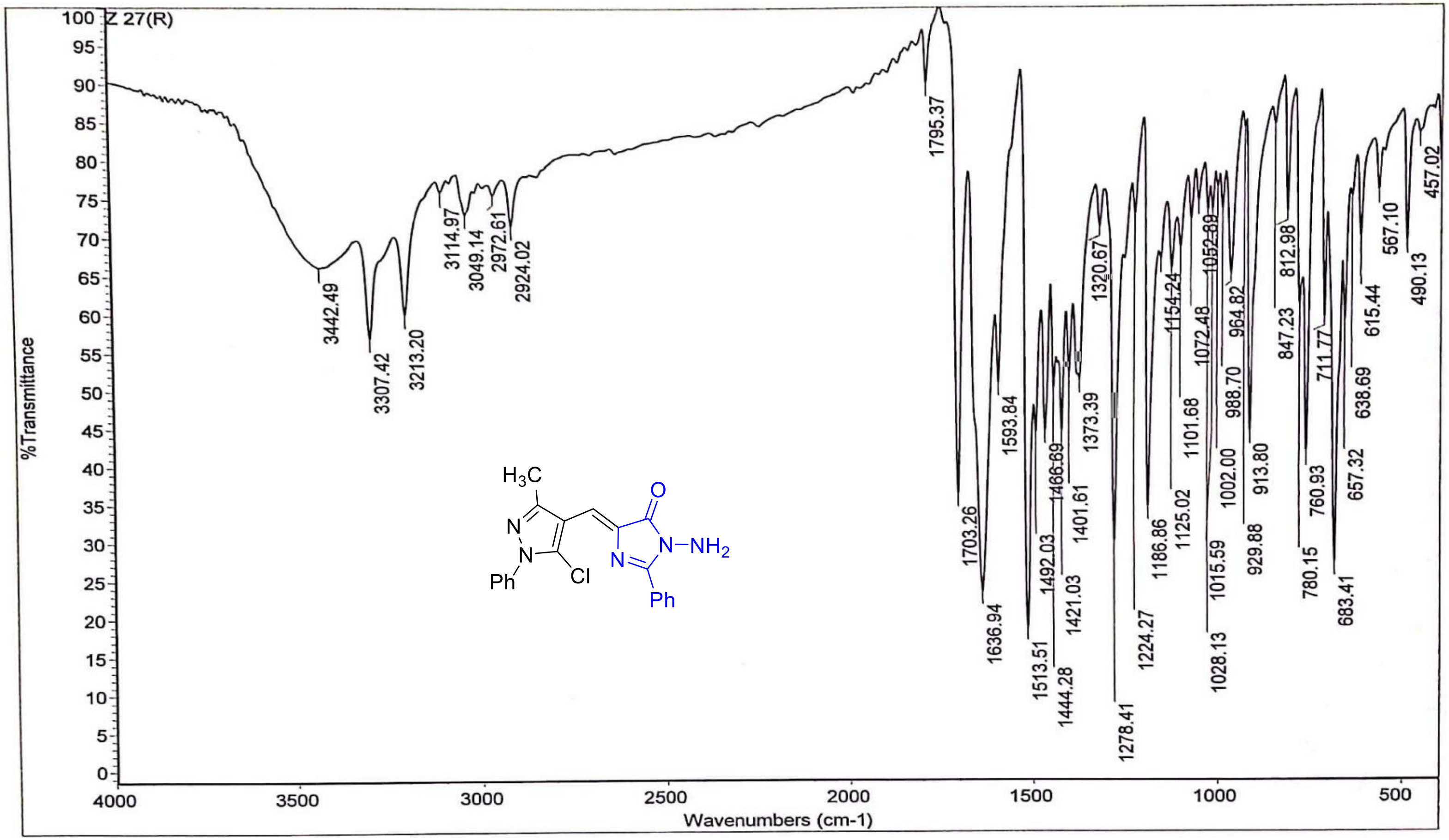

Fig. S25: IR spectrum of (8)

Figures

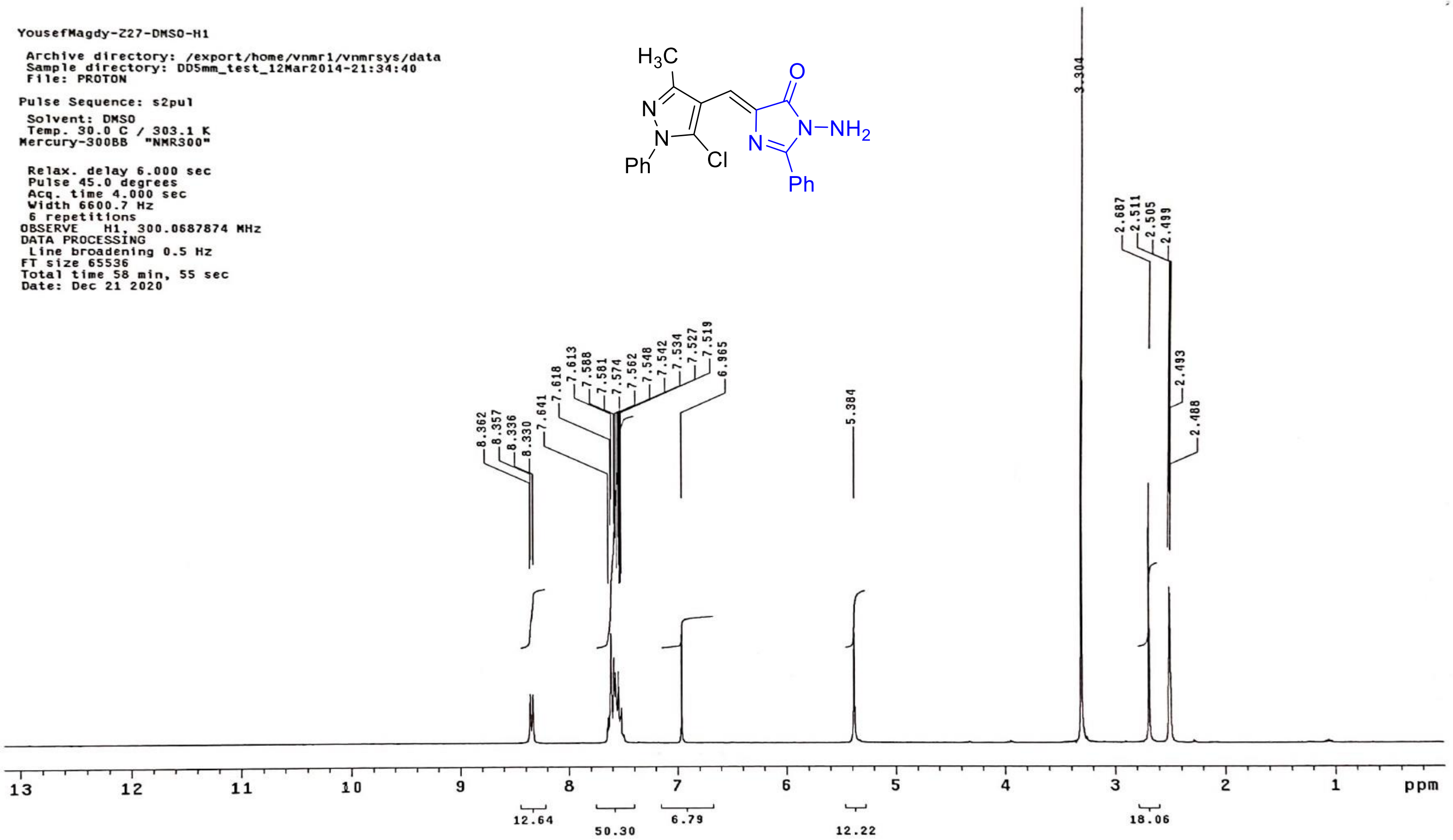

Fig. S26: ^{1}H NMR spectrum of (8)

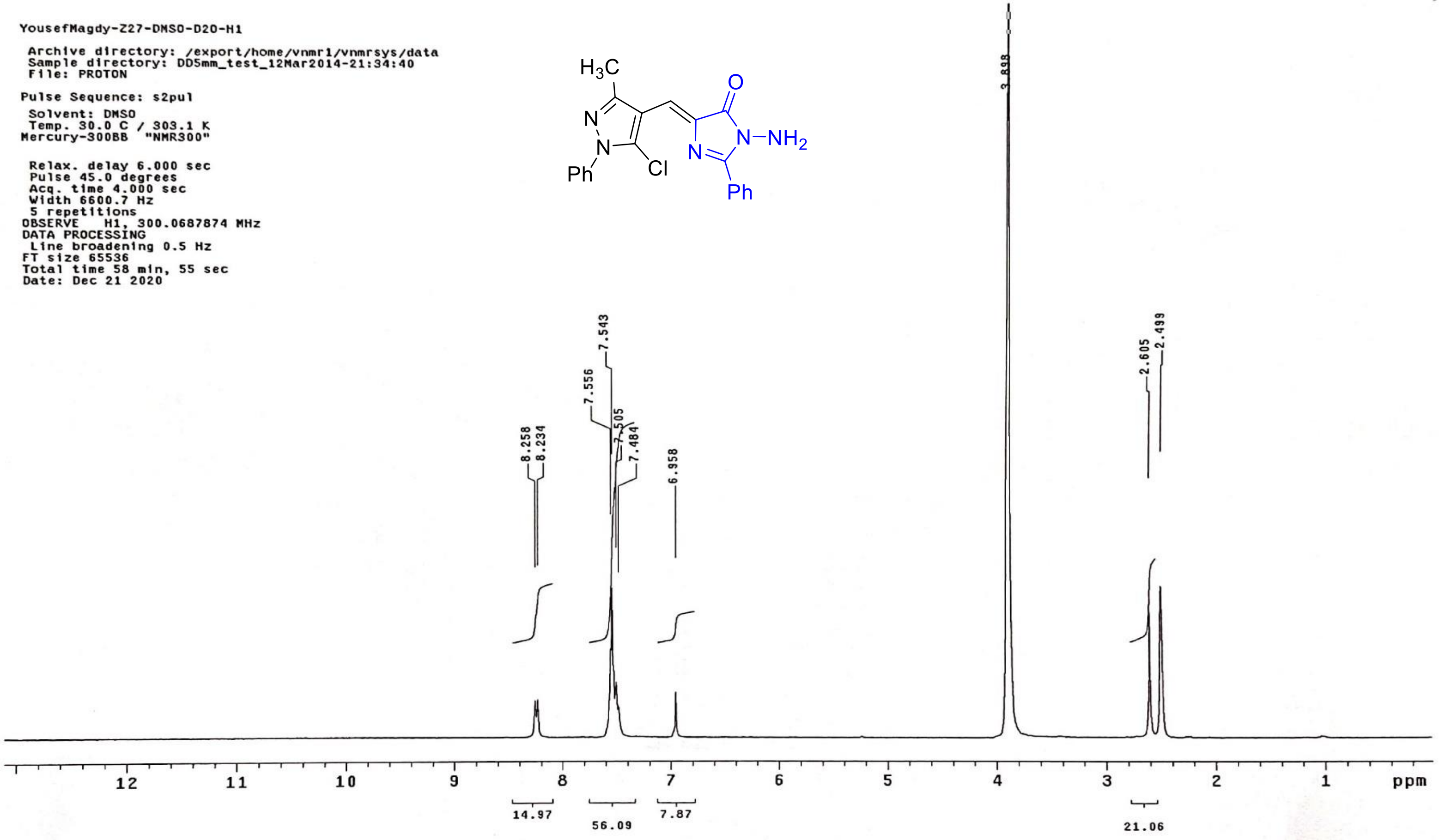

Fig. S27: ^{1}H NMR-D_2O spectrum of (8)

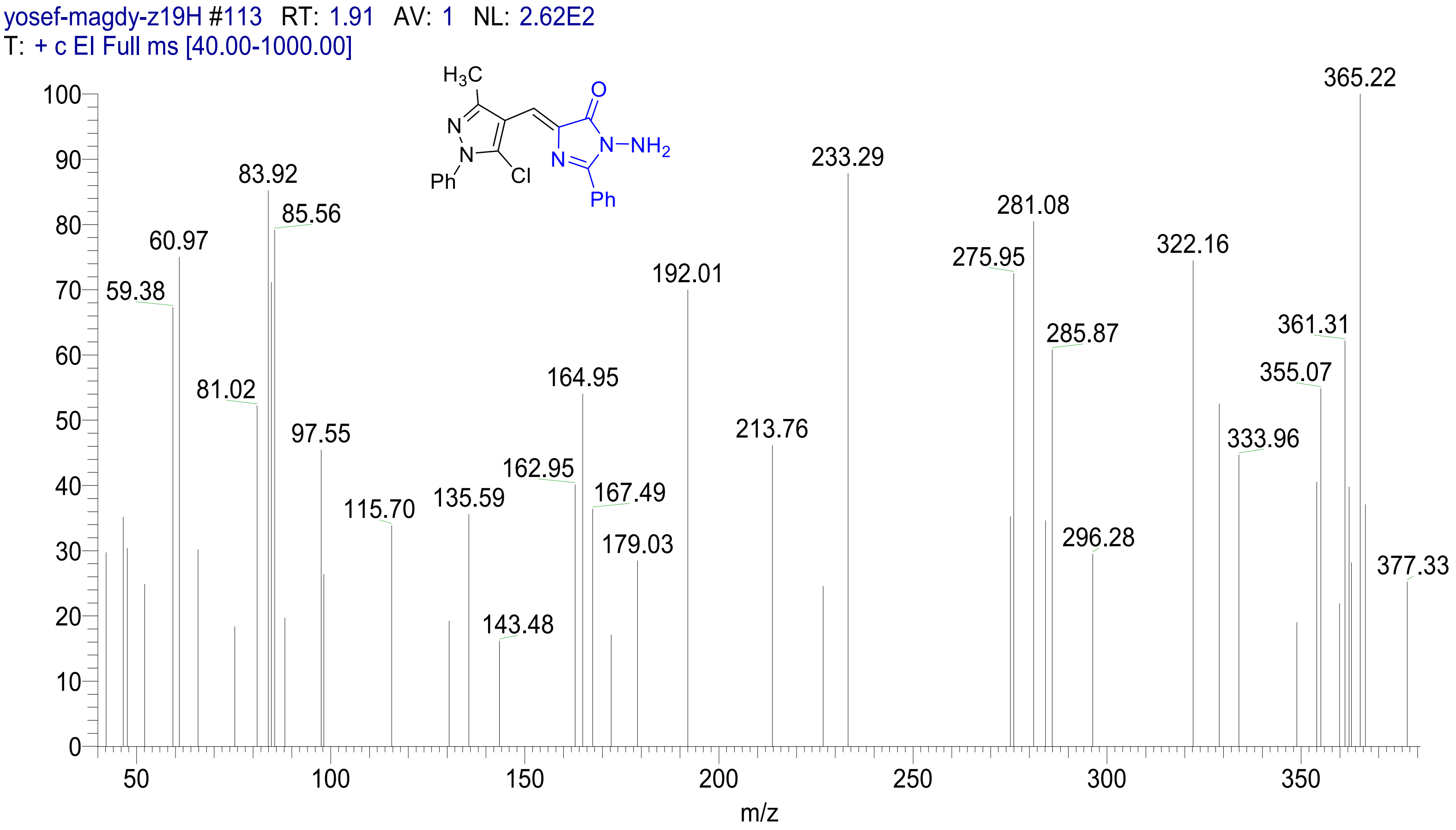

Fig. S28: Mass spectrum of (8)

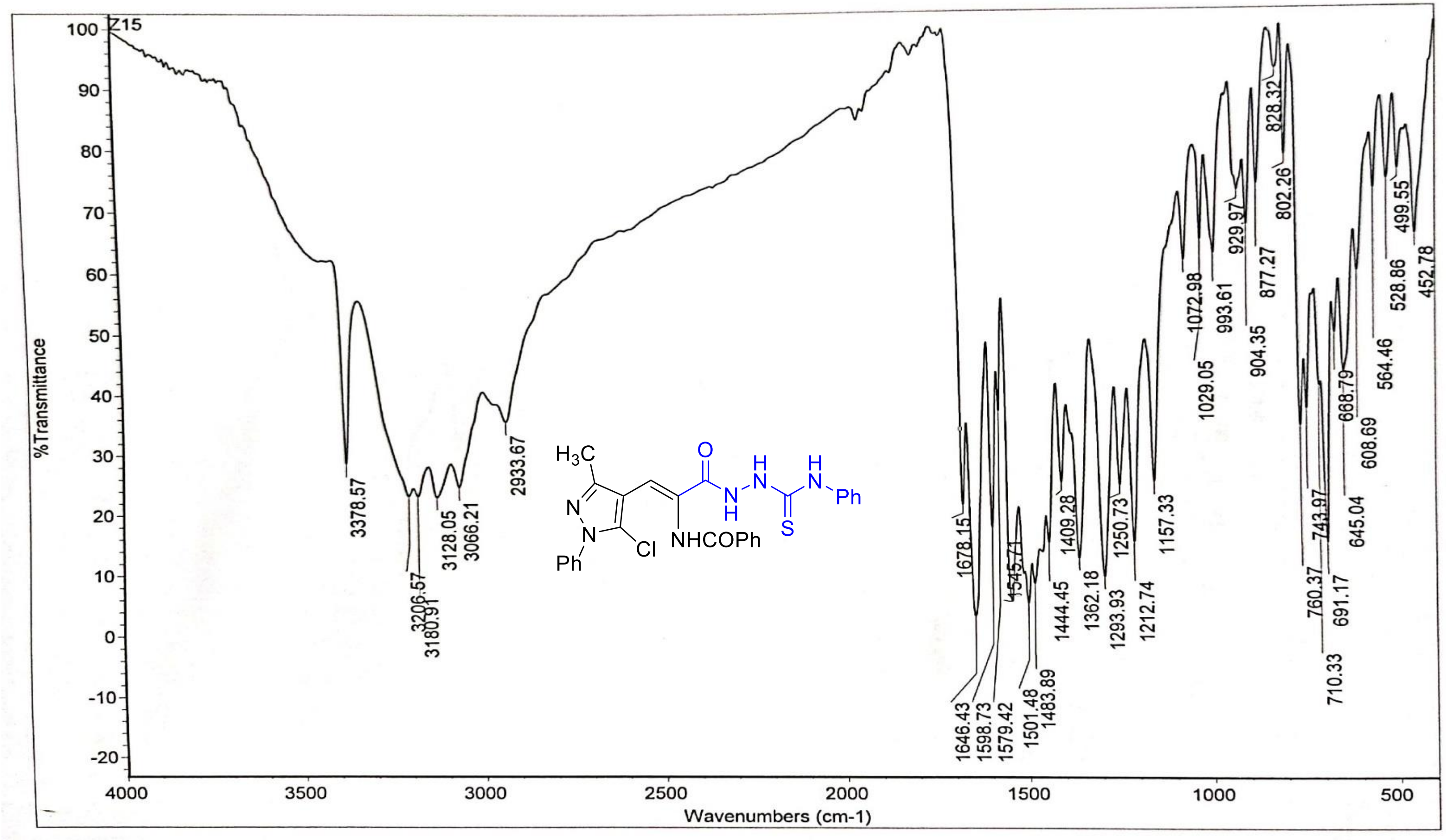

Fig. S29: IR spectrum of (9)

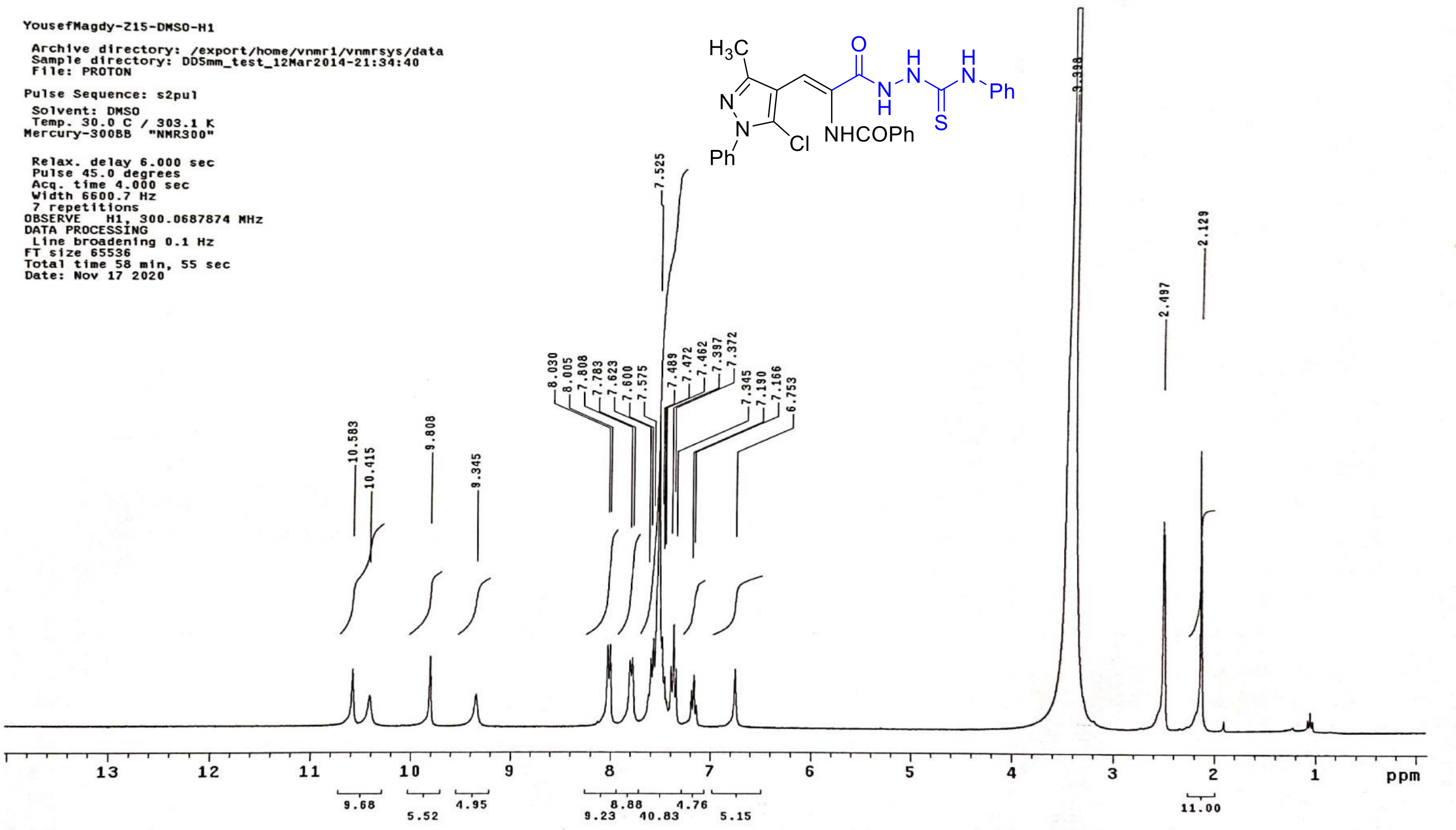

Fig. S30: ^{1}H NMR spectrum of (9)

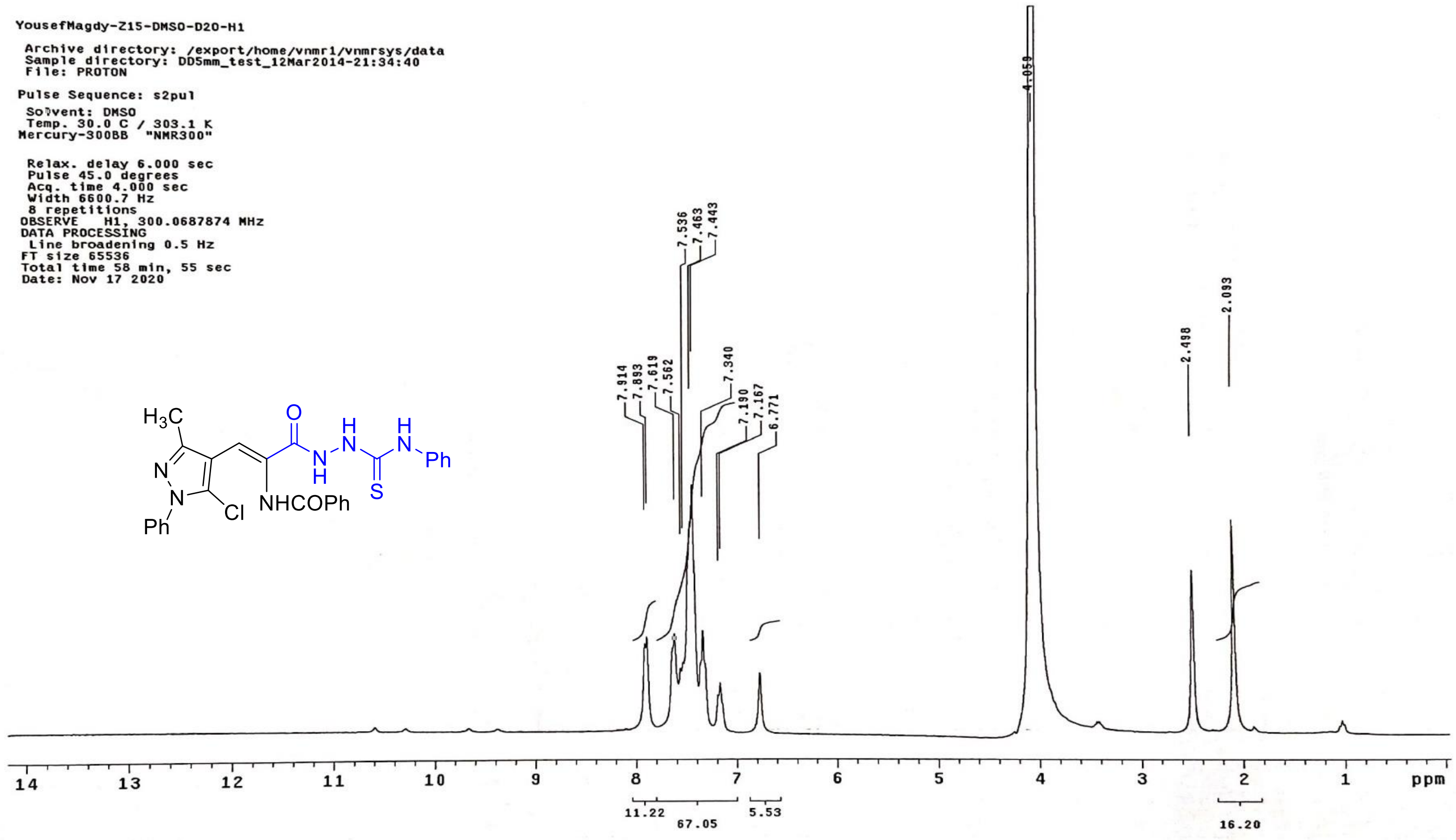

Fig. S31: ^{1}H NMR-D_2O spectrum of (9)

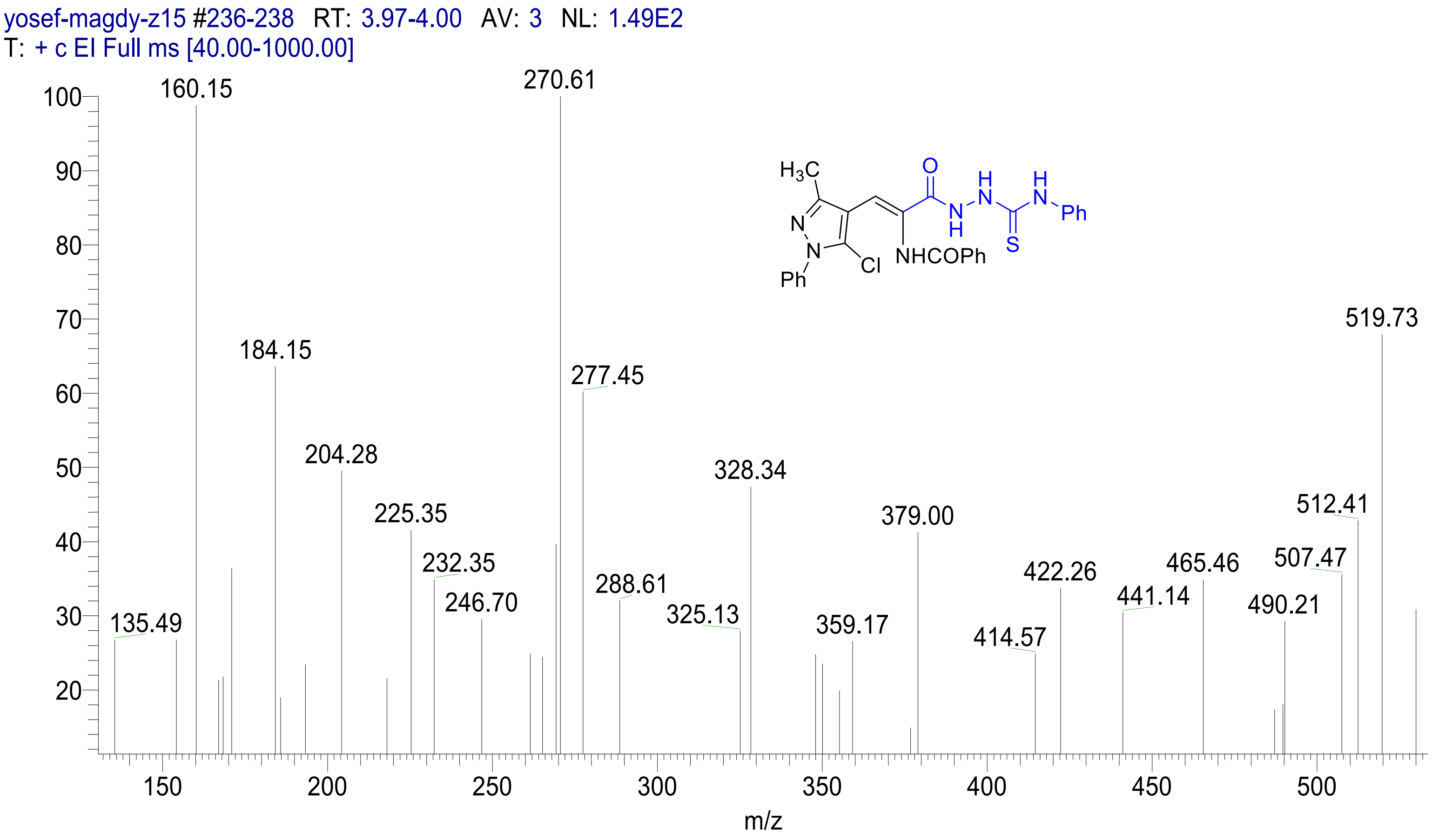

Fig. S32: Mass spectrum of (9)

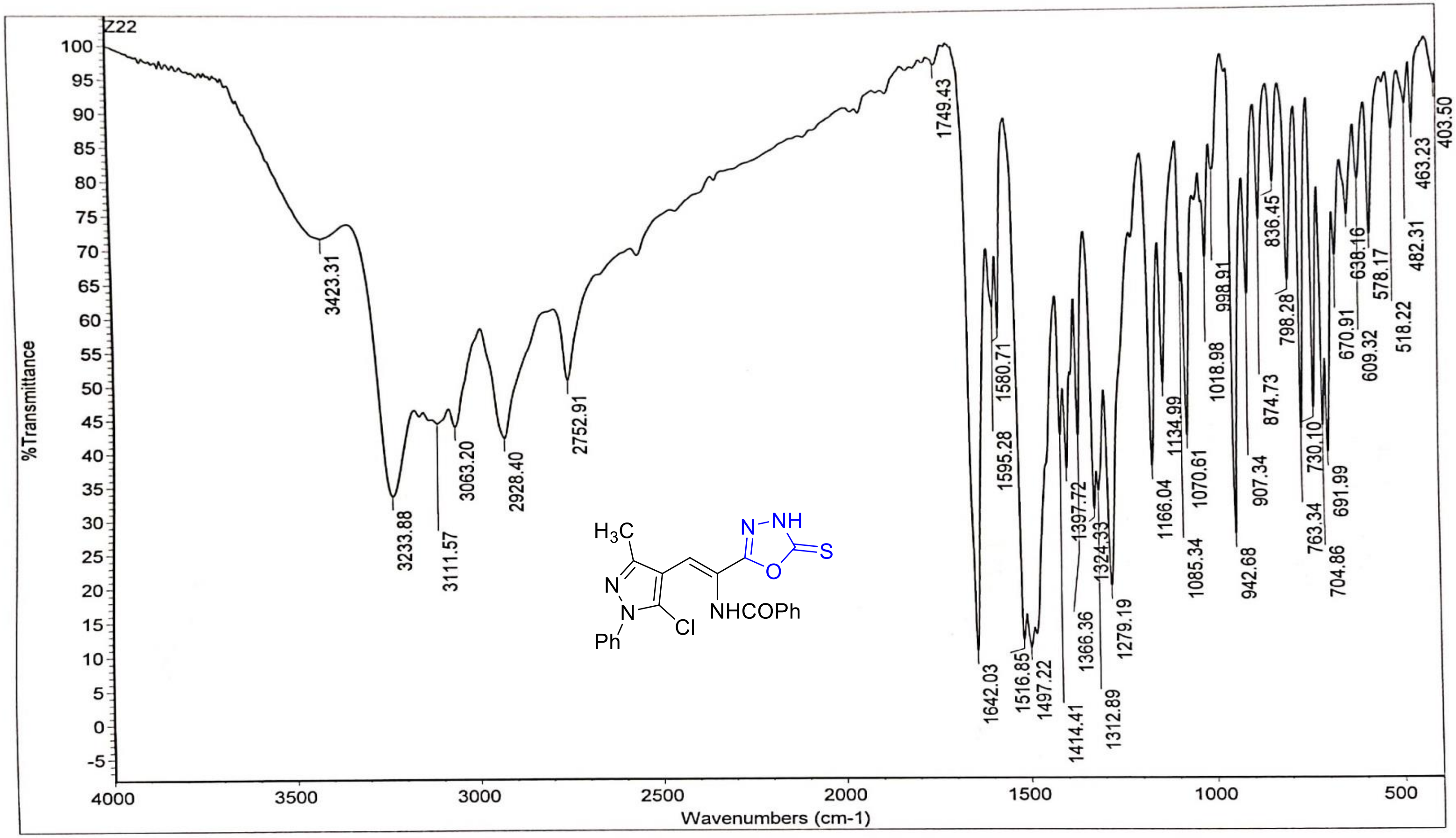

Fig. S33: IR spectrum of (10)

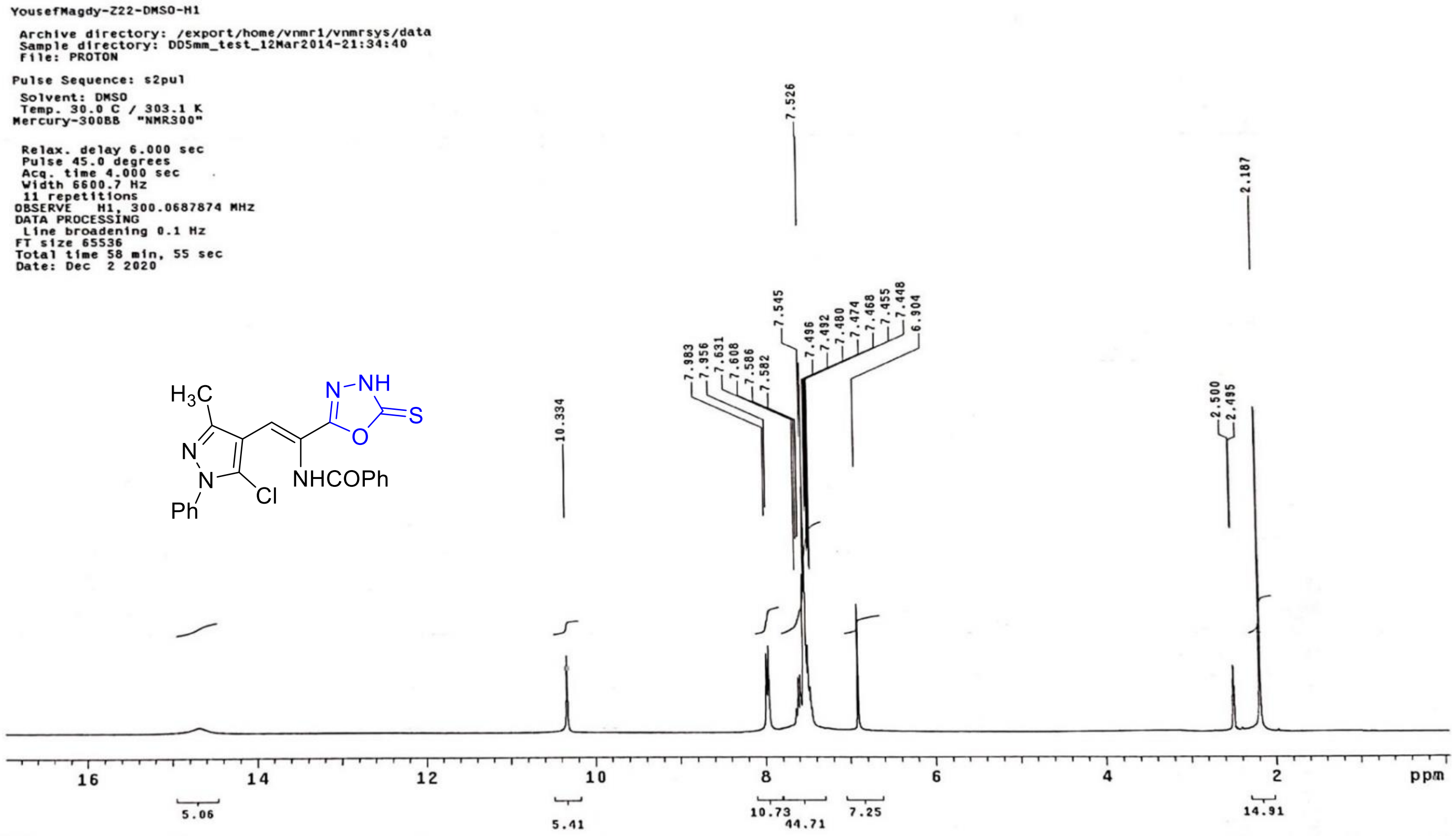

Fig. S34: ^{1}H NMR spectrum of (10)

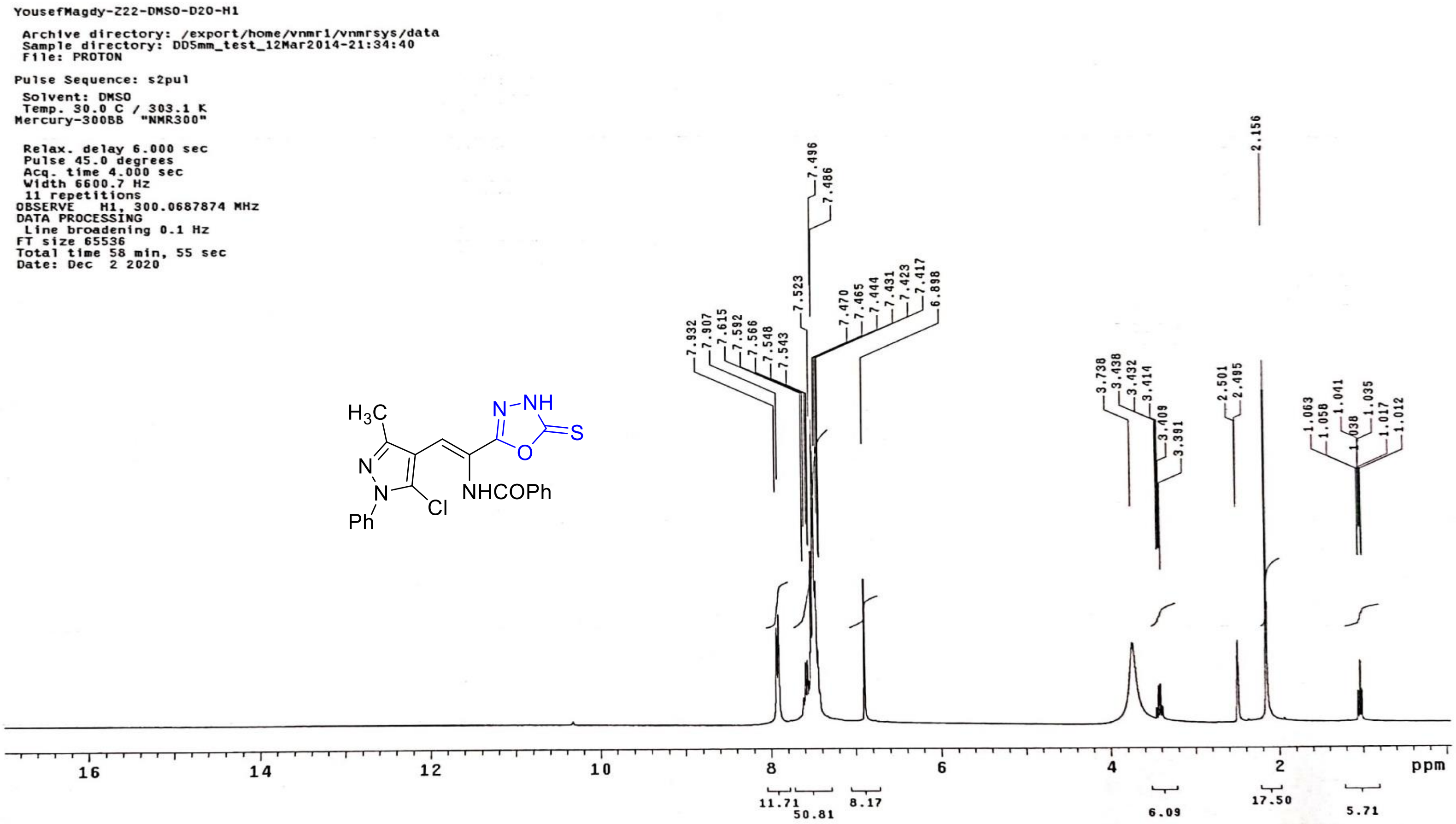

Fig. S35: ^{1}H NMR-D_2O spectrum of (10)

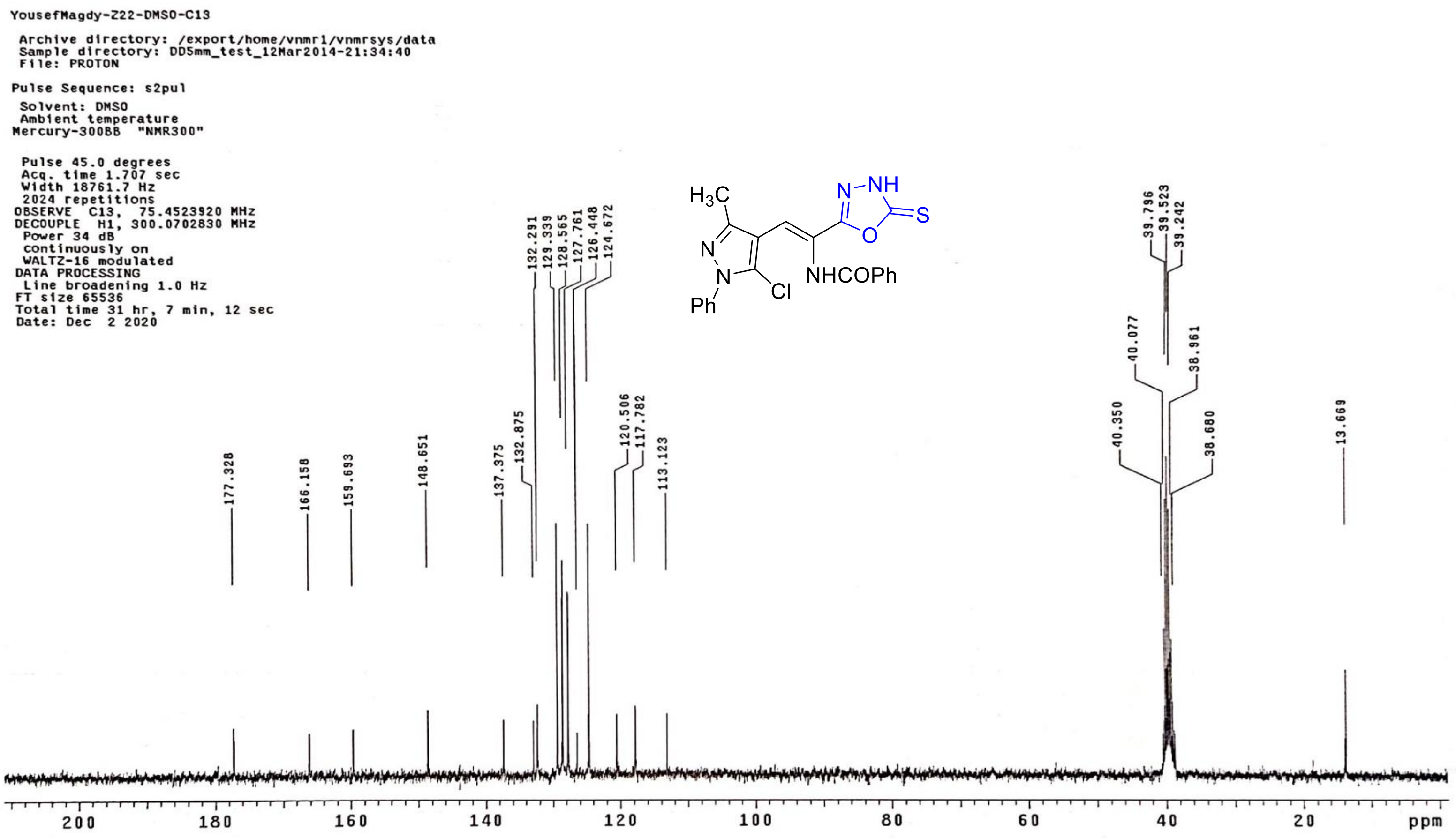

Fig. S36: ^{13}CNMR spectrum of (10)

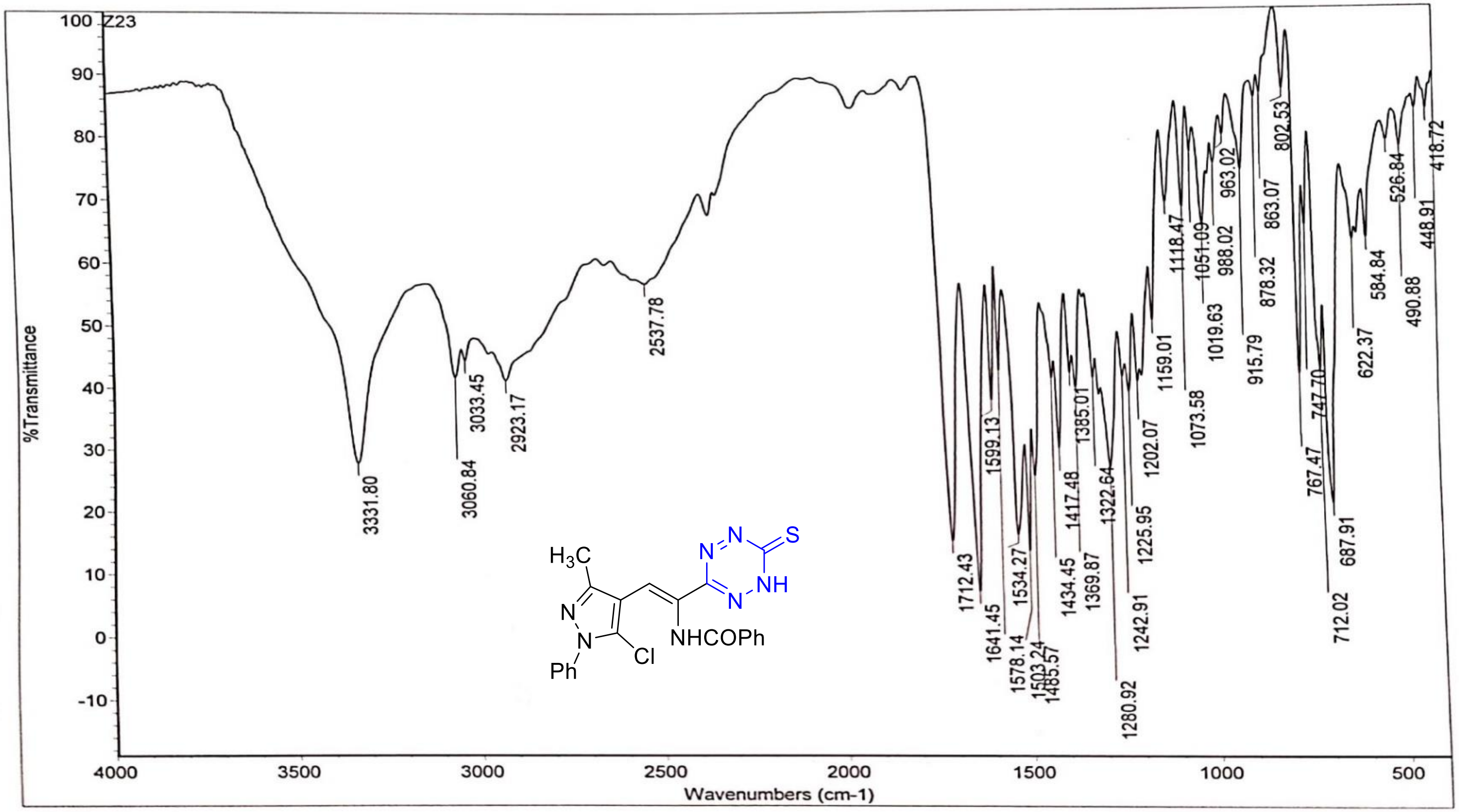

Fig. S37: IR spectrum of (11)

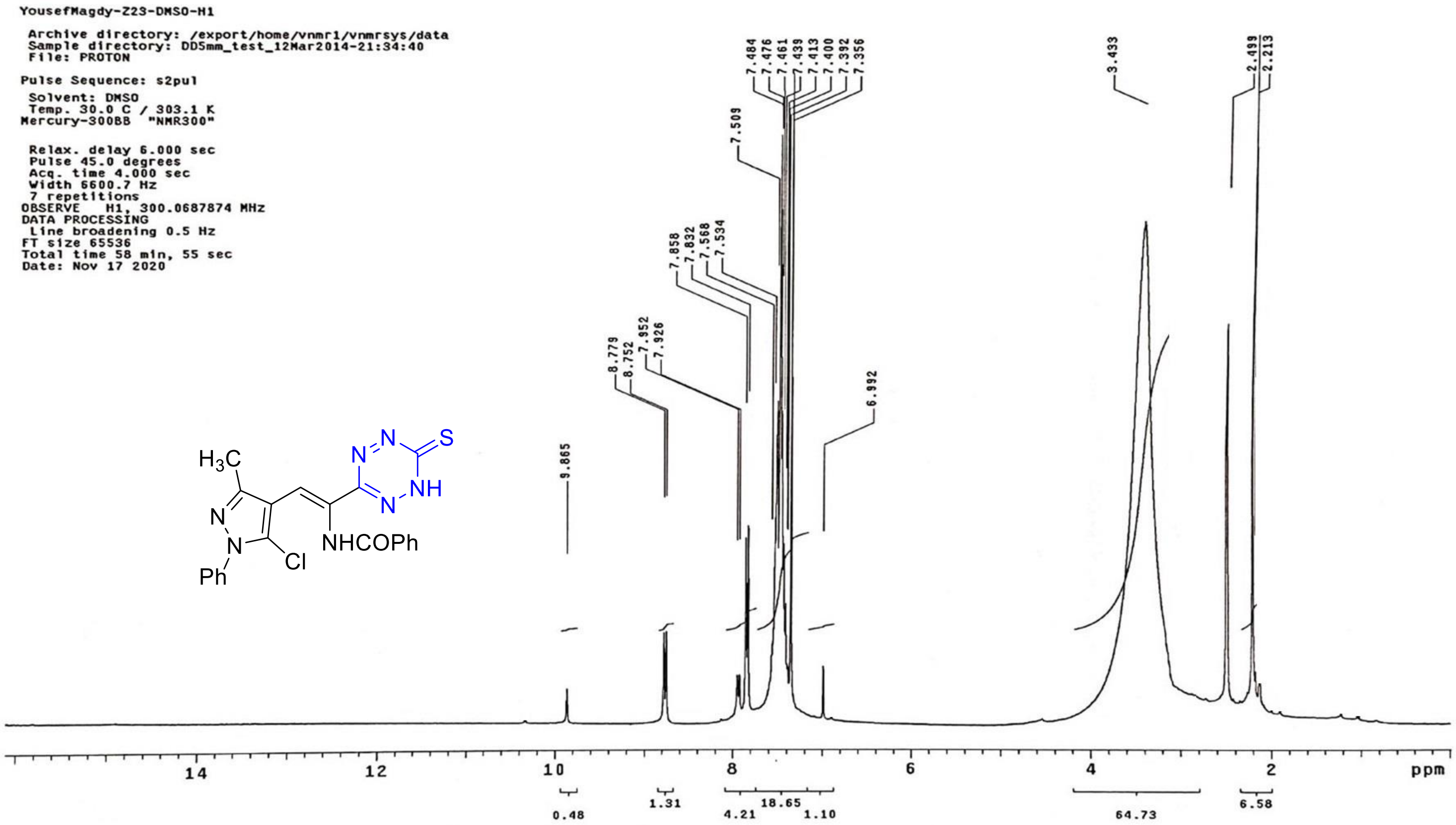

Fig. S38: ^{1}H NMR spectrum of (11)

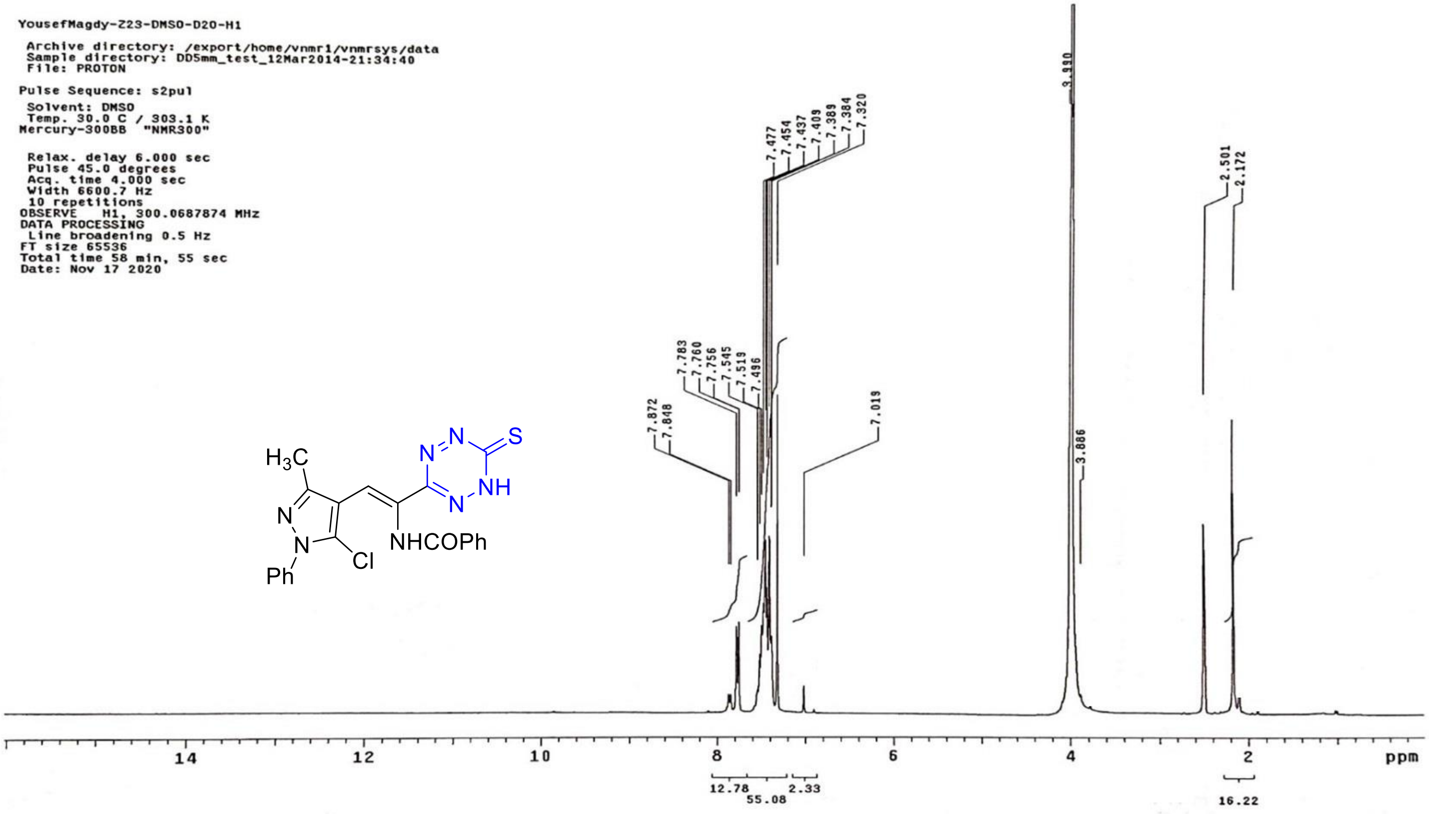

Fig. S39: ^{1}H NMR-D_2O spectrum of (11)

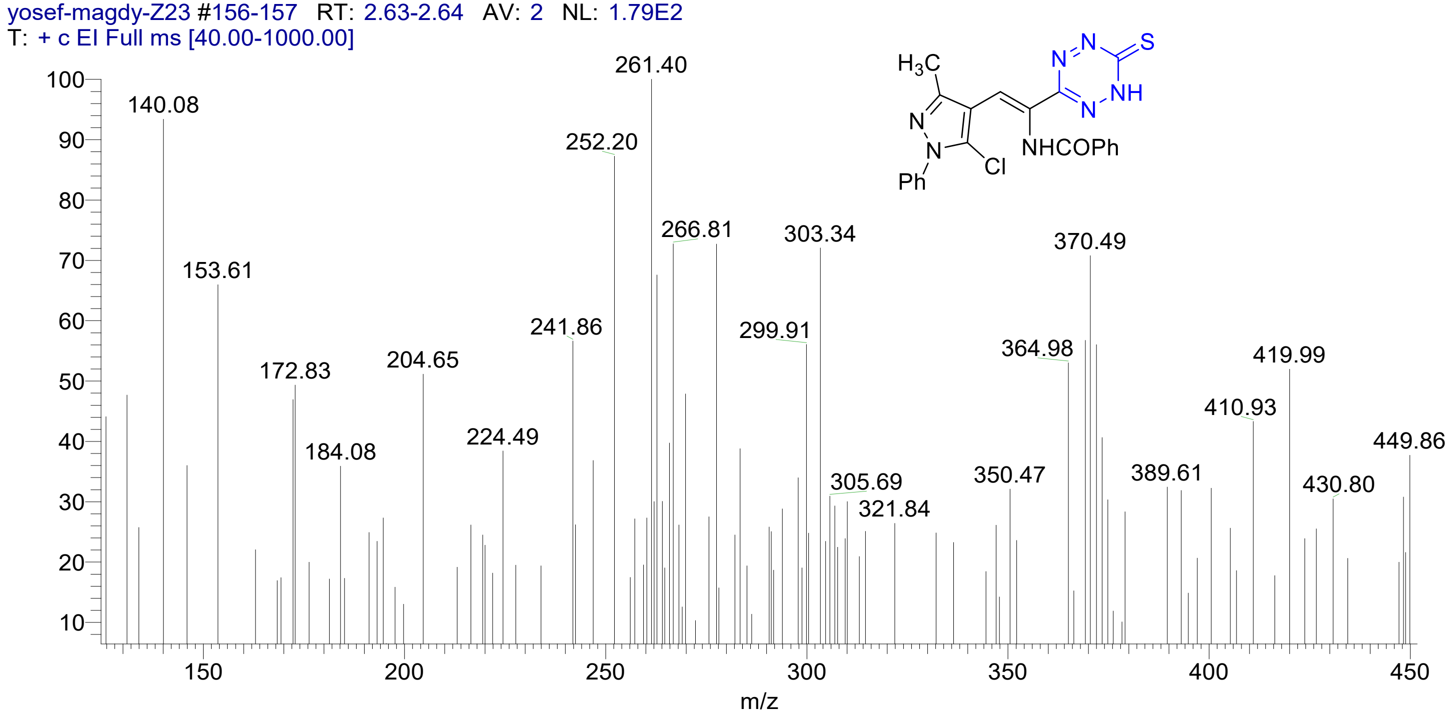

Fig. S40: Mass spectrum of (11)

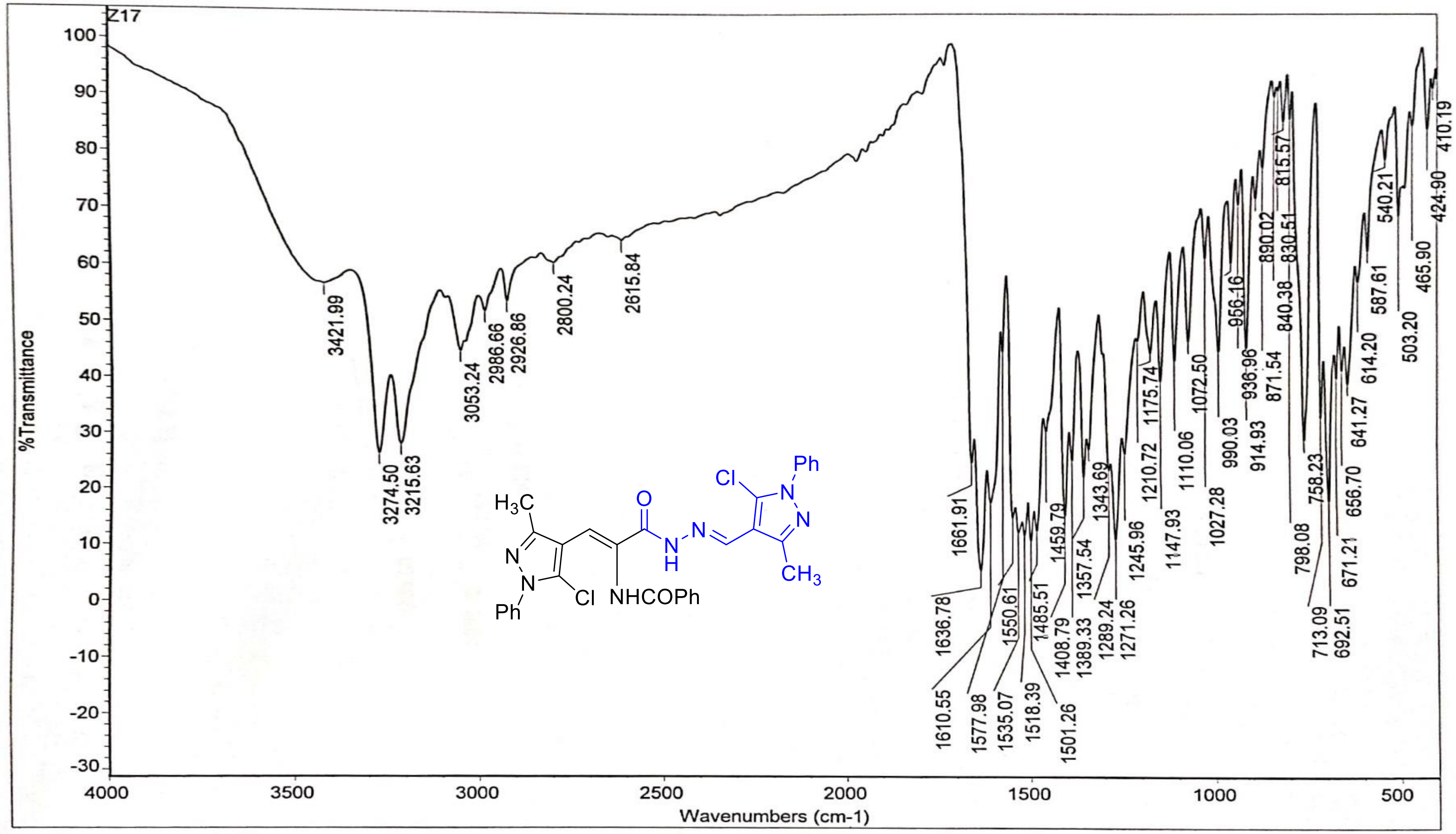

Fig. S41: IR spectrum of (12)

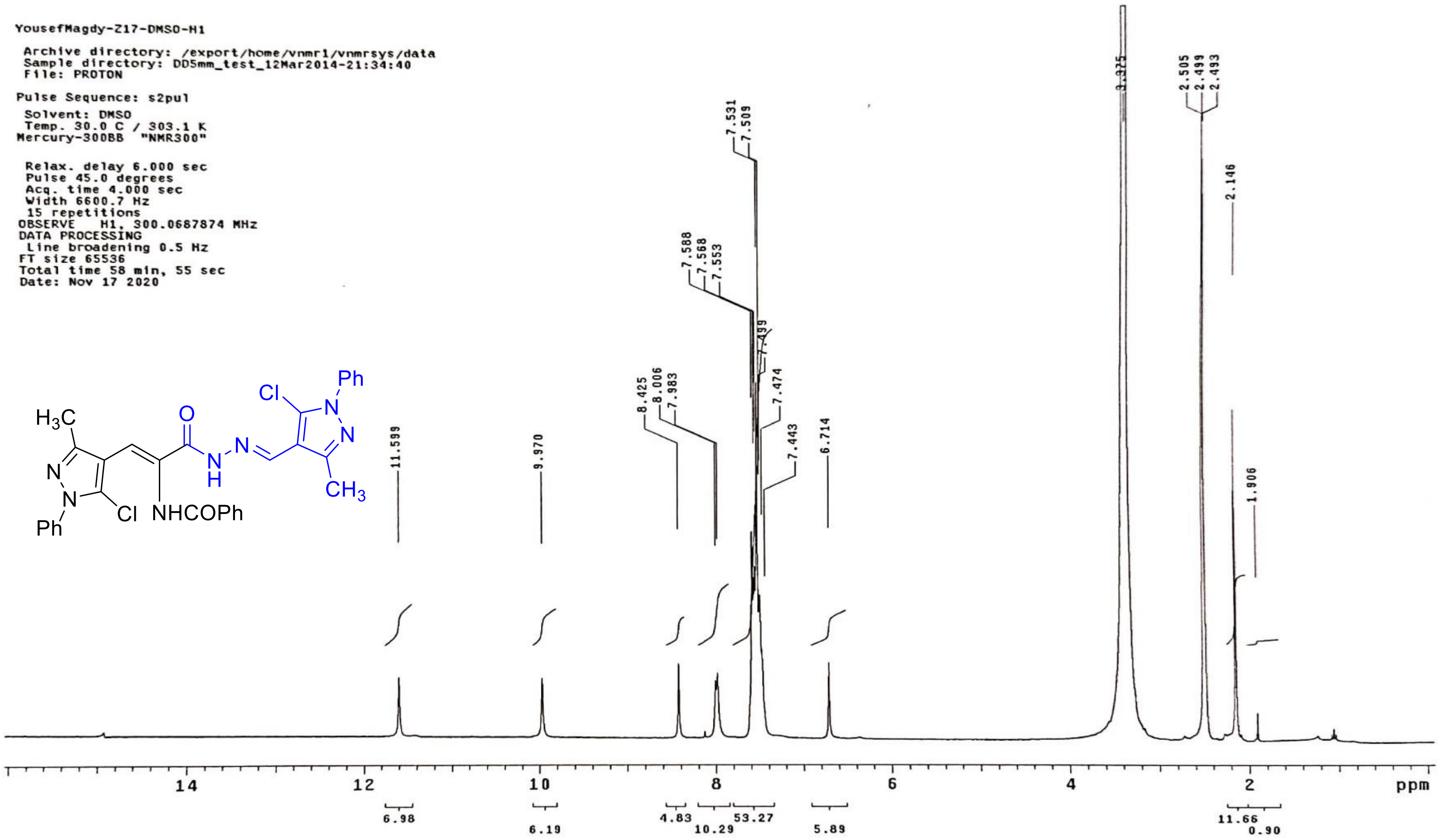

Fig. S42: ^{1}H NMR spectrum of (12)

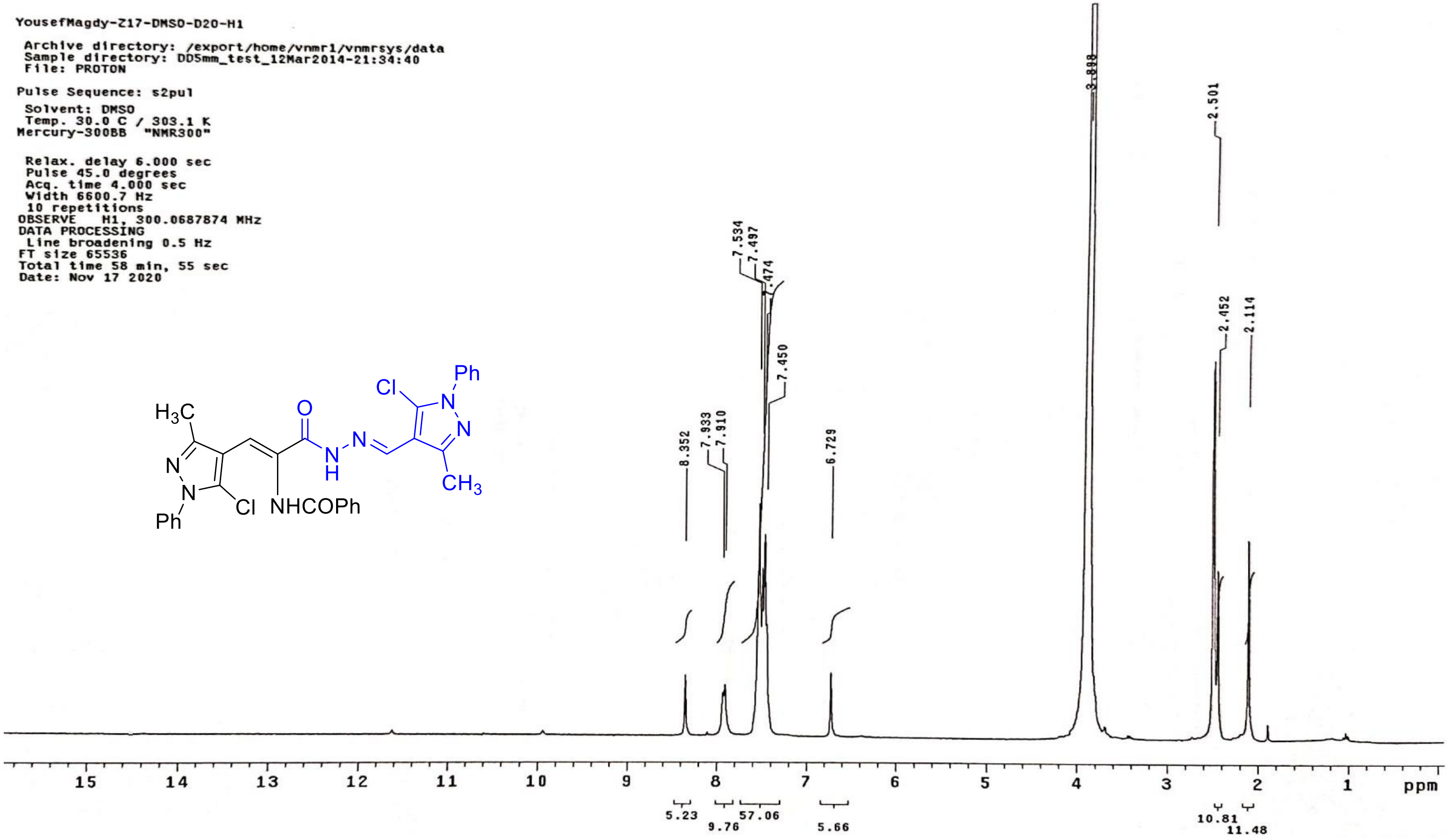

Fig. S43: ^{1}H NMR-D_2O spectrum of (12)

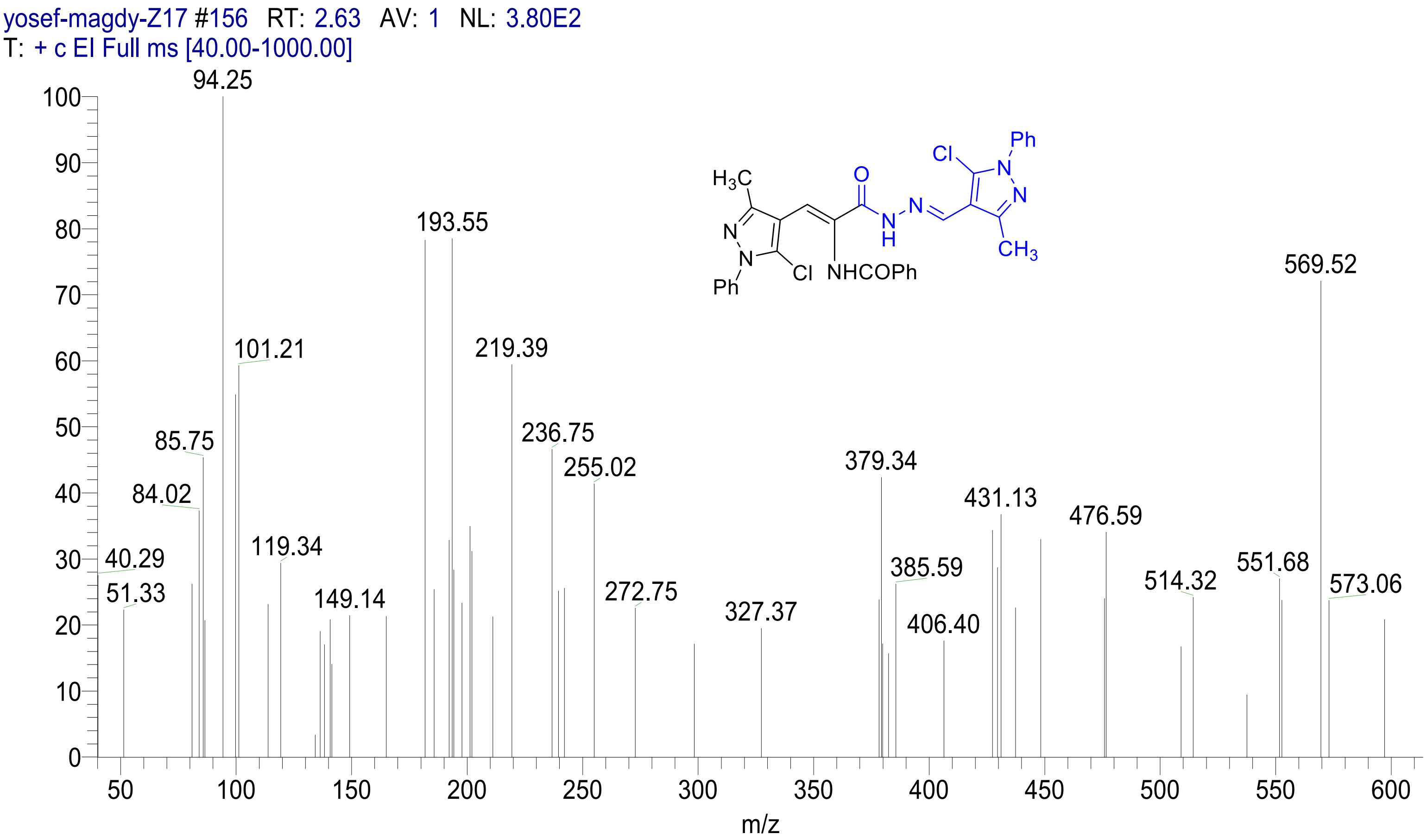

Fig. S44: Mass spectrum of (12)

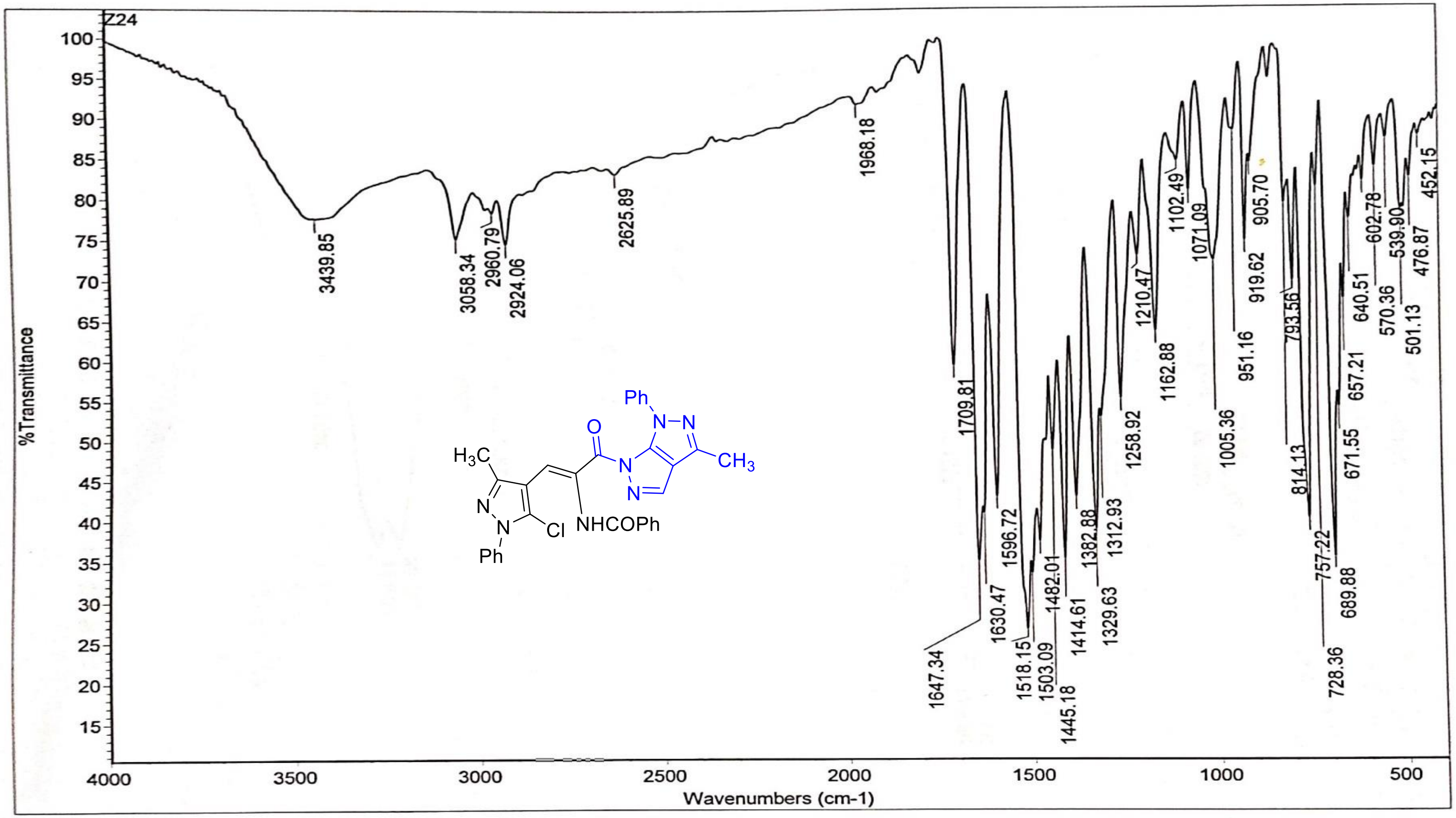

Fig. S45: IR spectrum of (13)

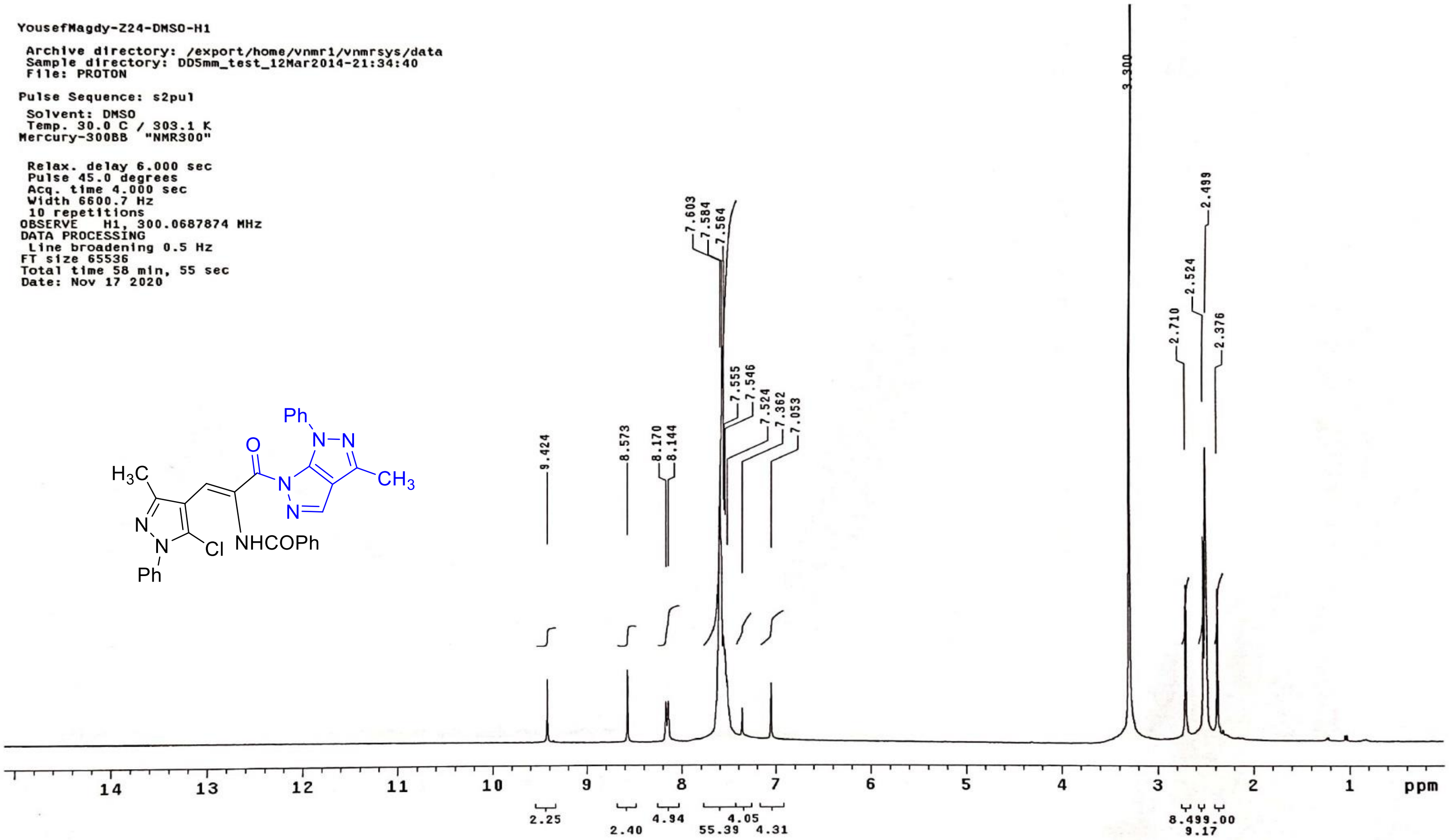

Fig. S46: ^{1}H NMR spectrum of (13)

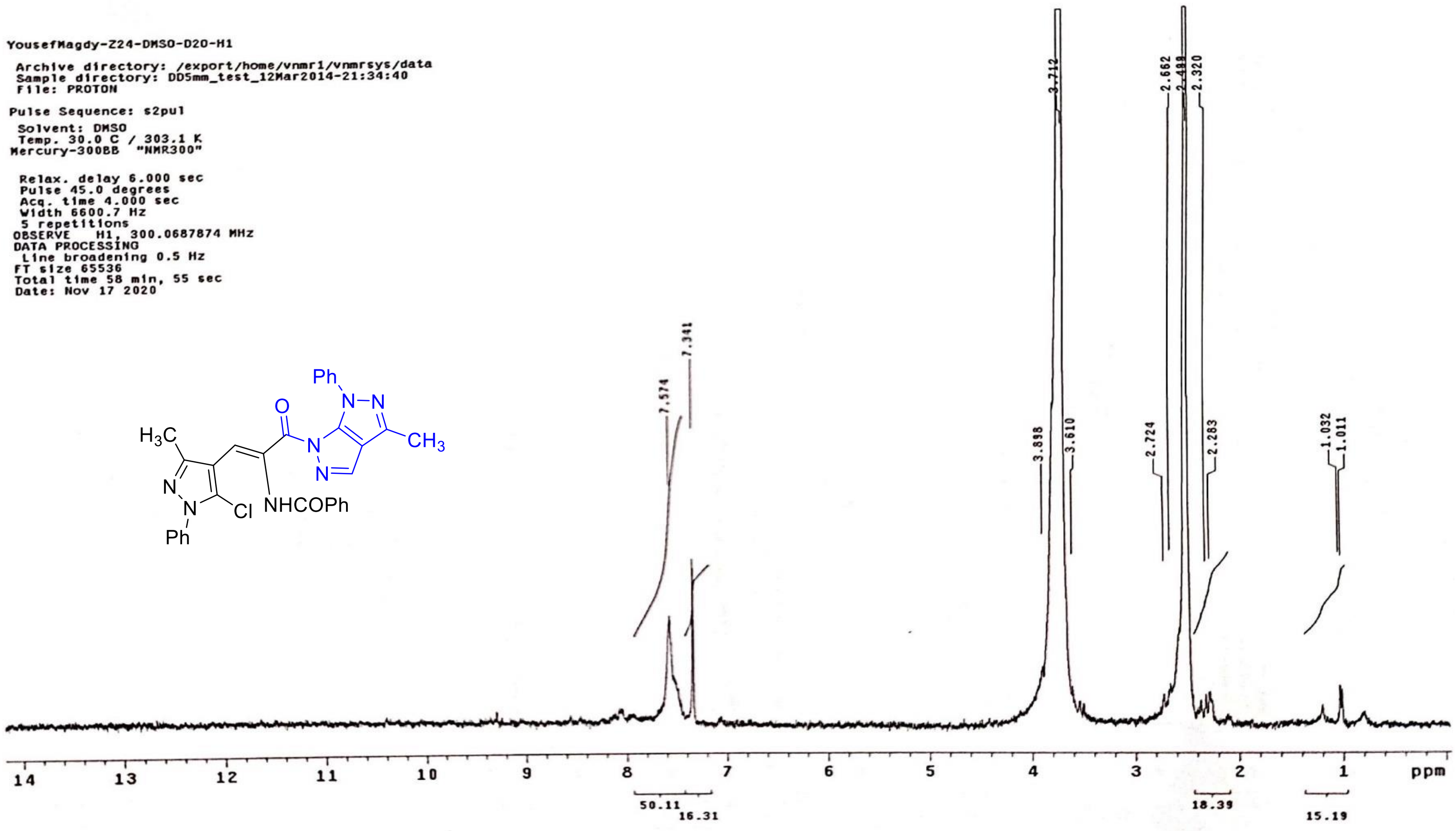

Fig. S47: ^{1}H NMR-D_2O spectrum of (13)

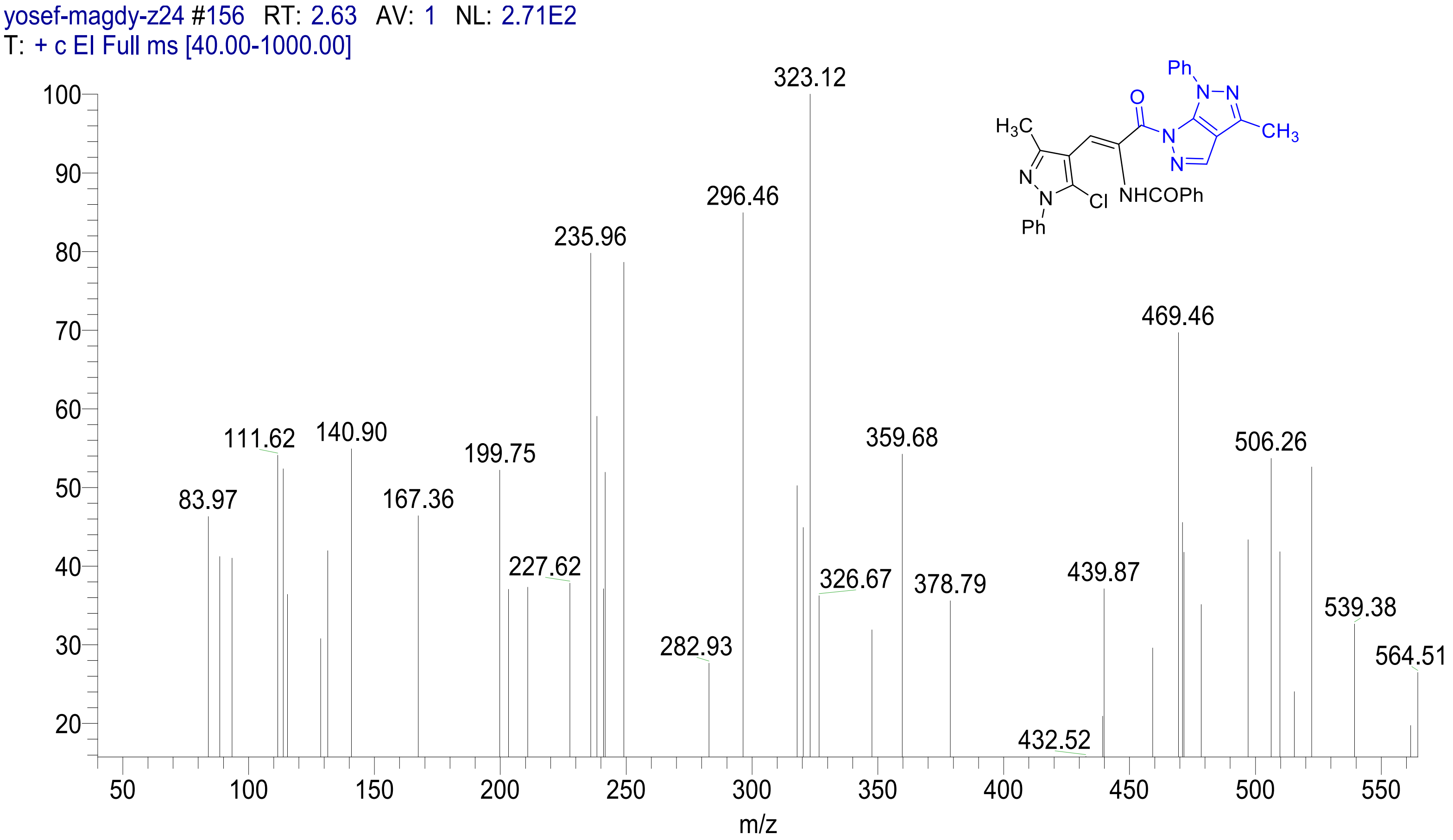

Fig. S48: Mass spectrum of (13)

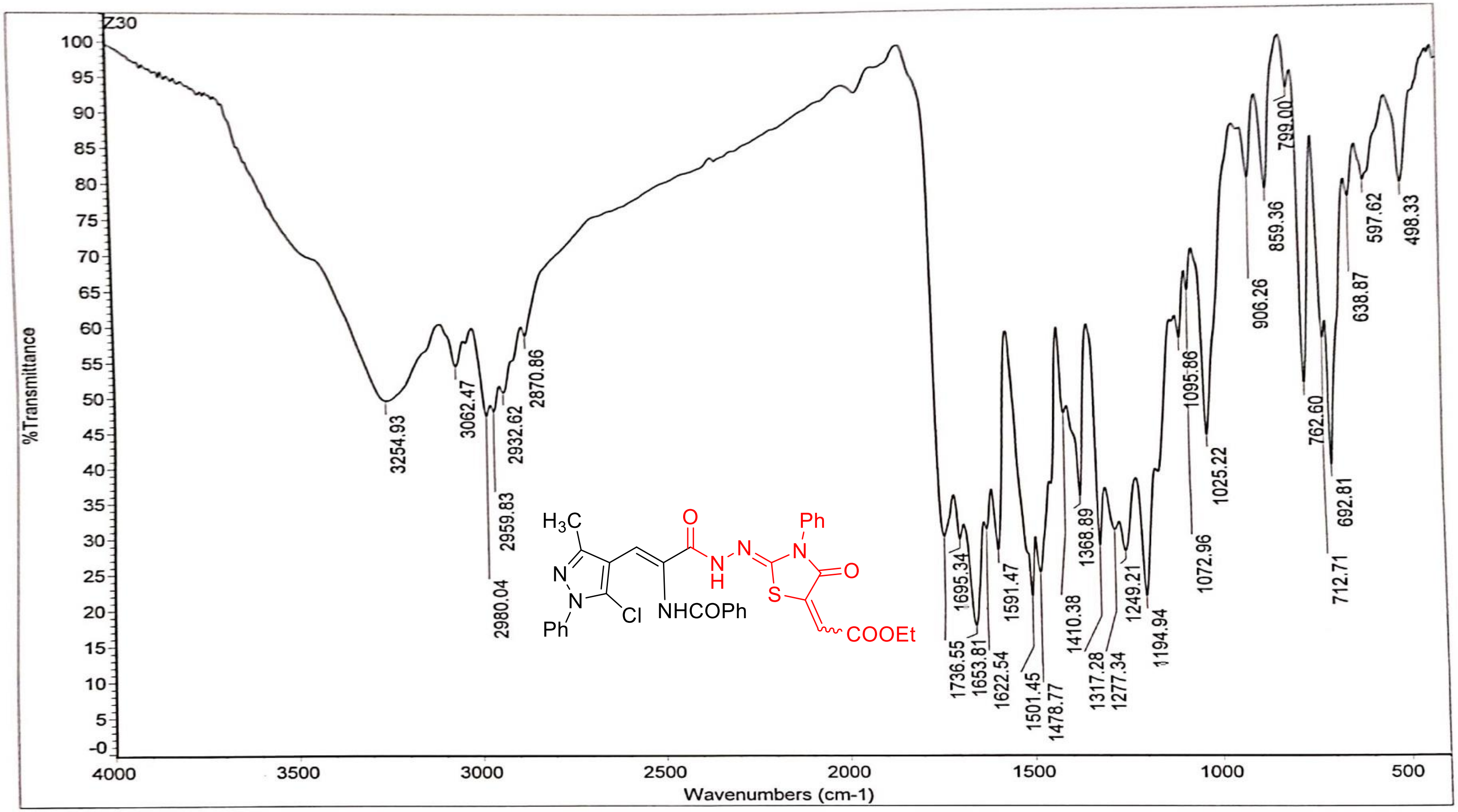

Fig. S49: IR spectrum of (14)

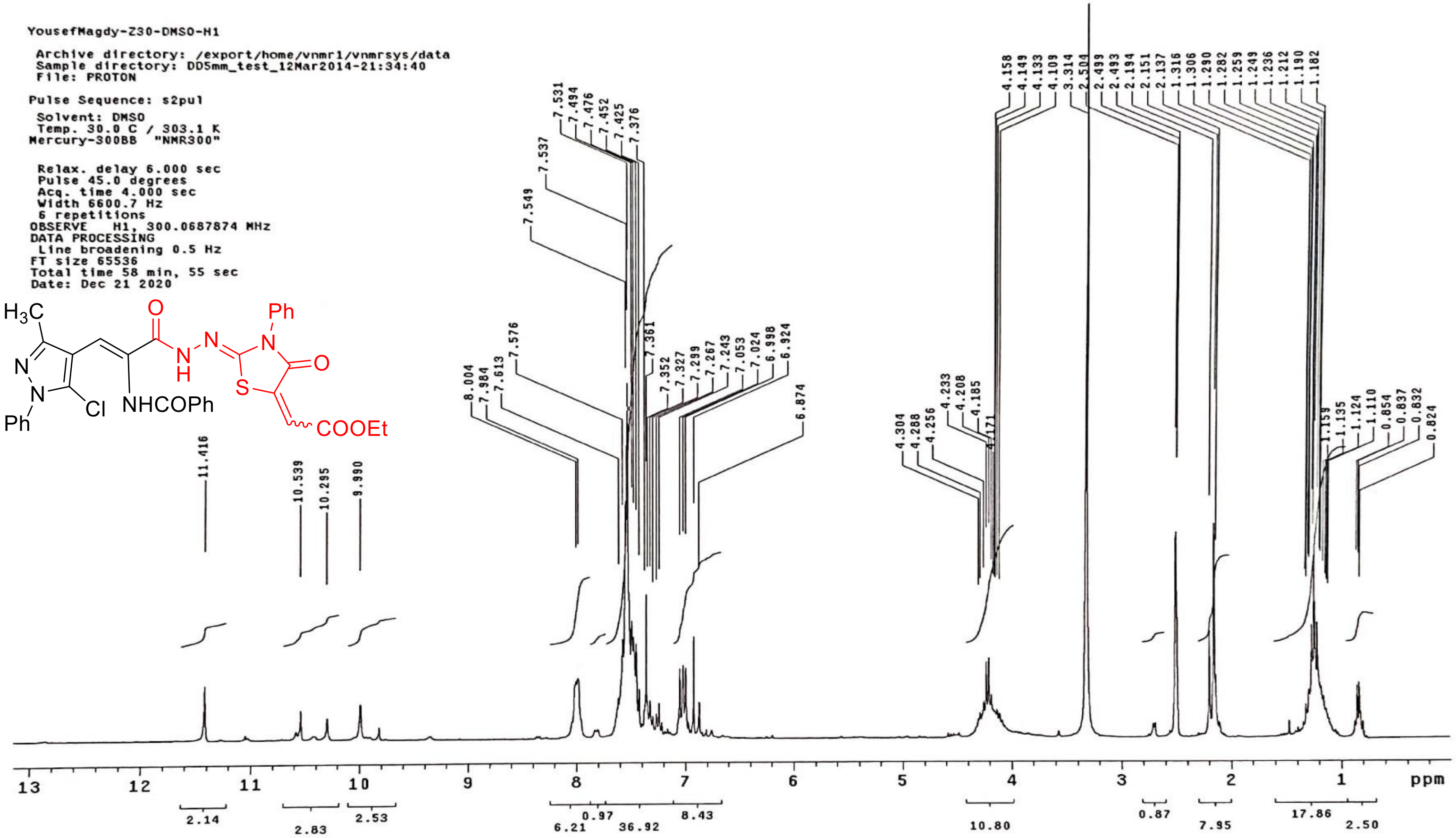

Fig. S50: ^{1}H NMR spectrum of (14)

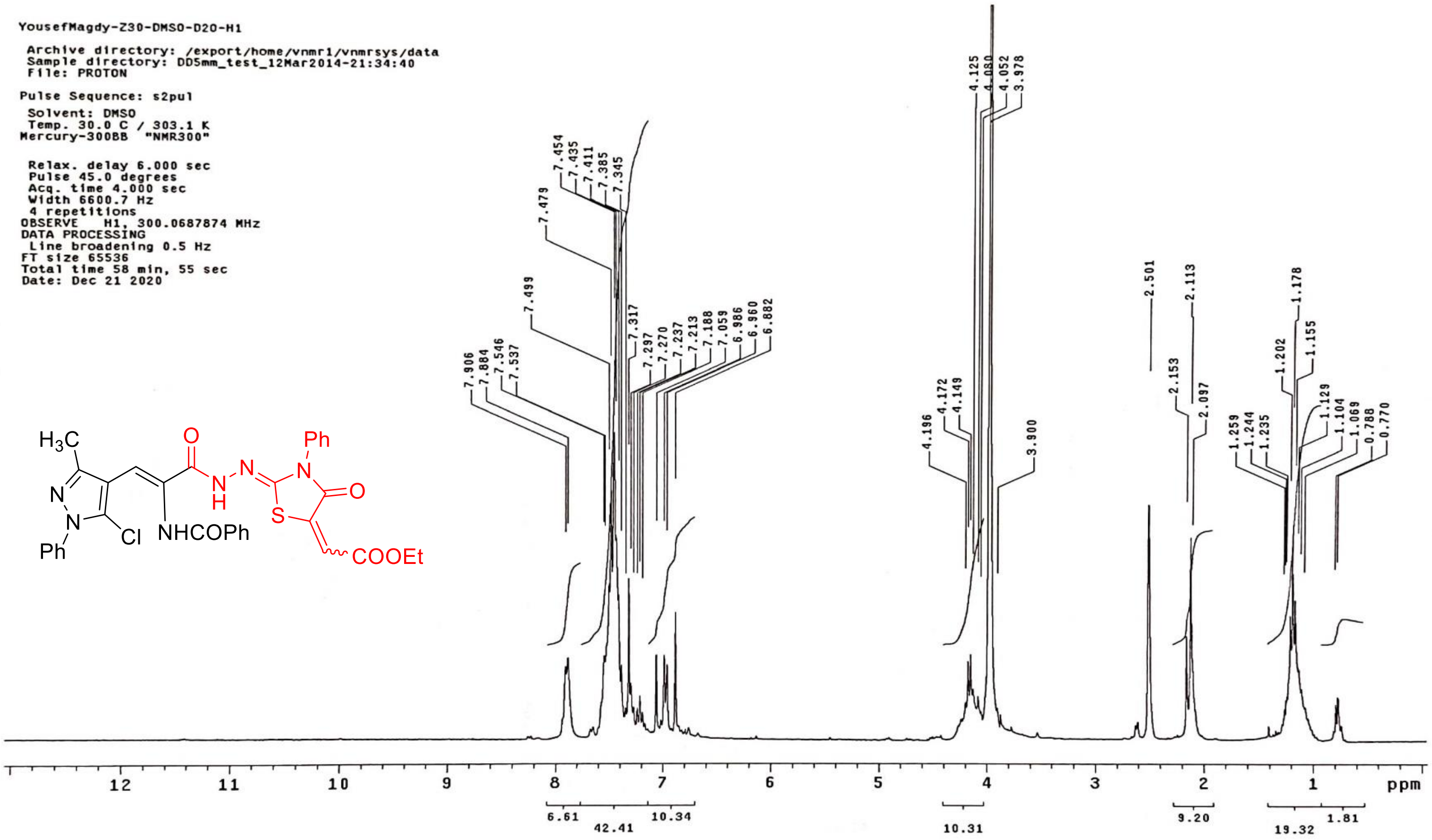

Fig. S51: ^{1}H NMR-D_2O spectrum of (14)

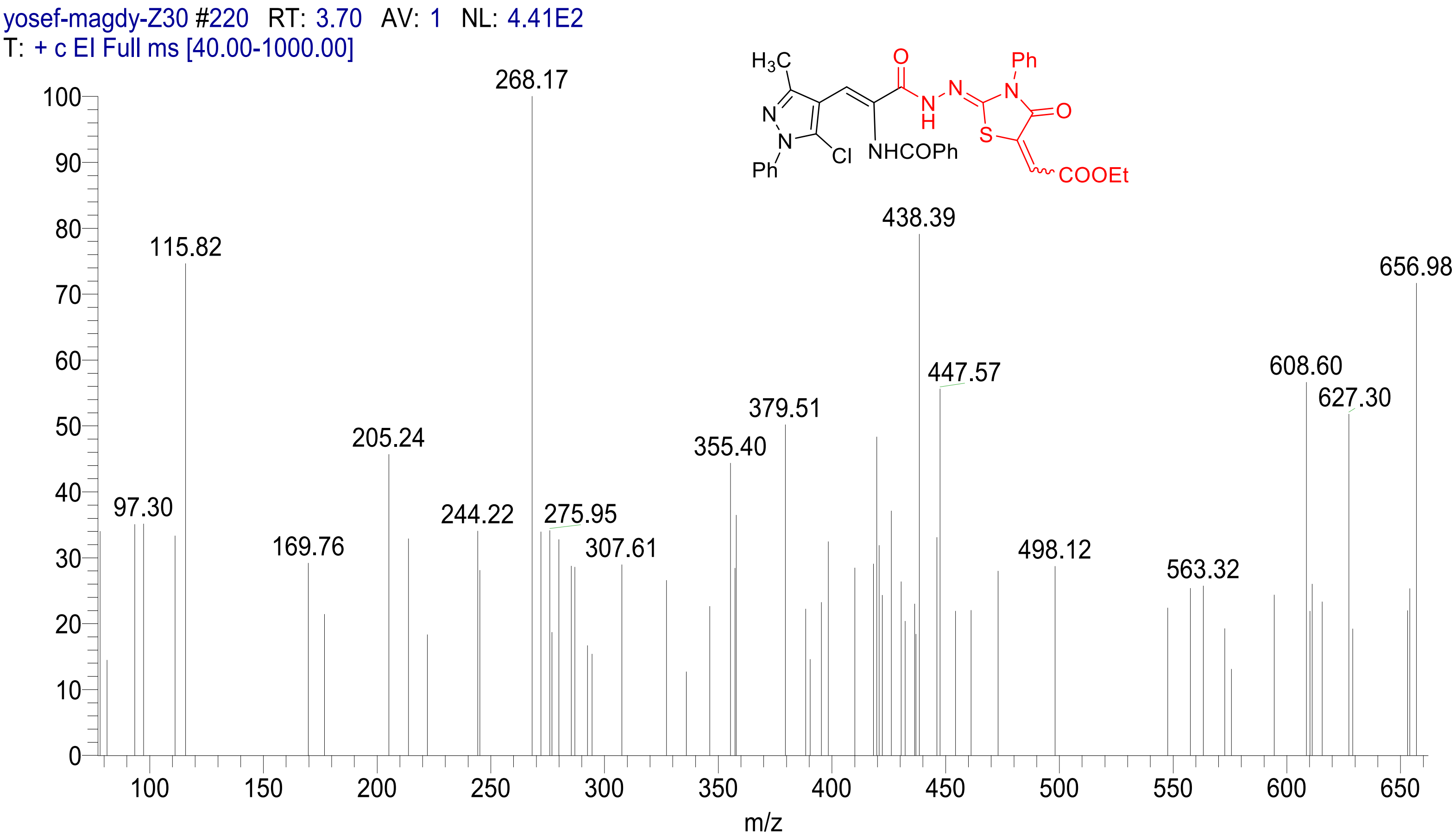

Fig. S52: Mass spectrum of (14)

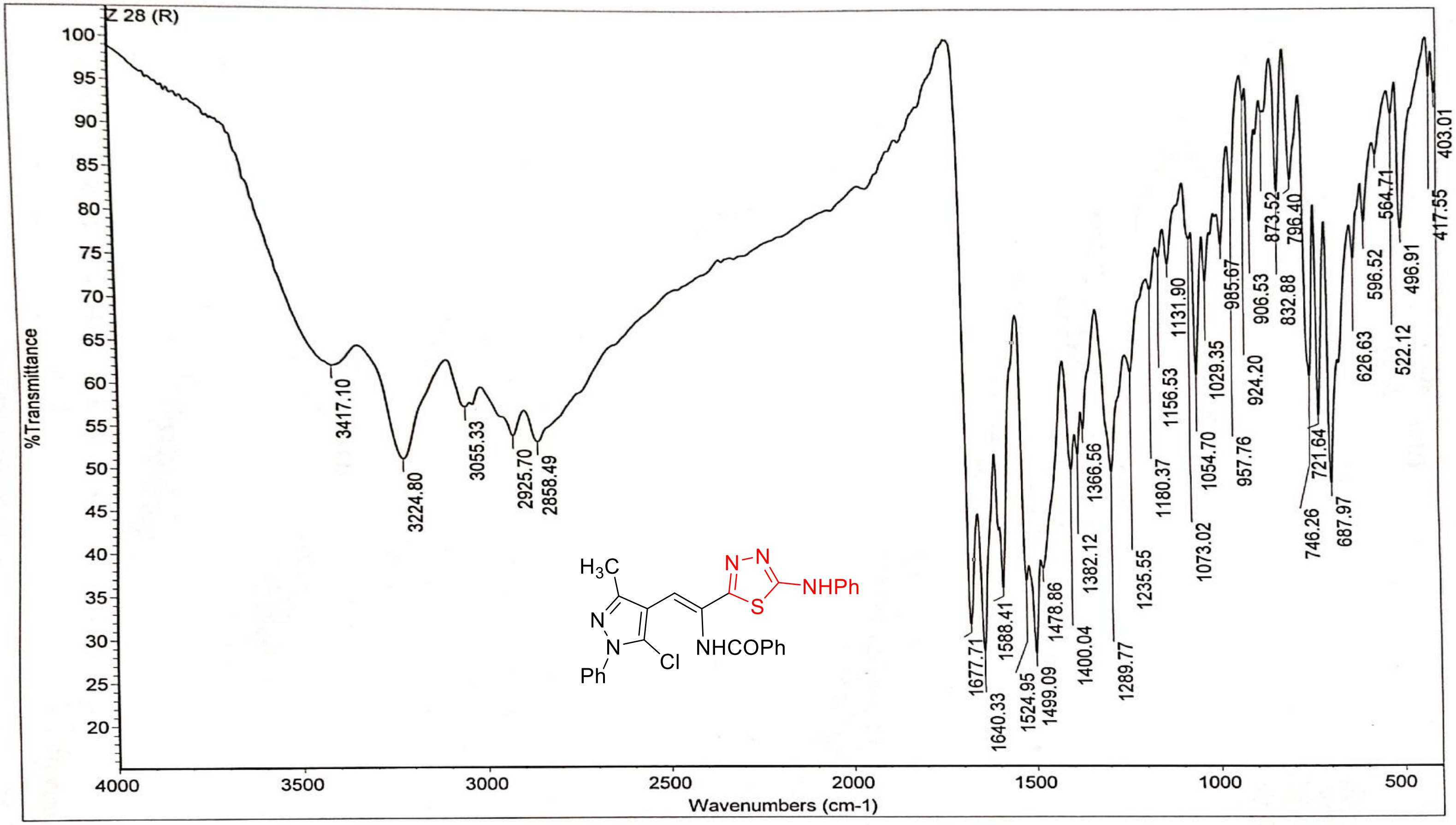

Fig. S53: IR spectrum of (16)

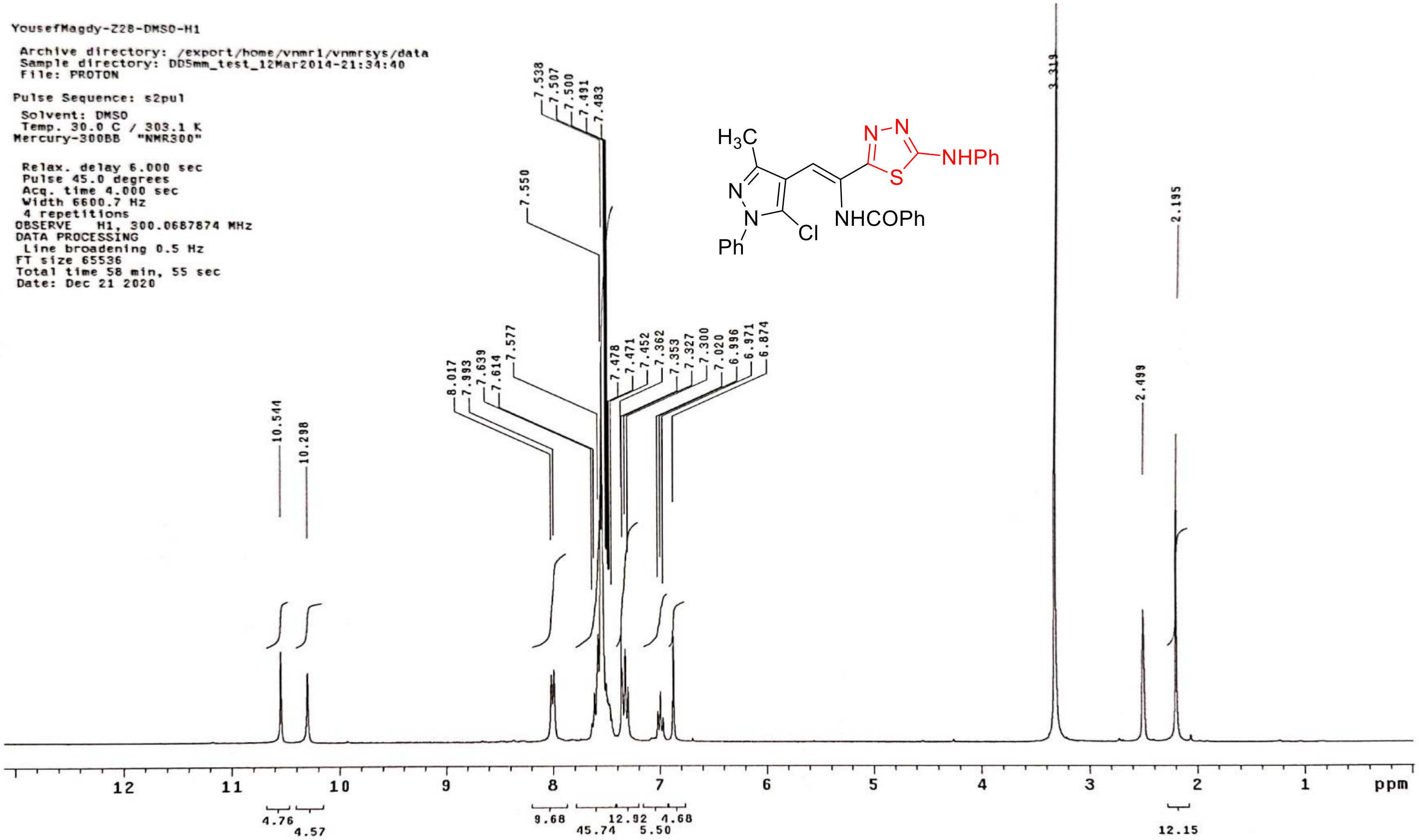

Fig. S54: ^{1}H NMR spectrum of (16)

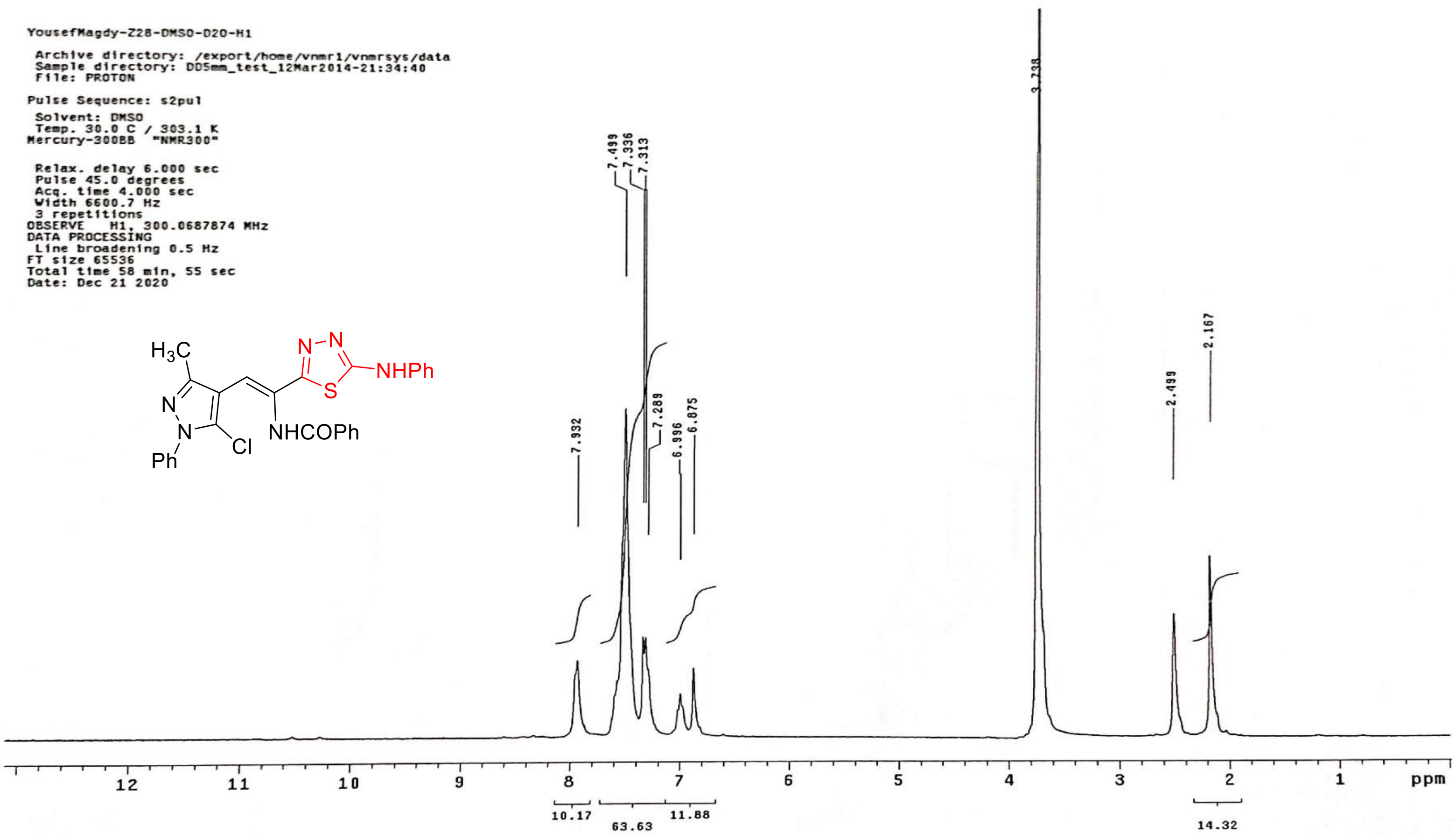

Fig. S55: ^{1}H NMR-D_2O spectrum of (16)

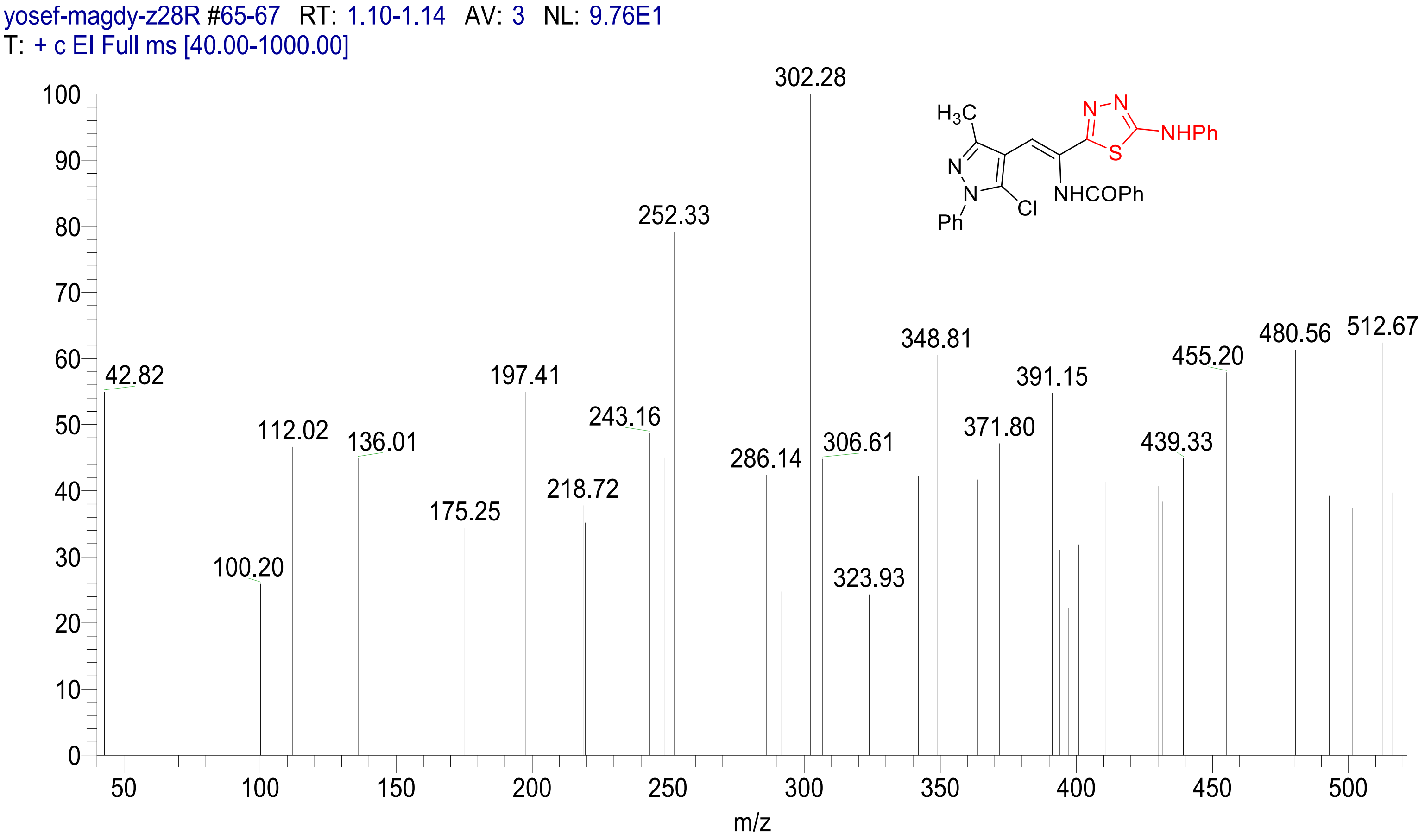

Fig. S56: Mass spectrum of (16)
